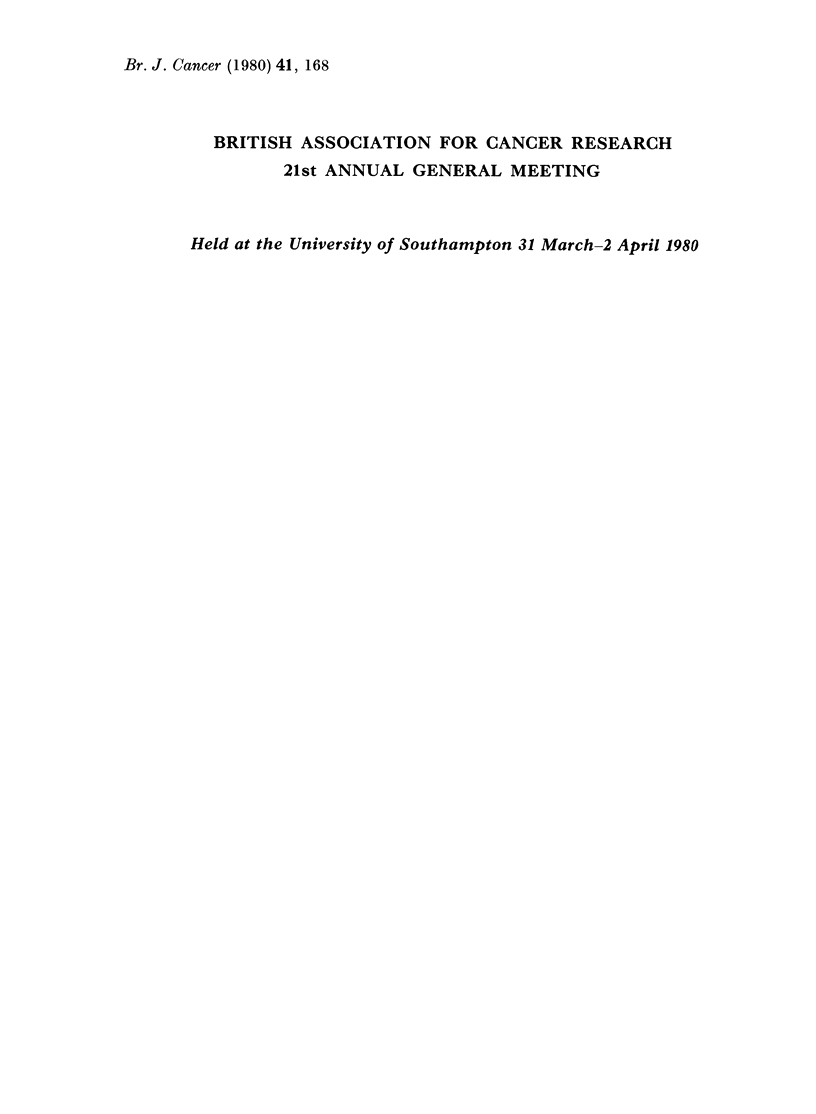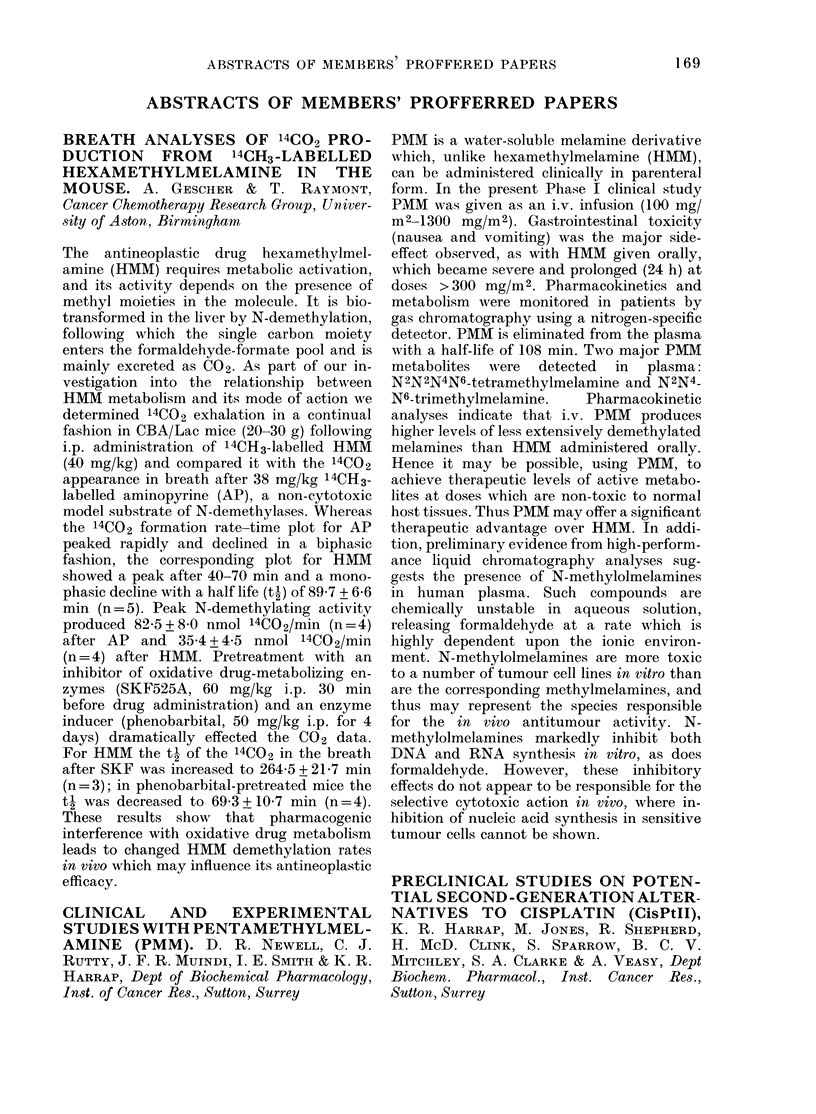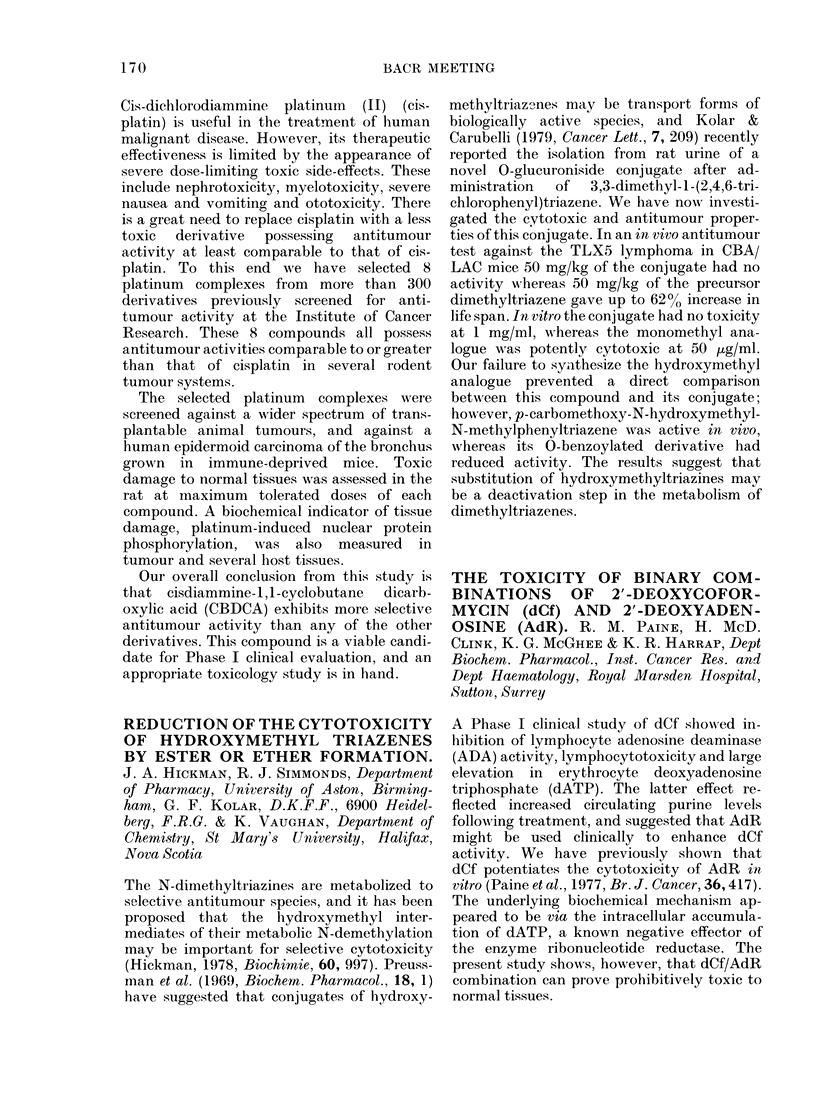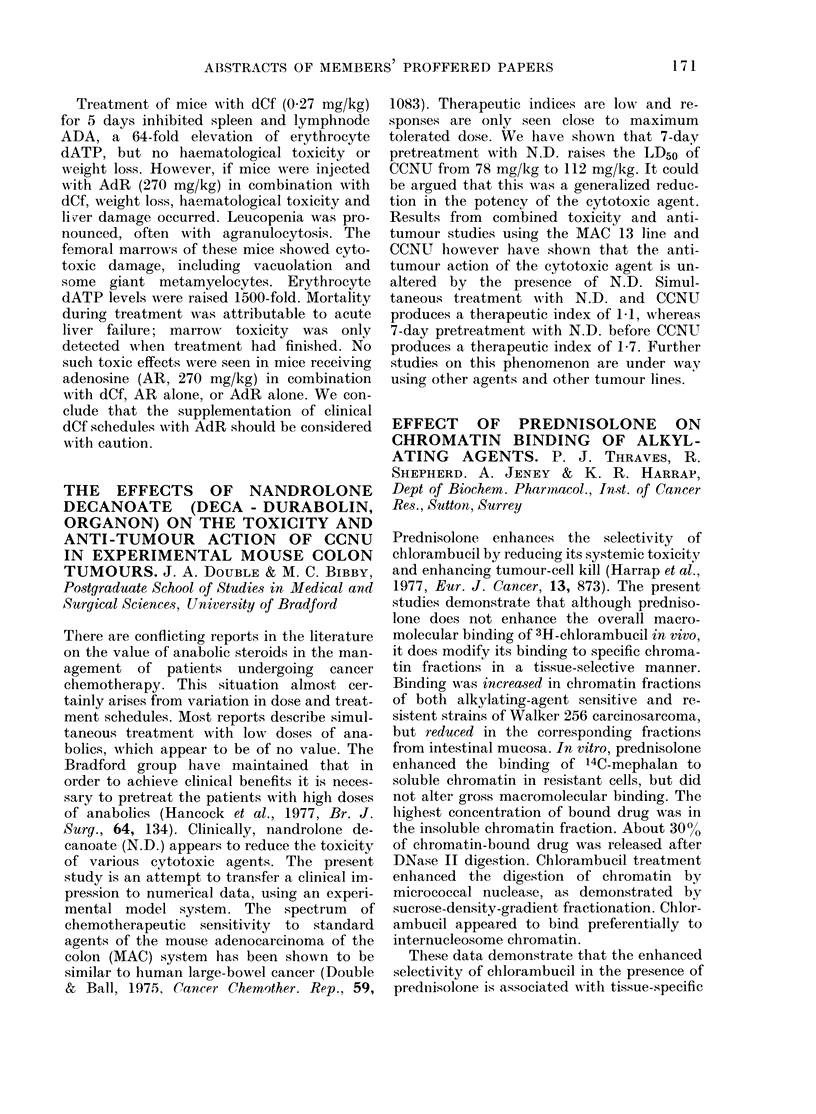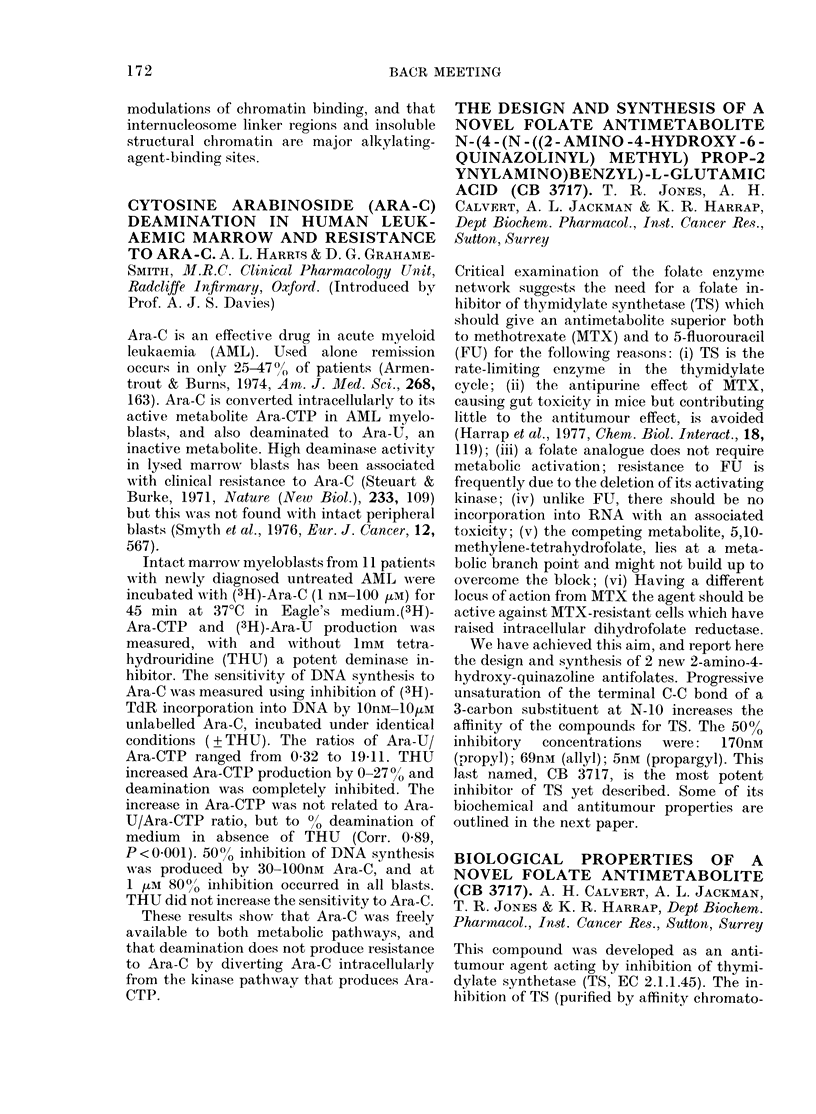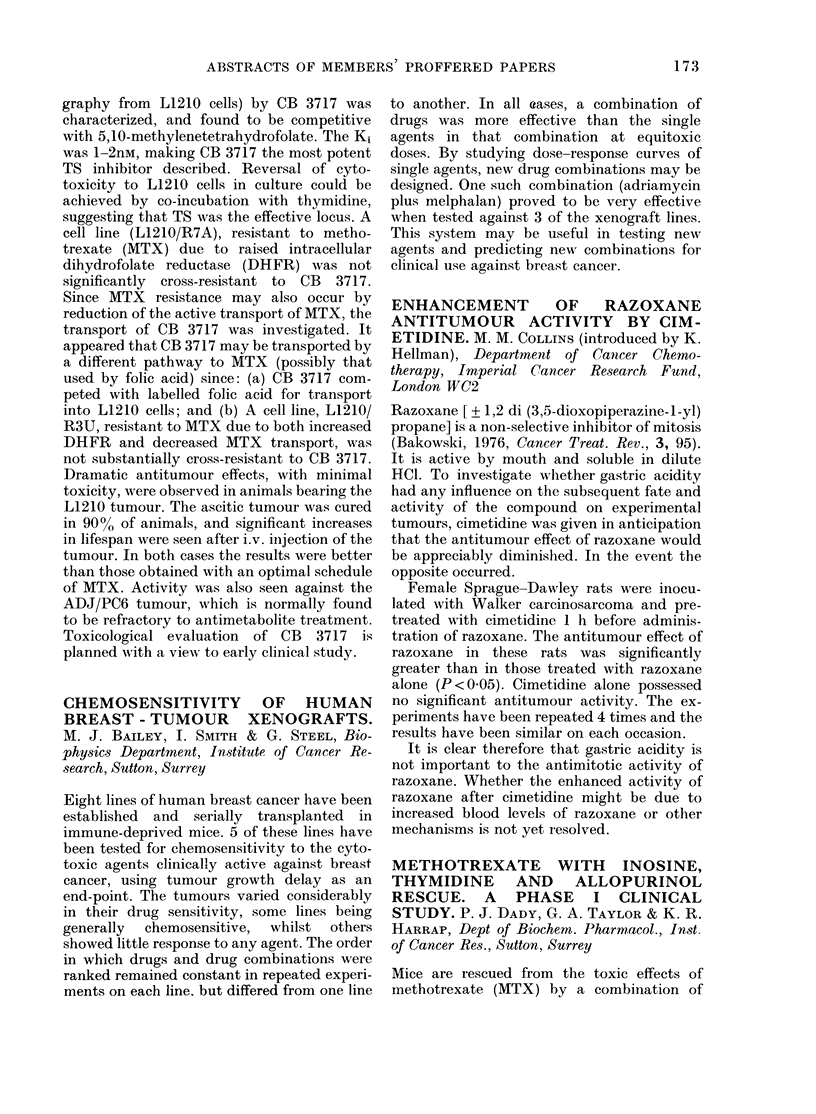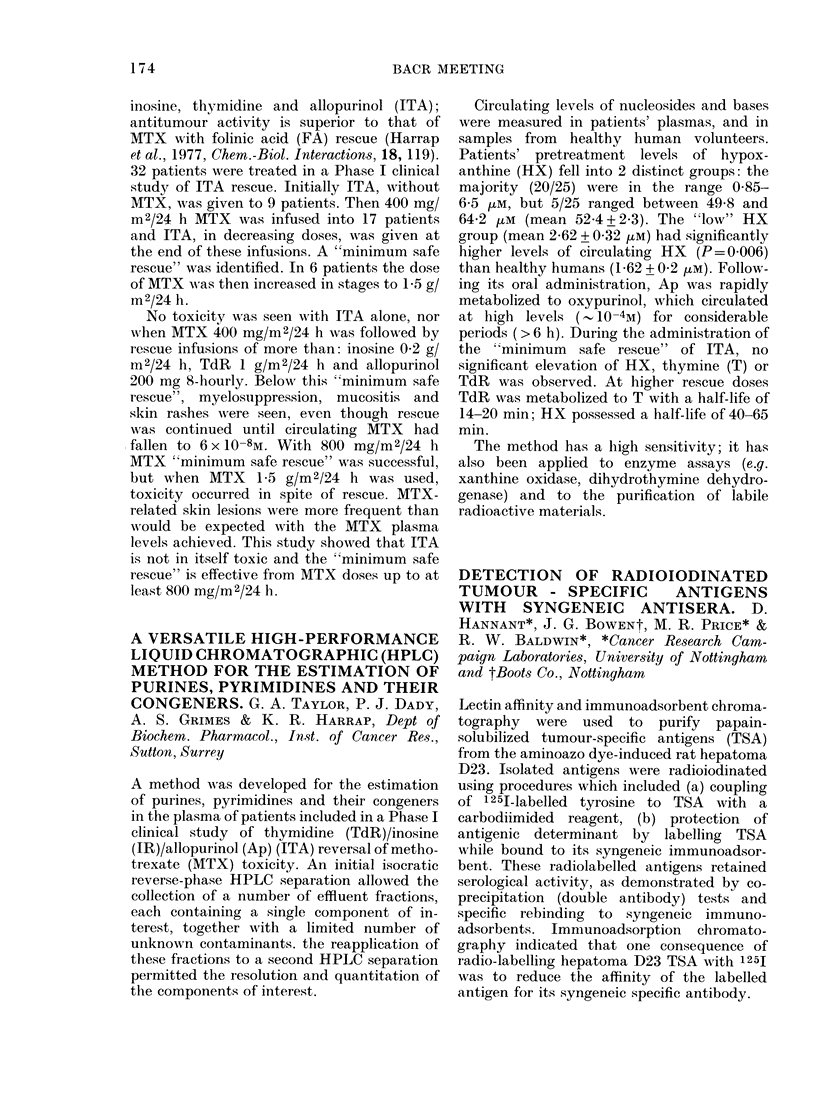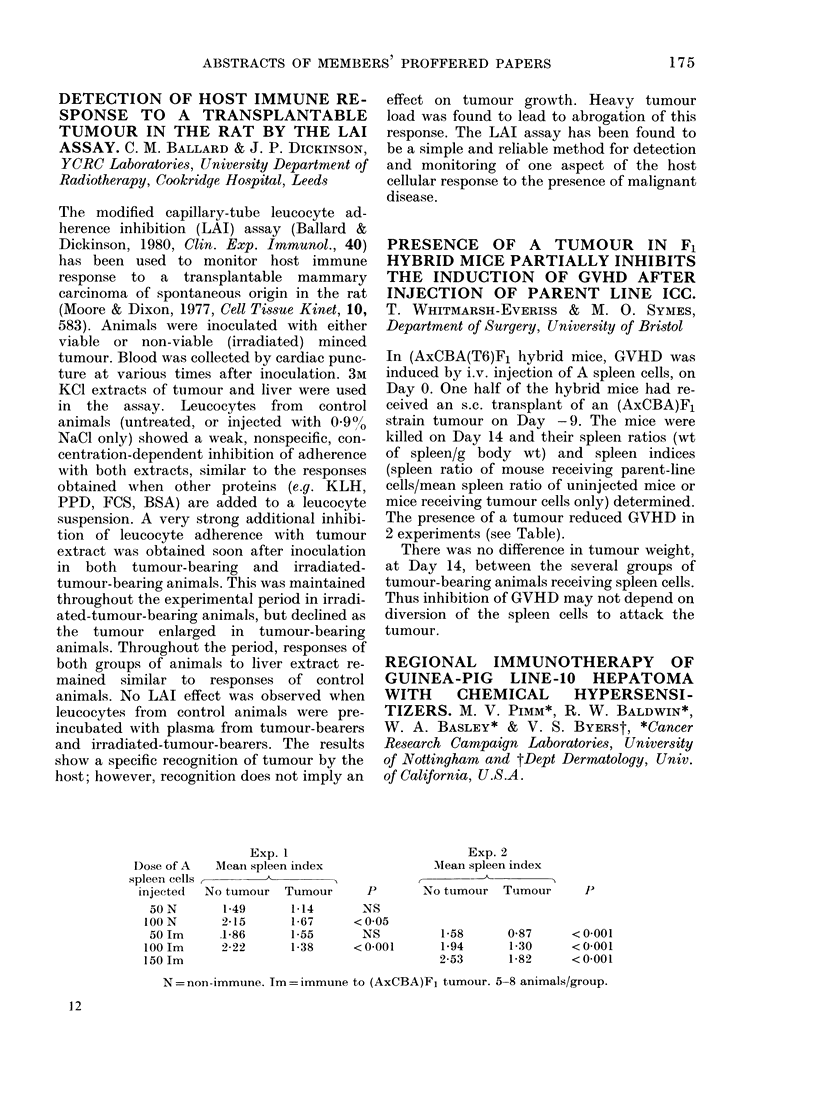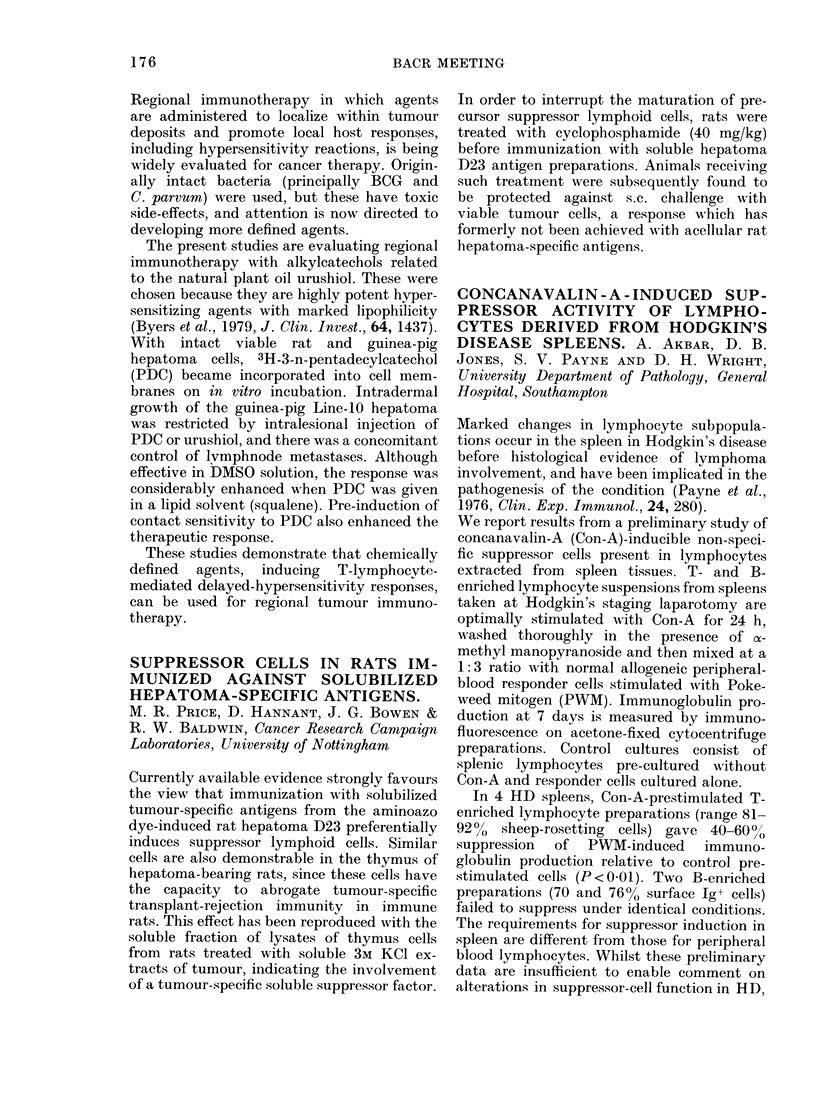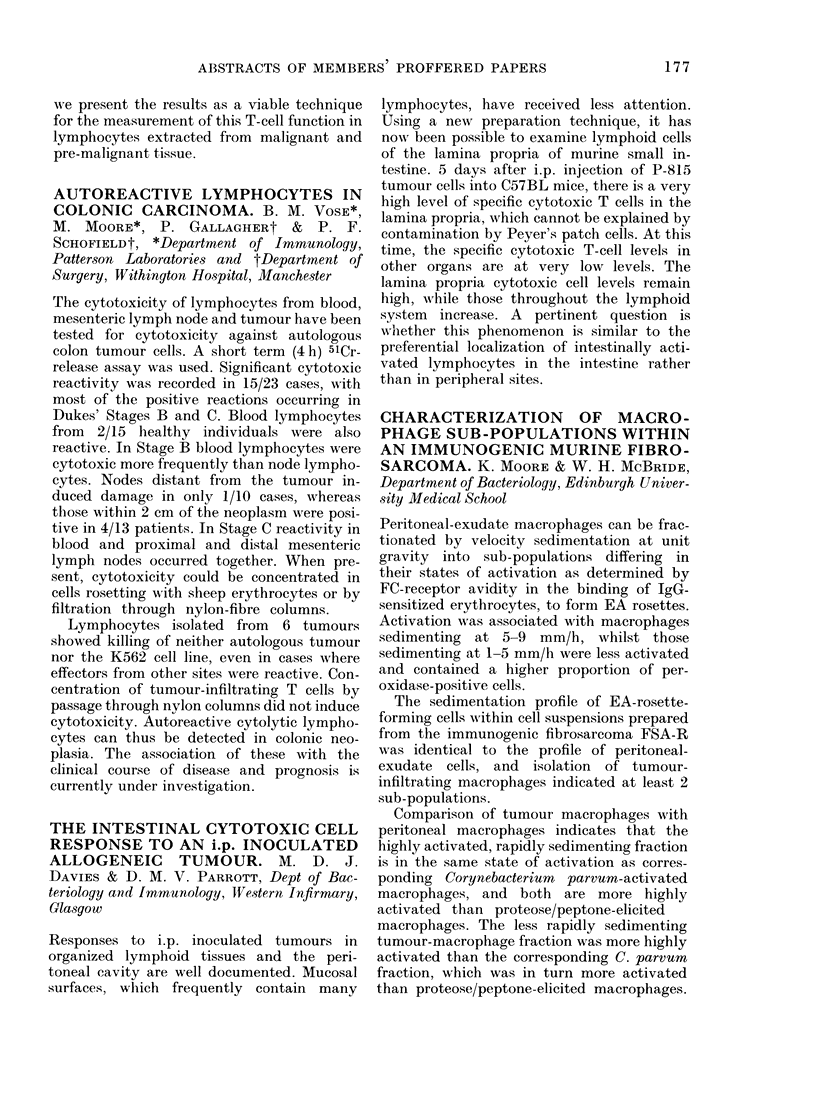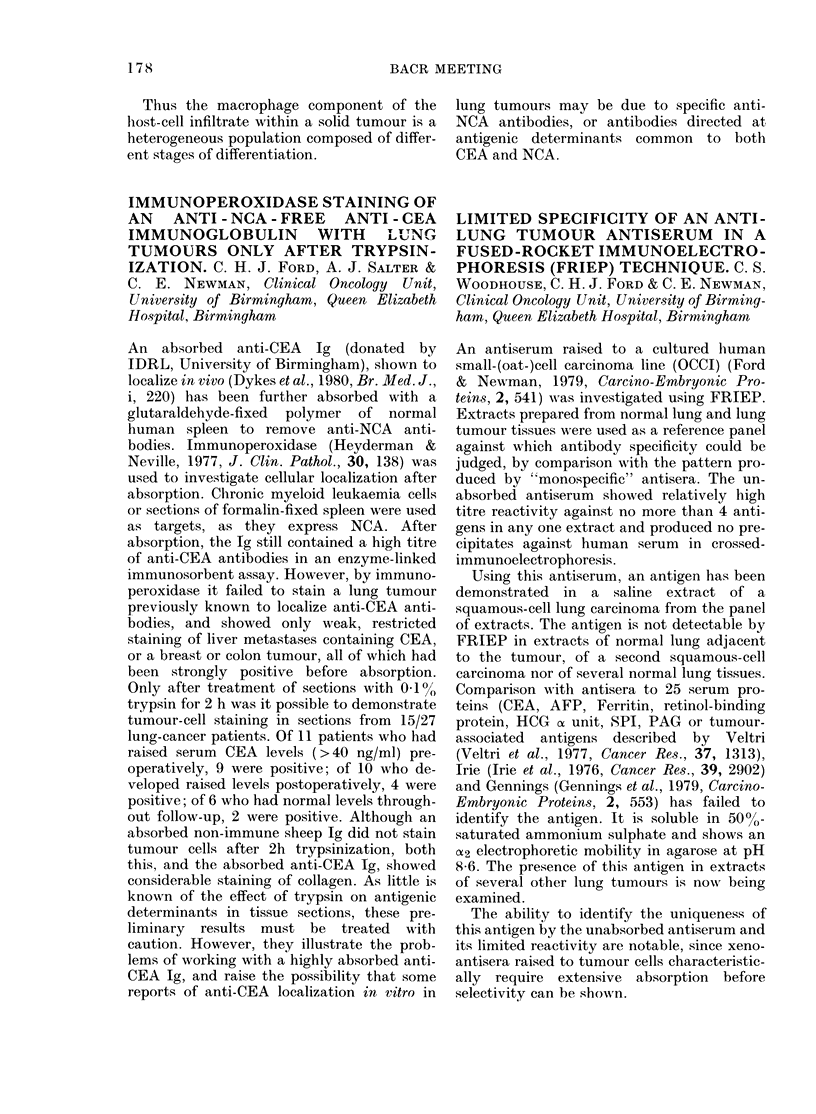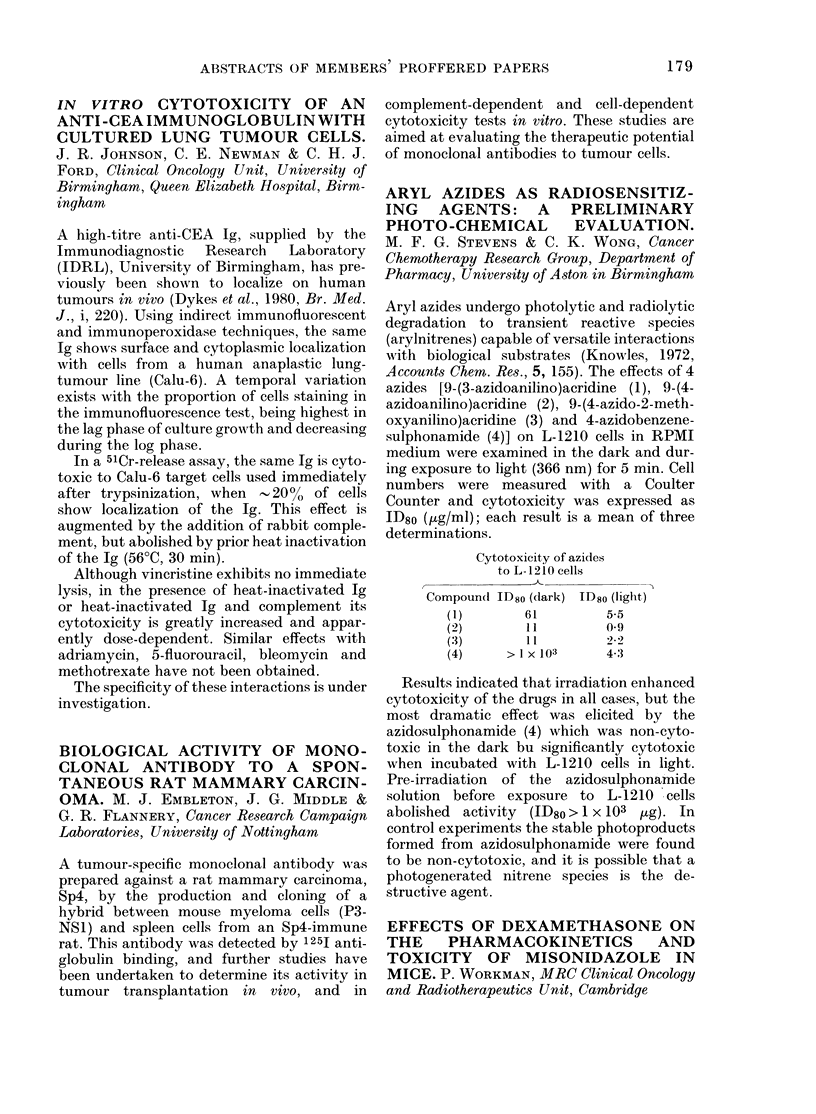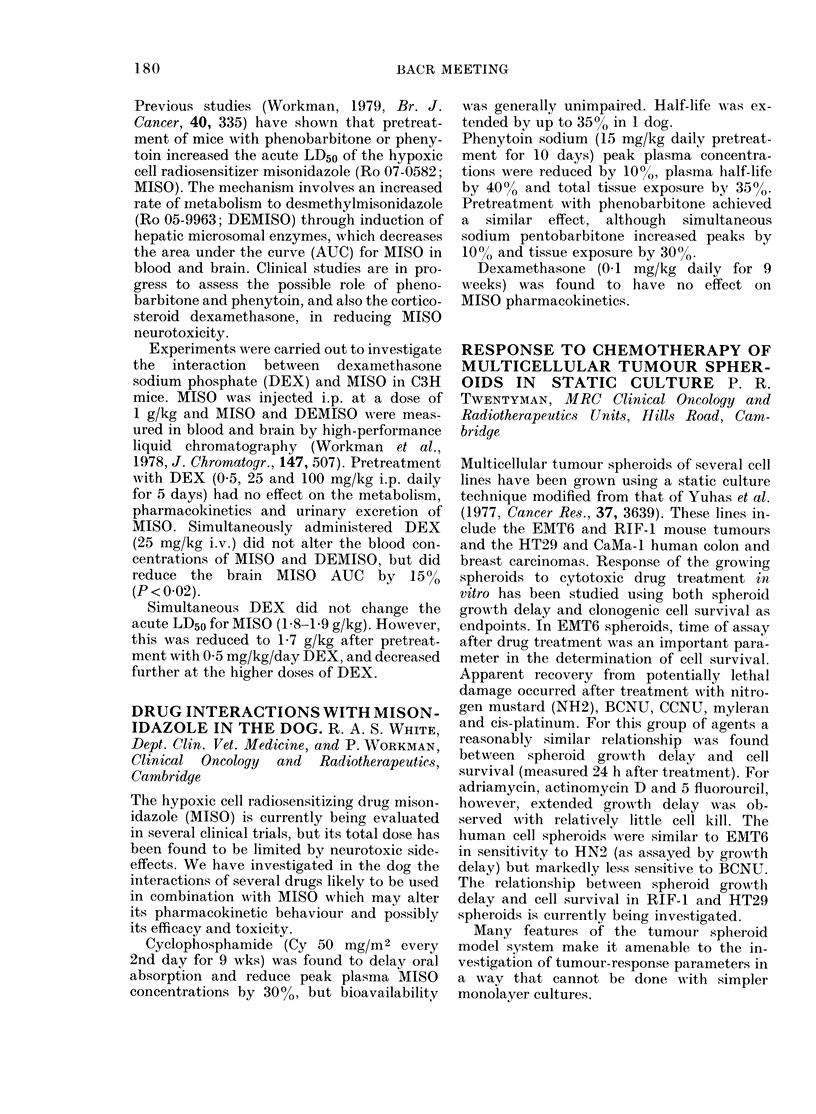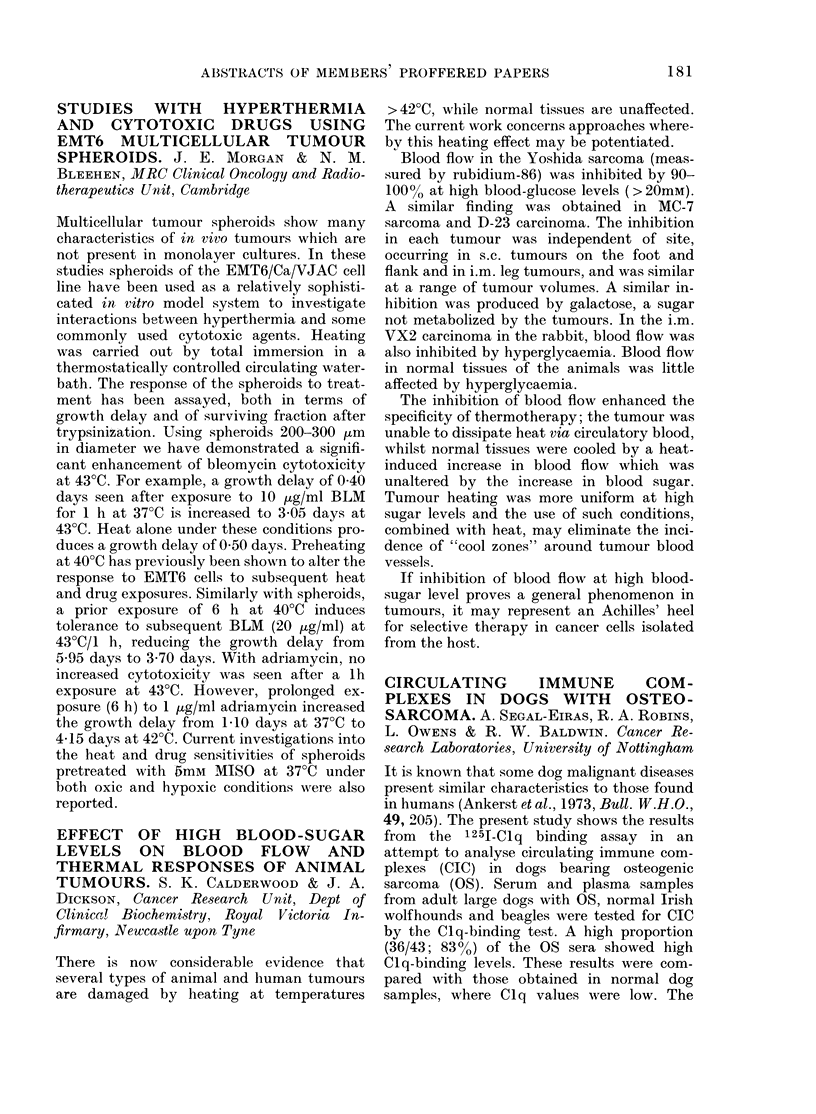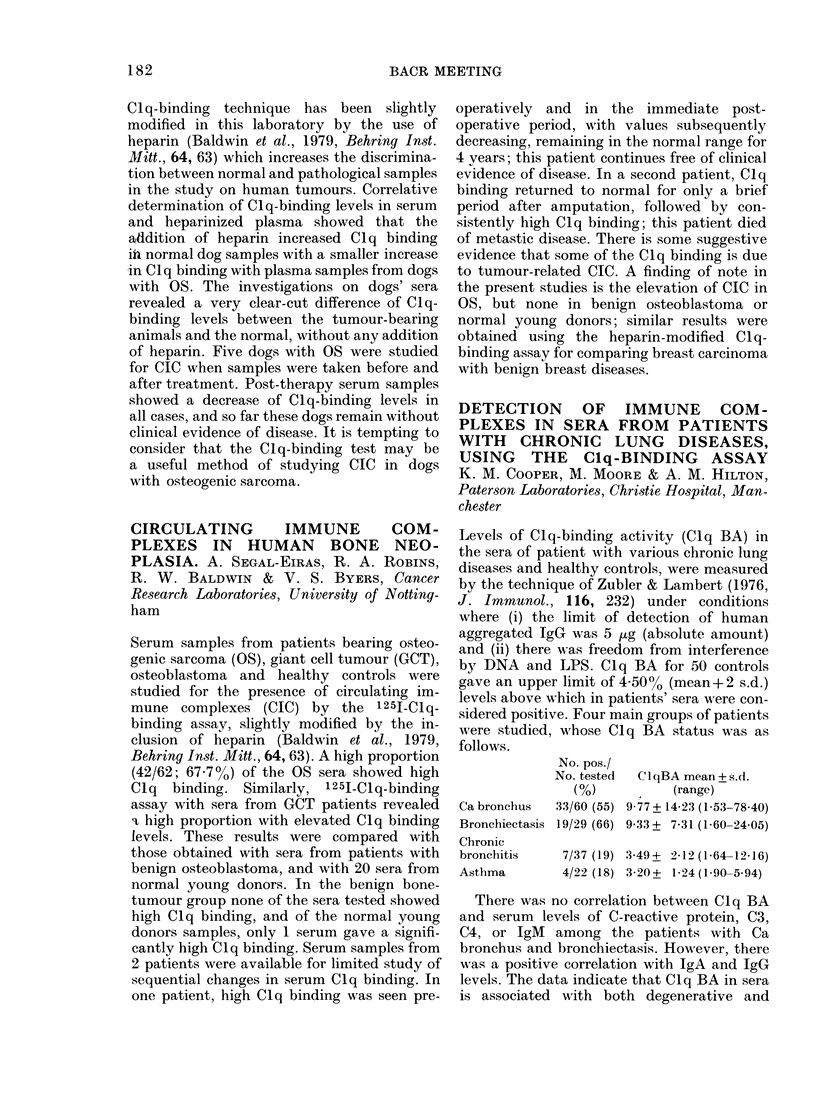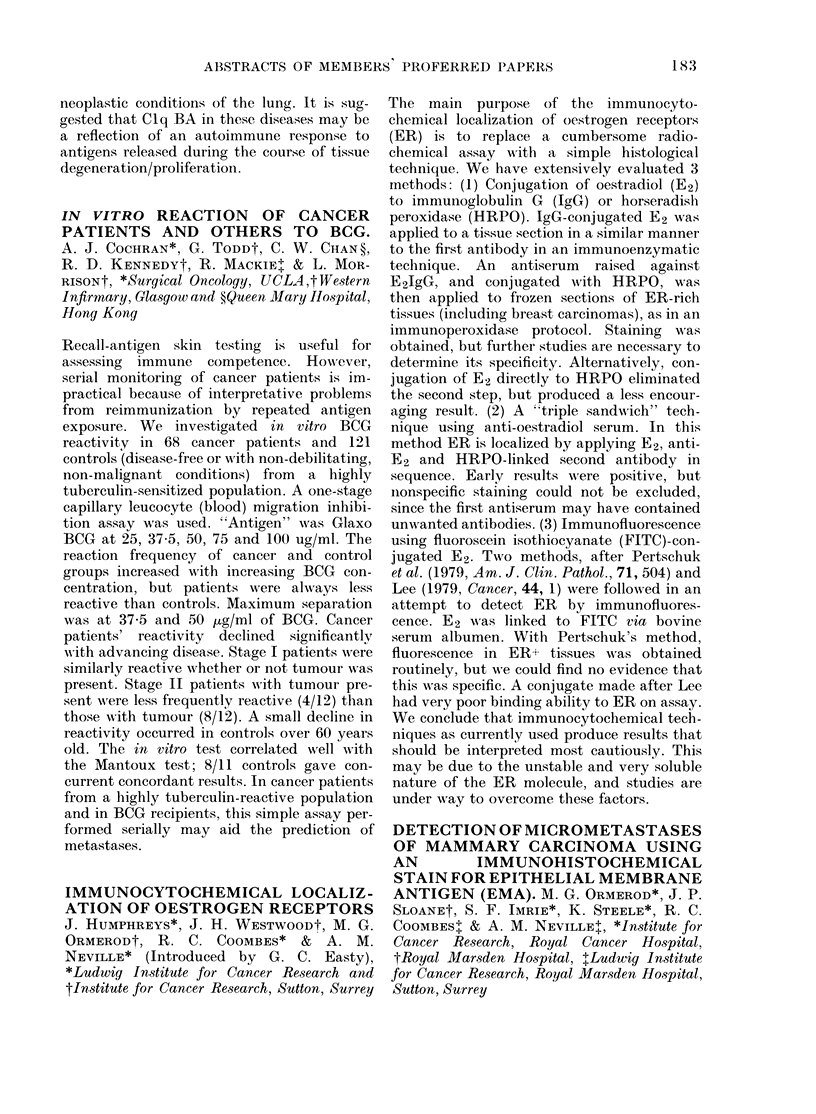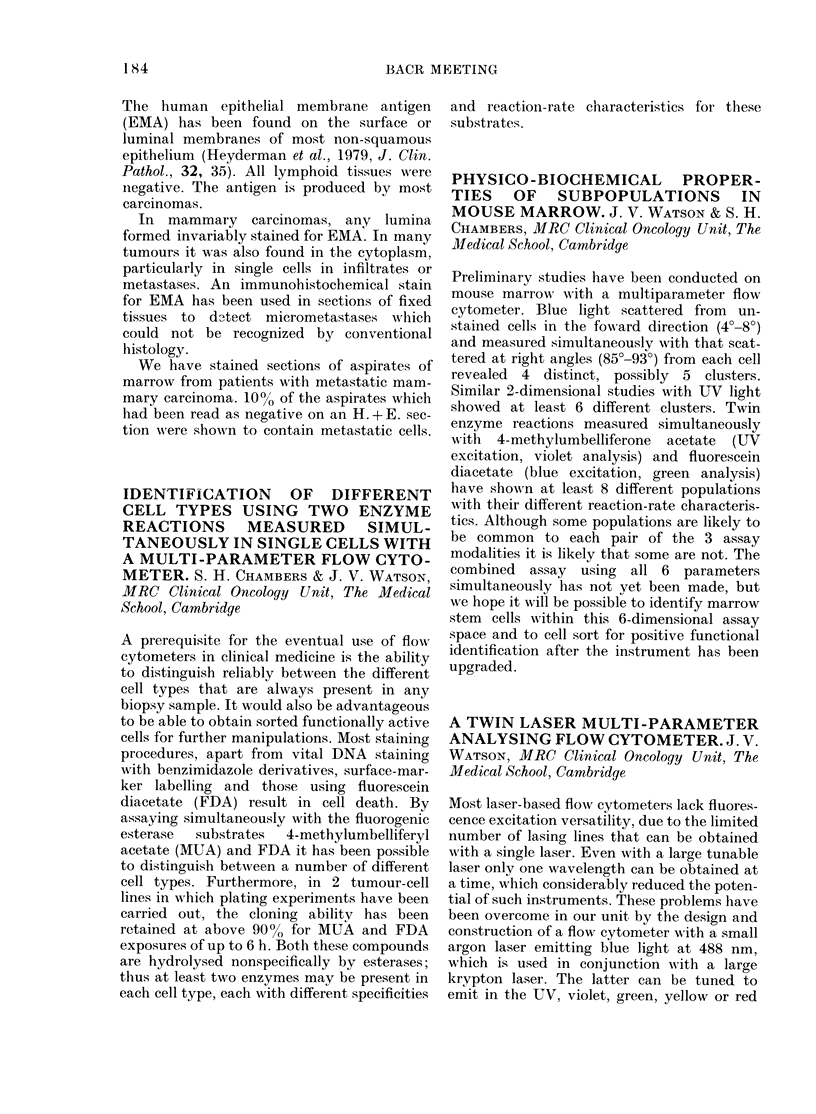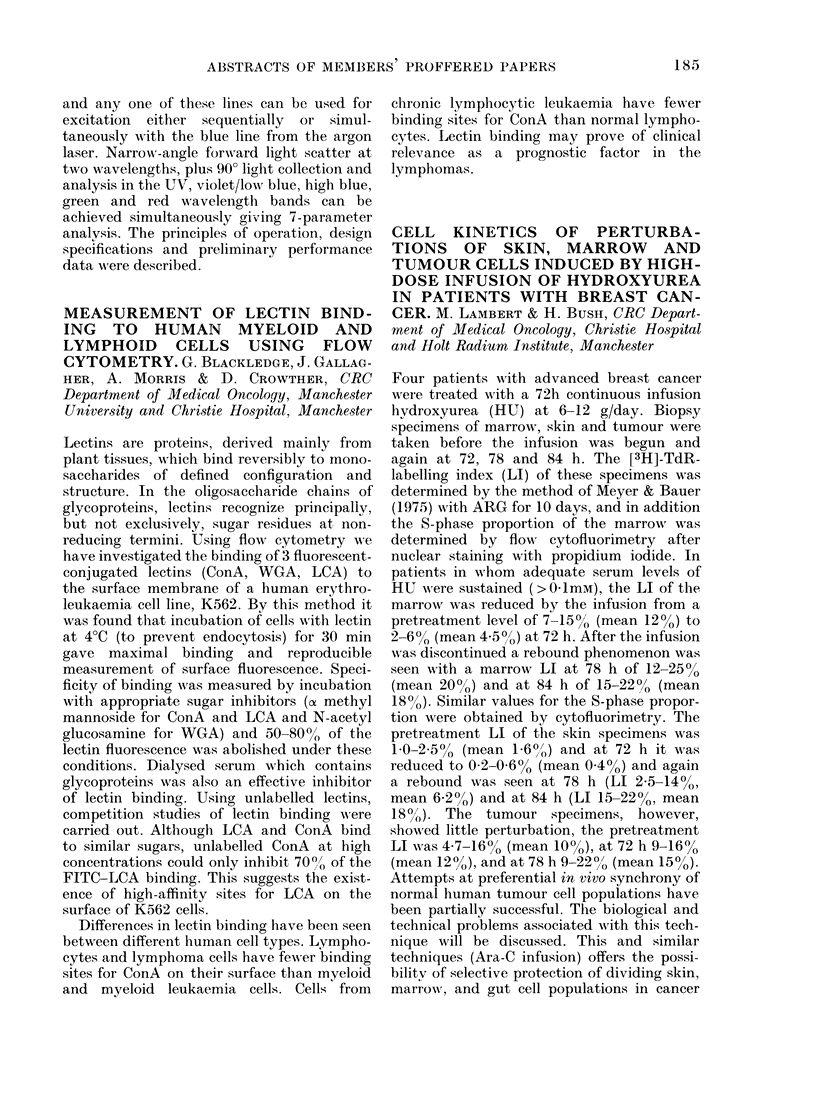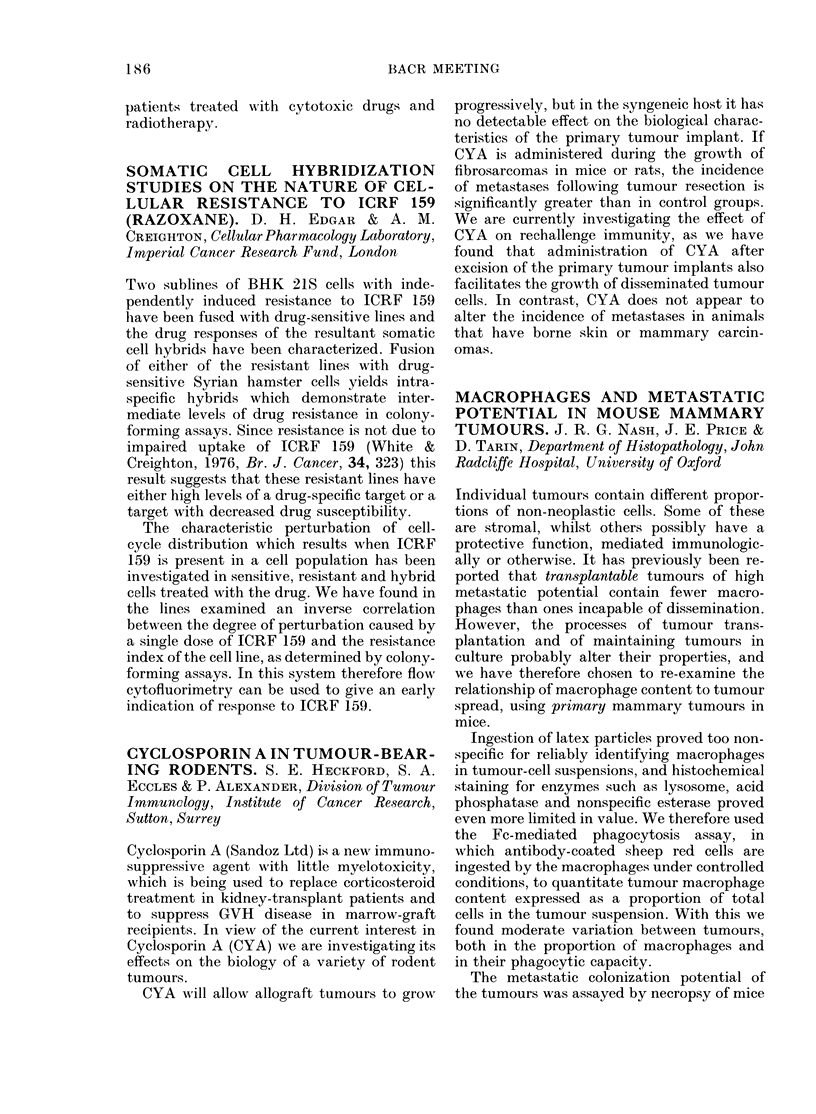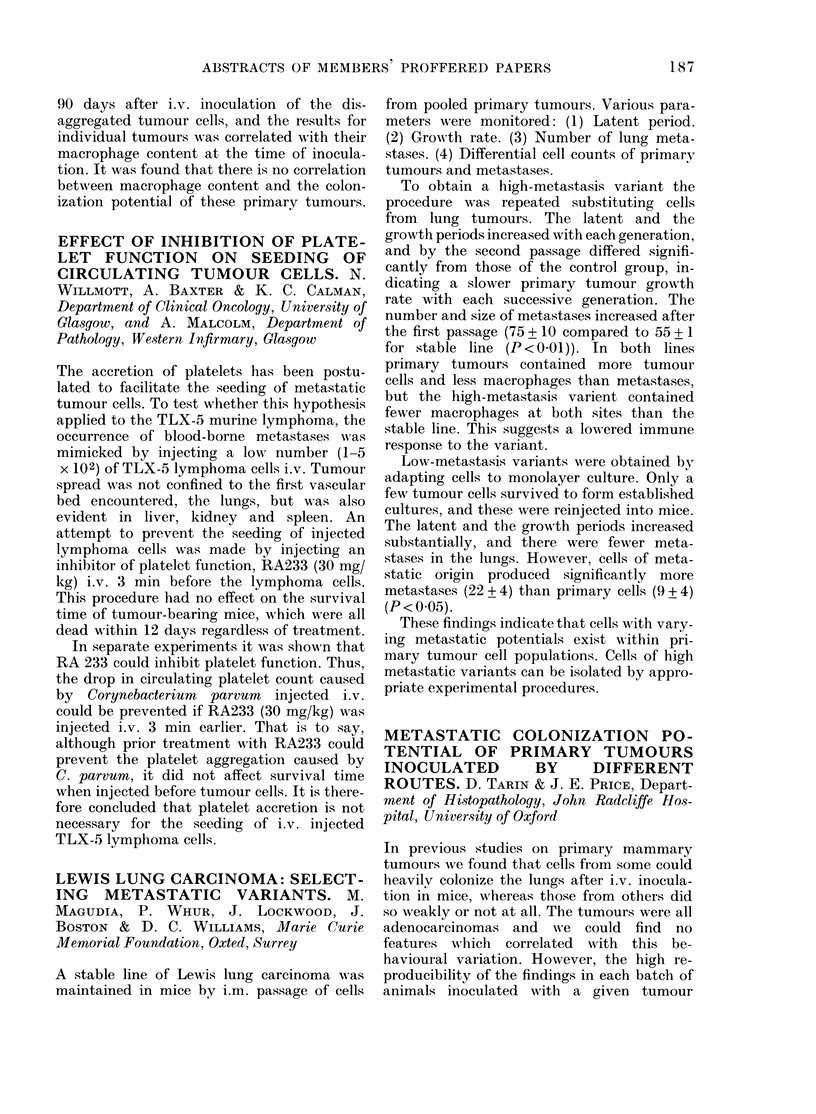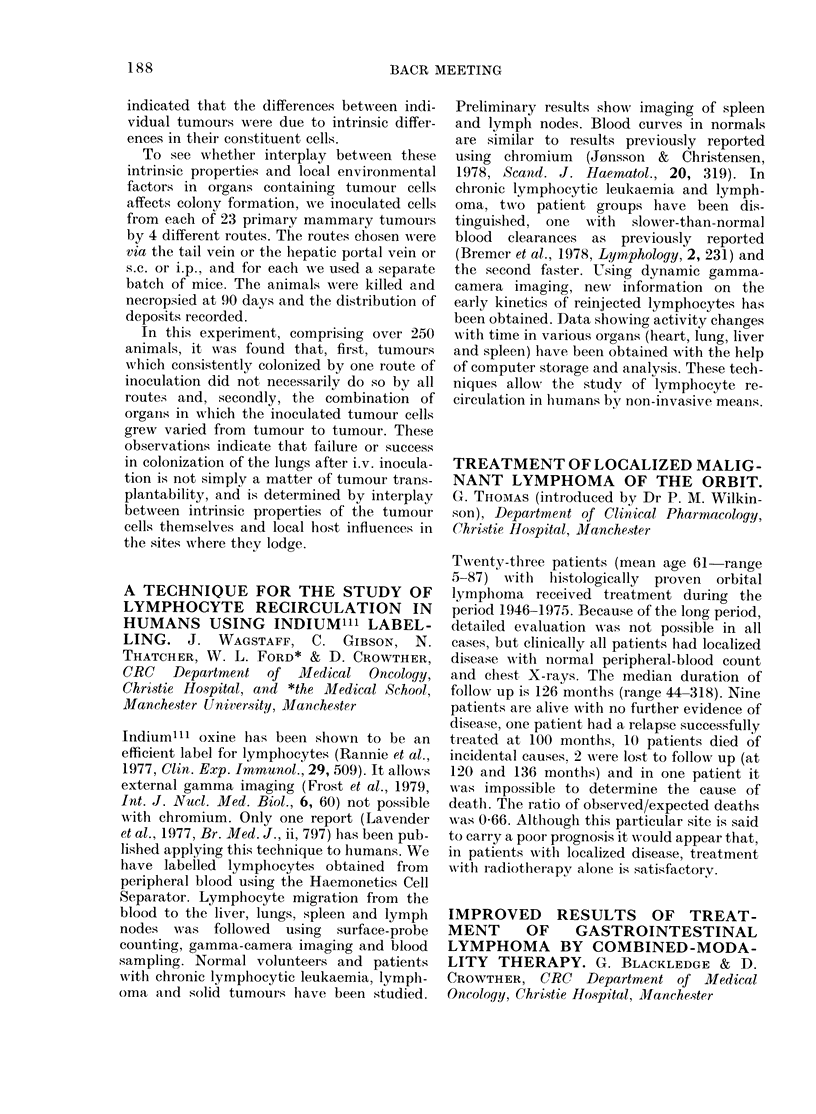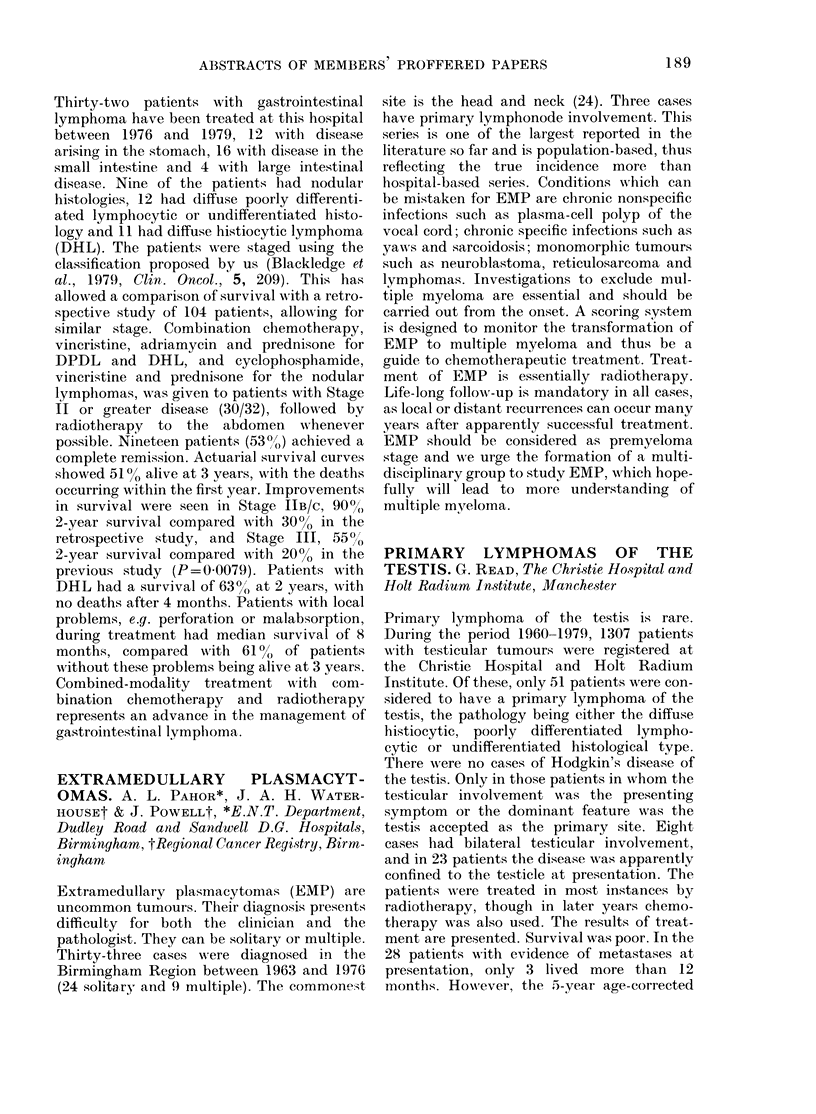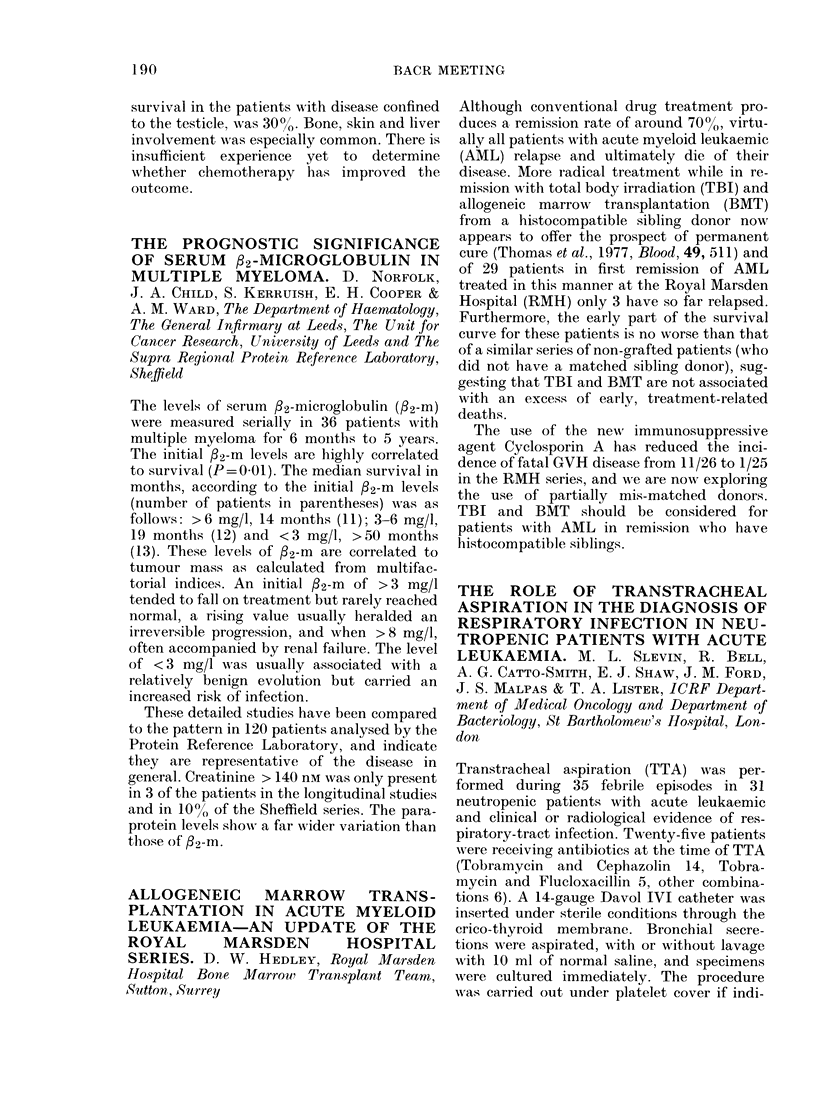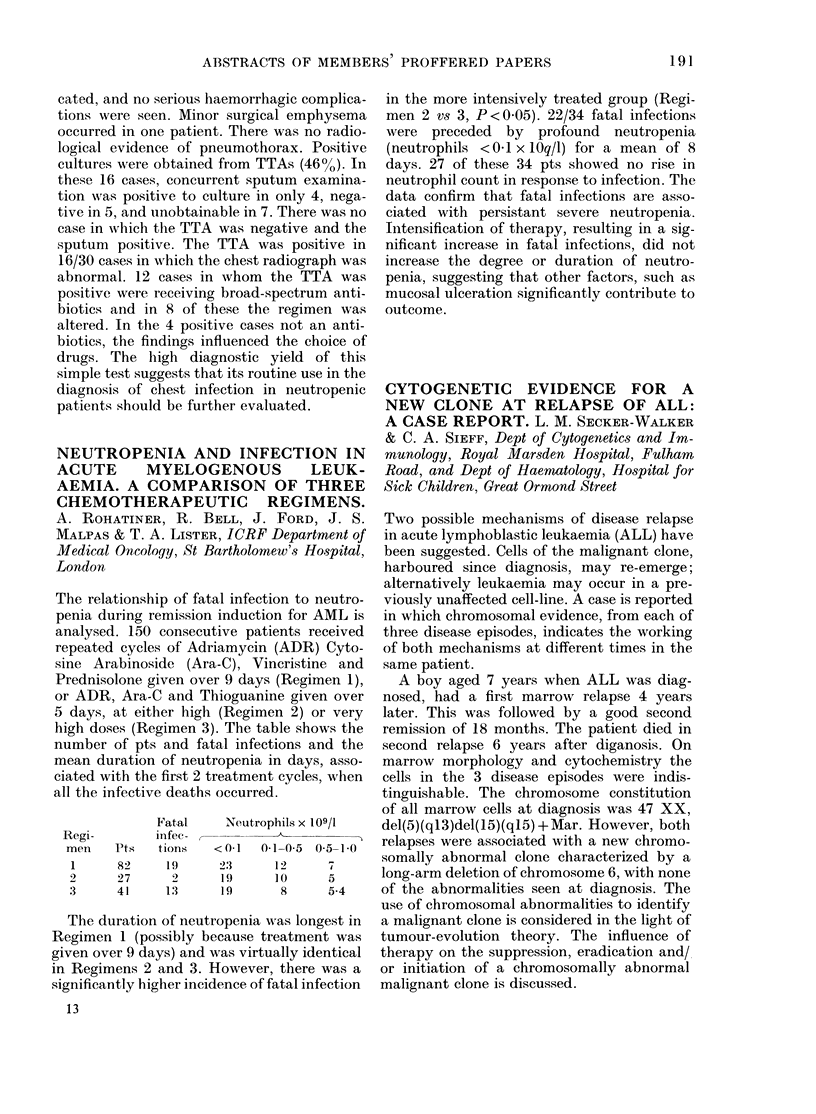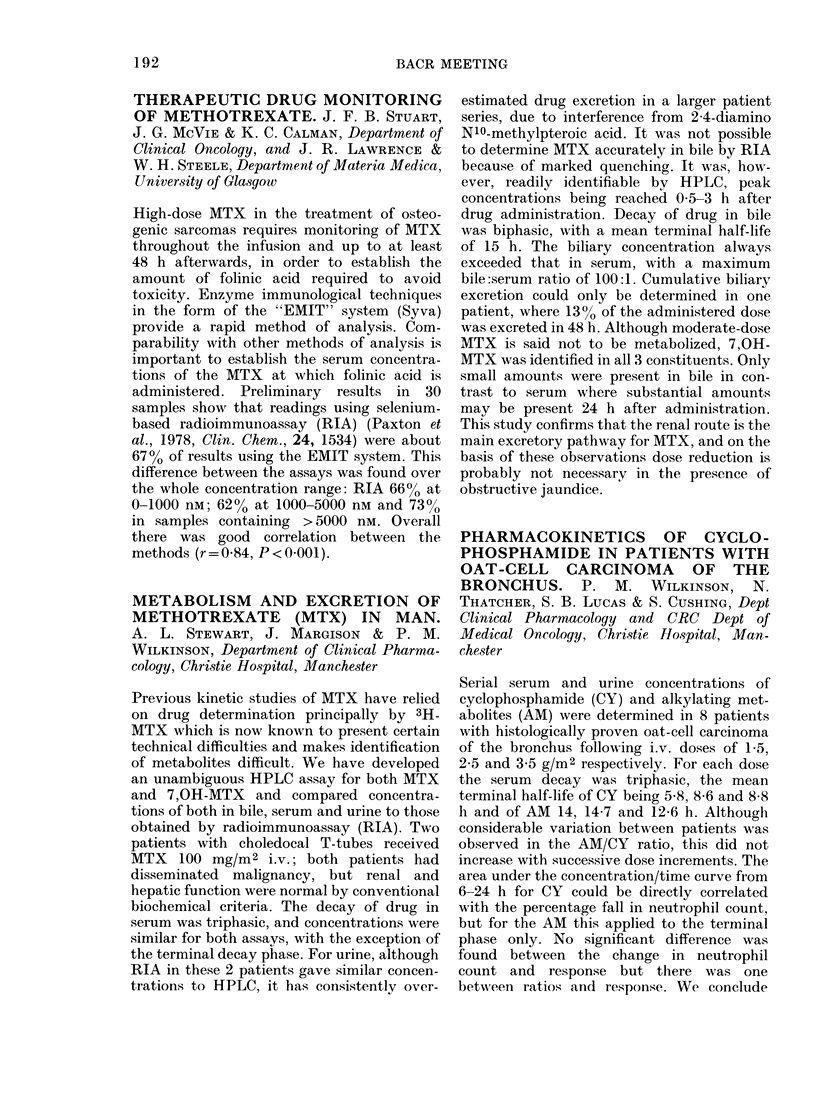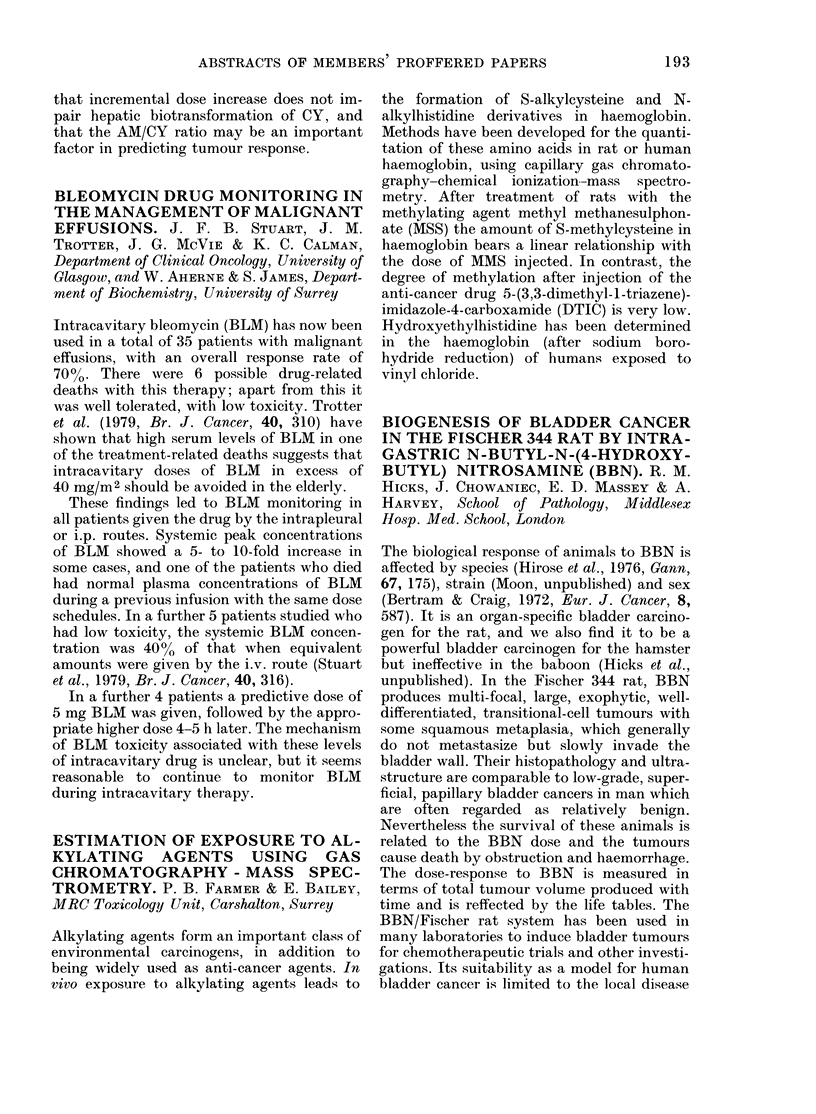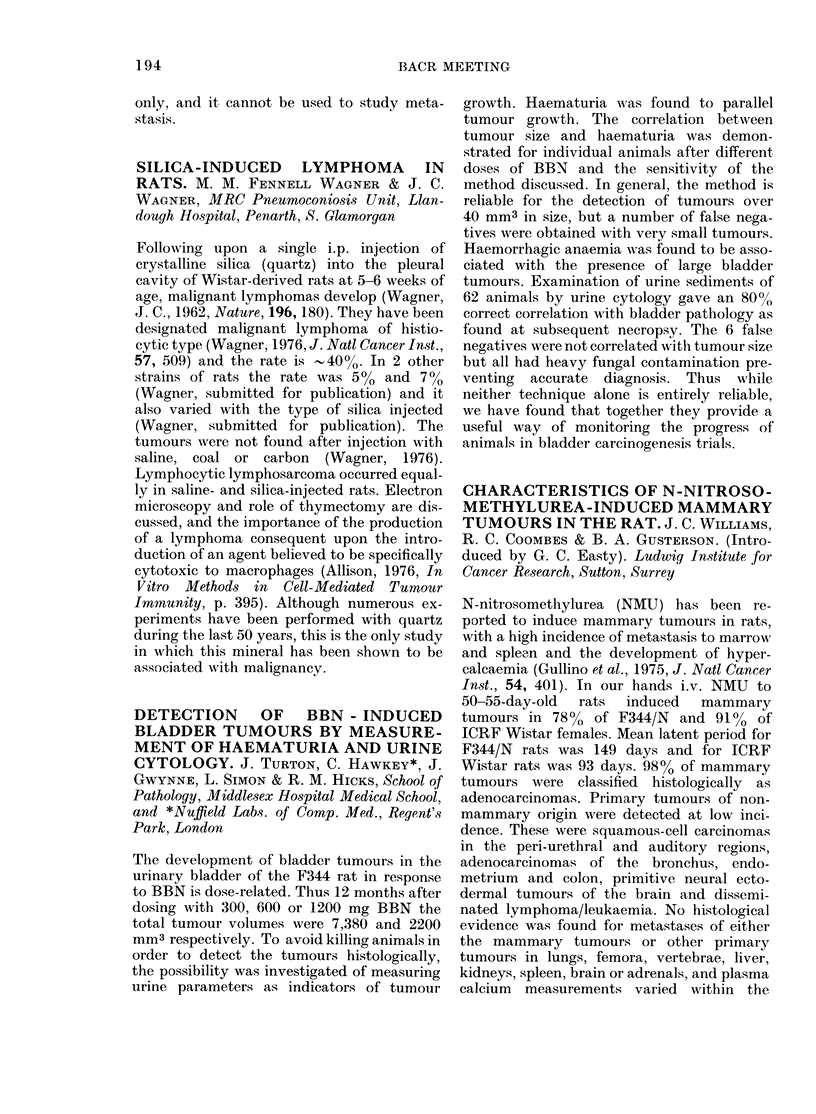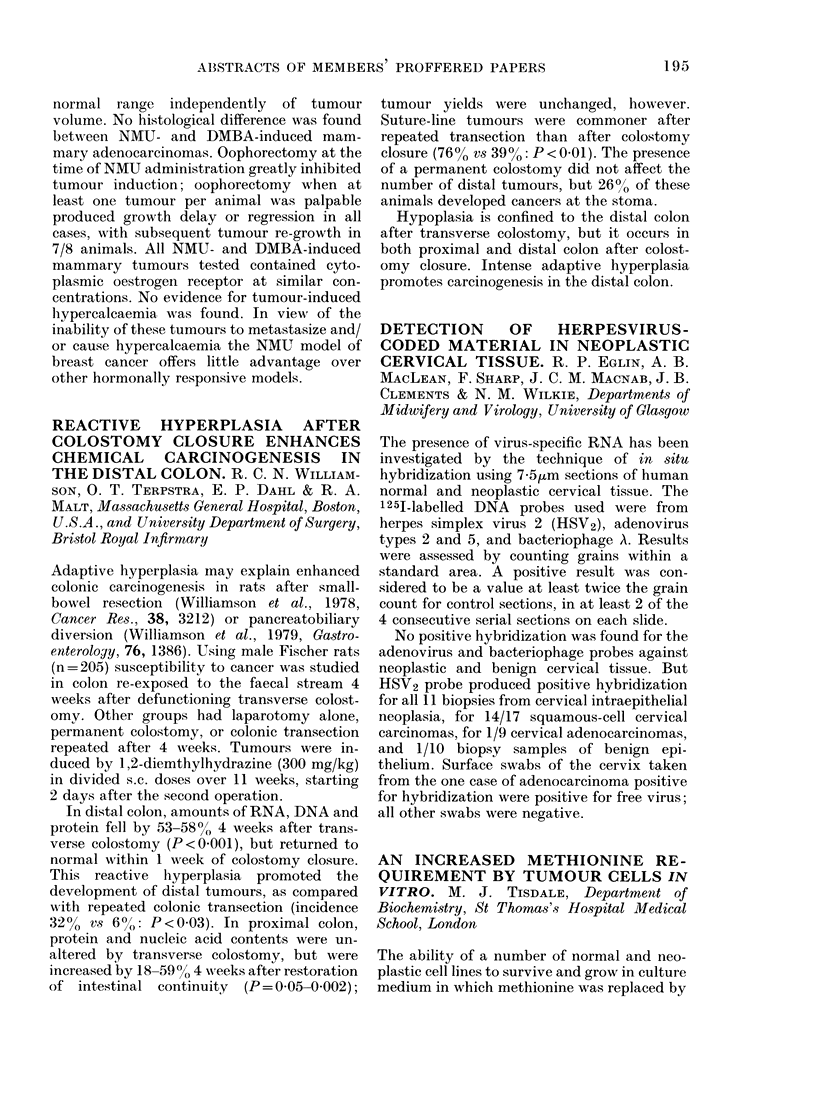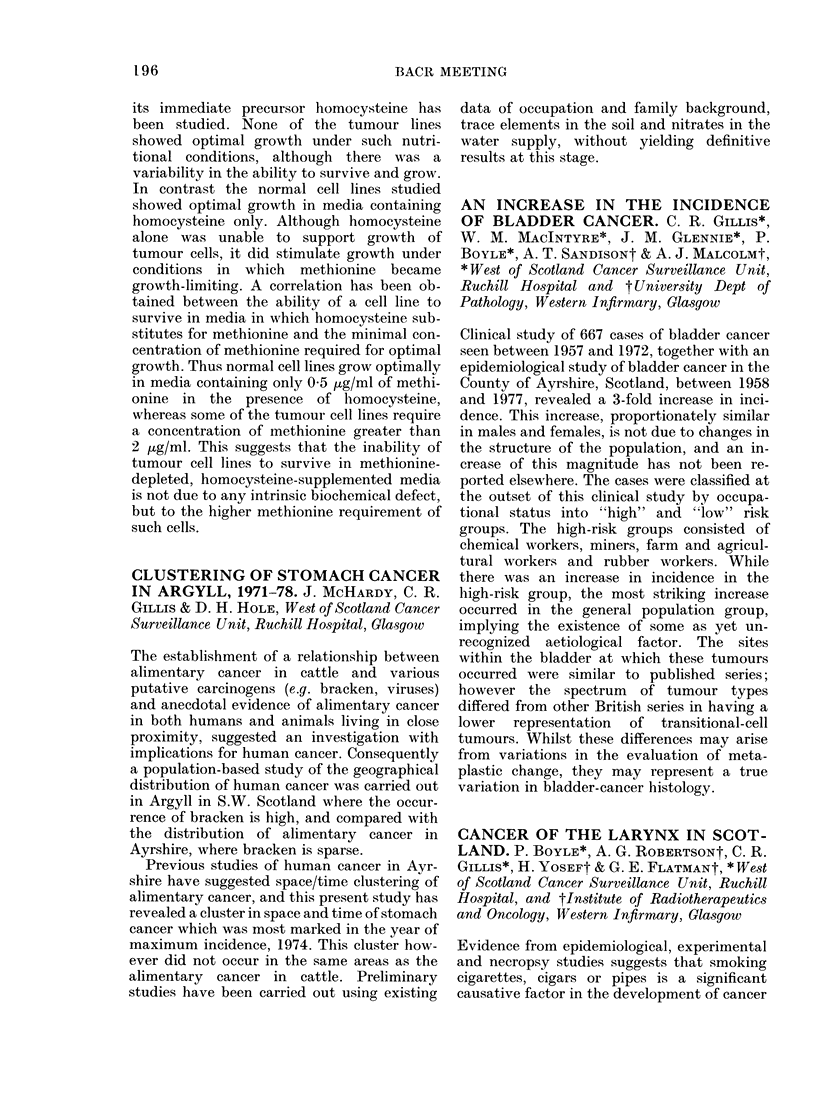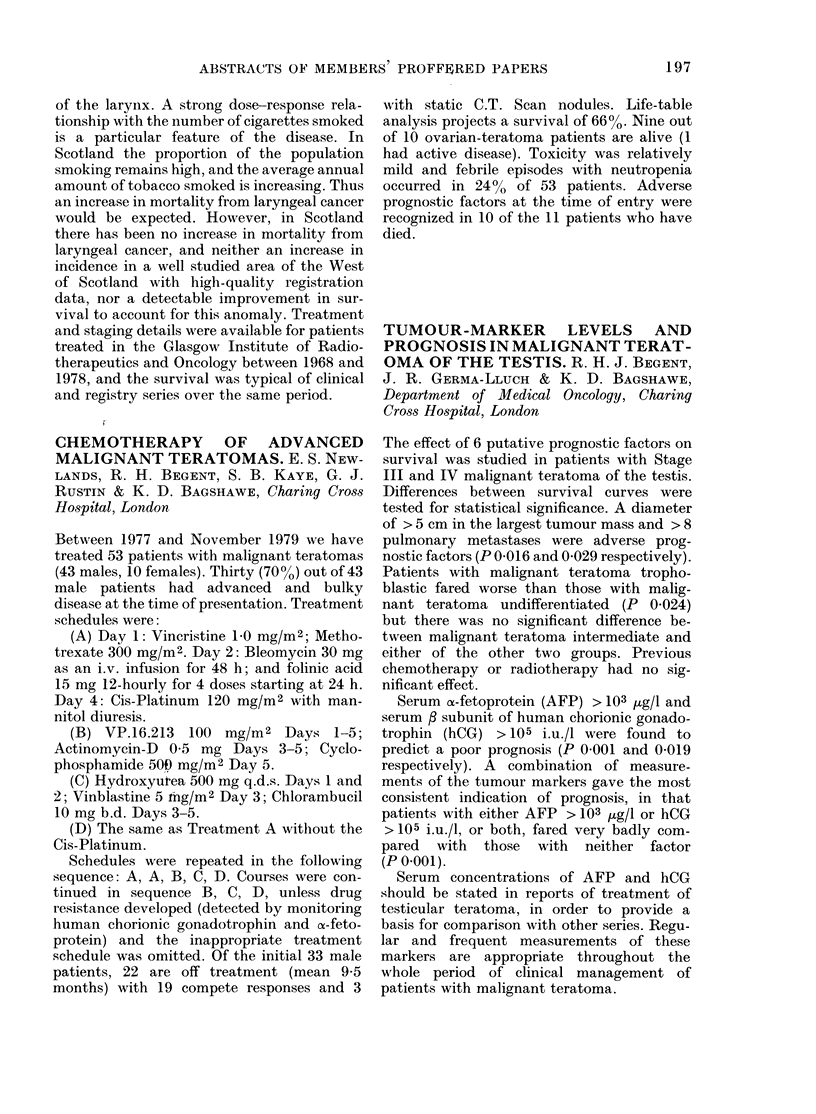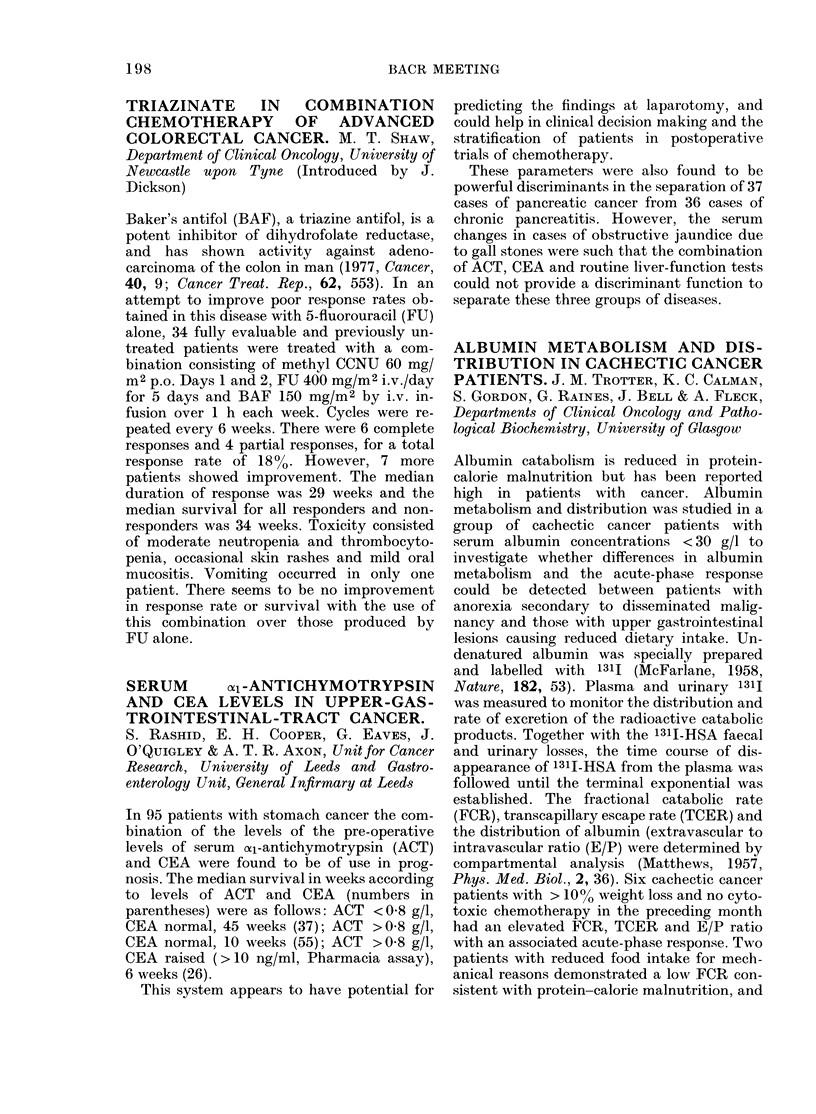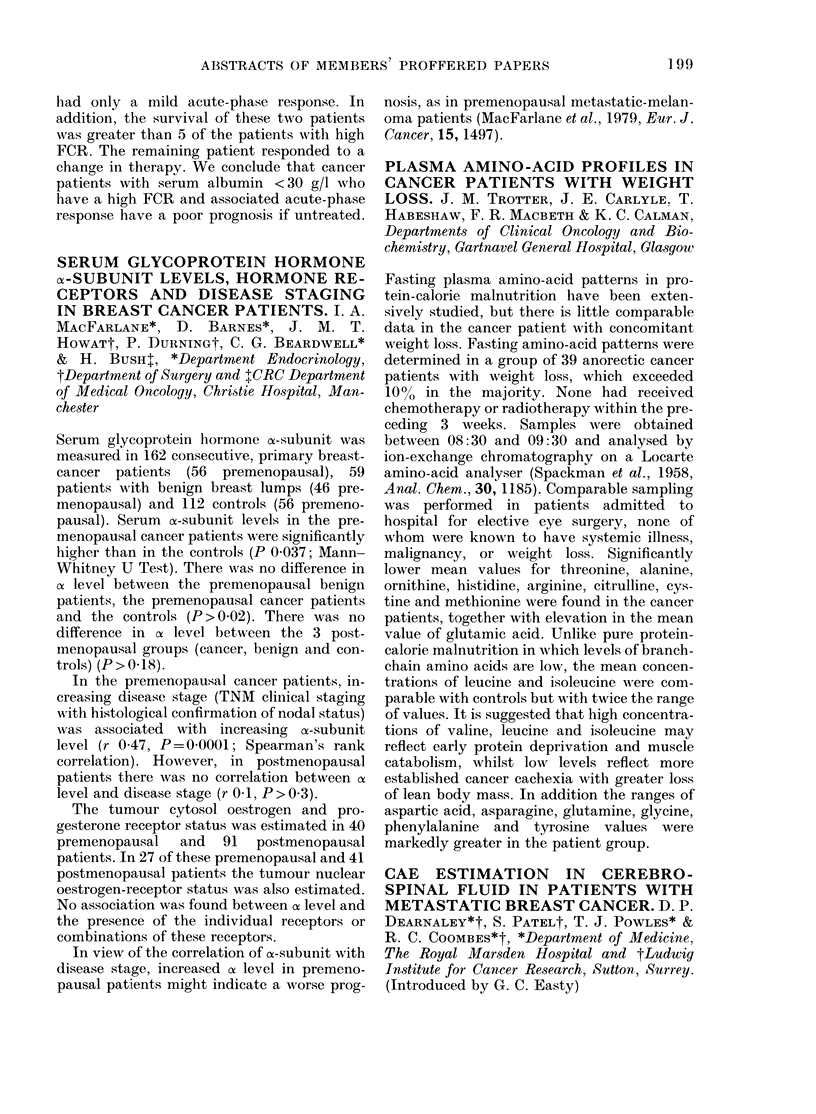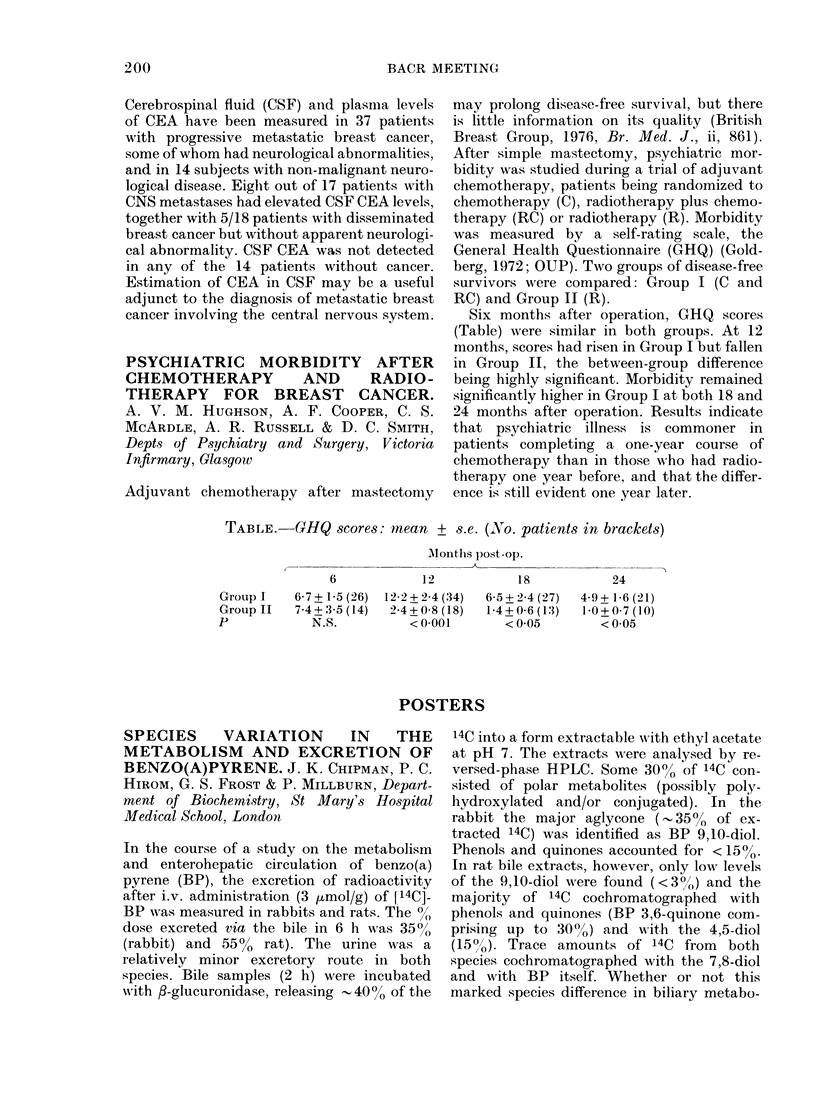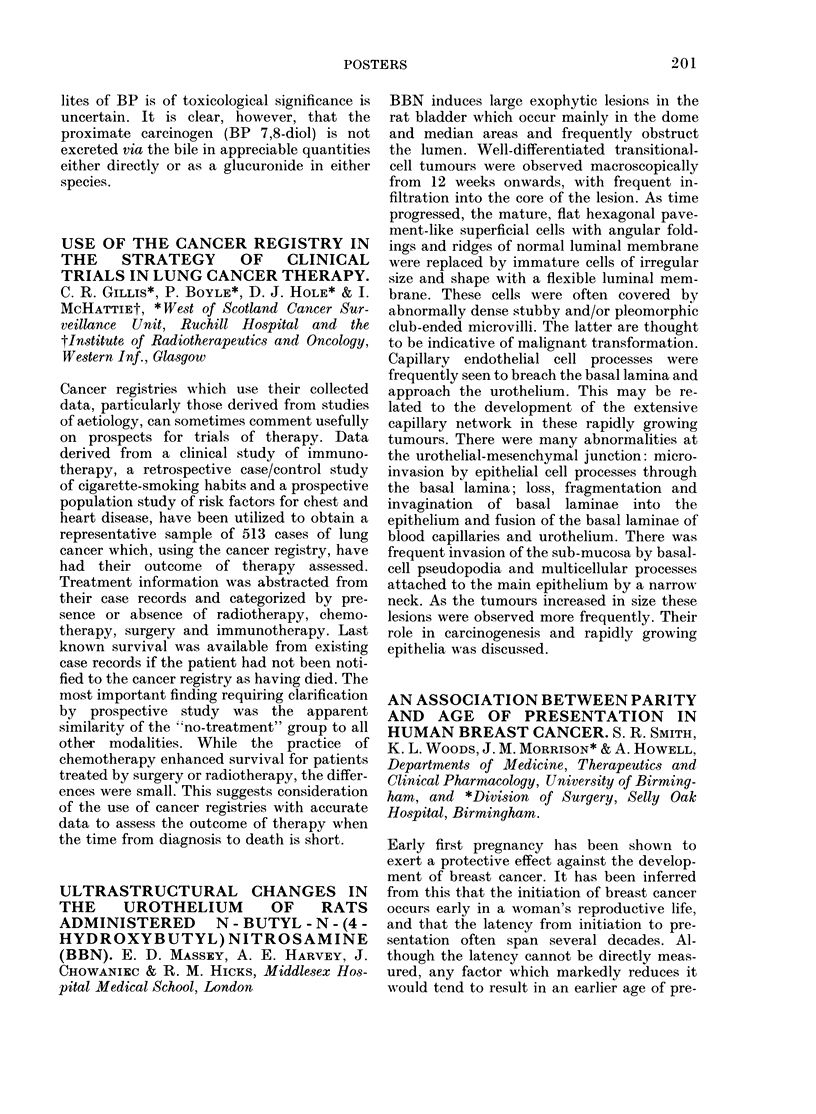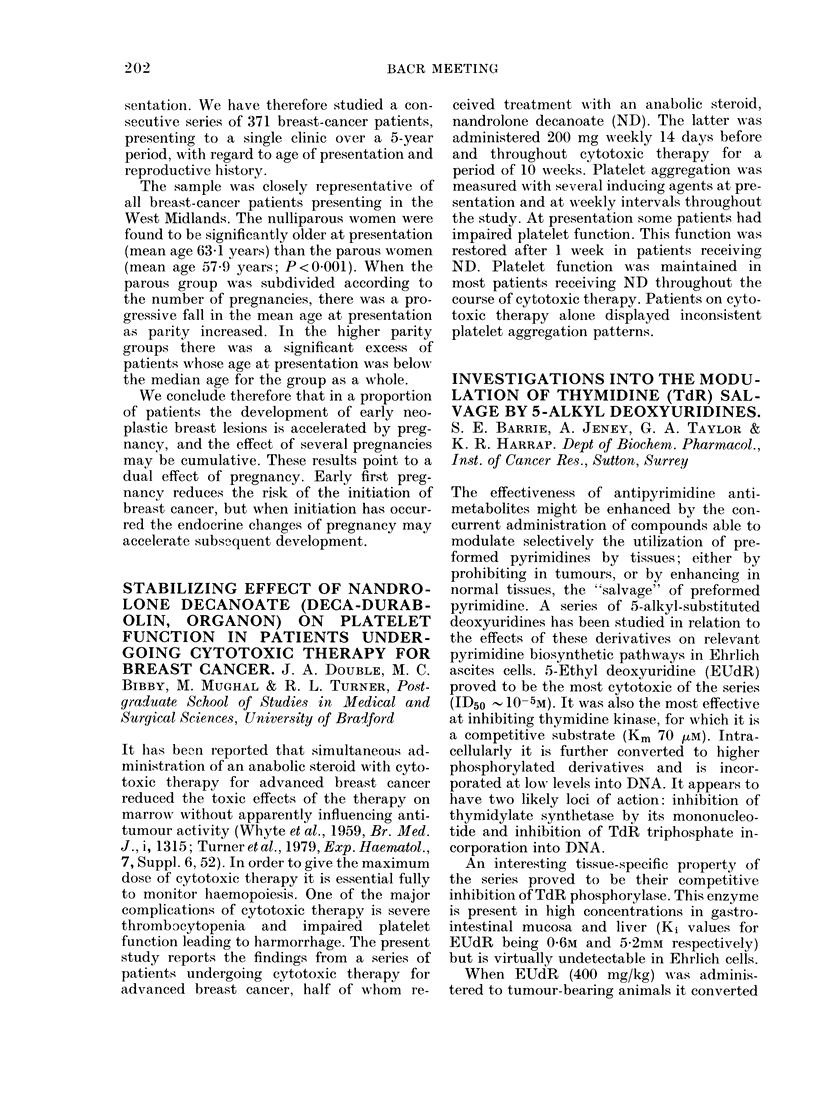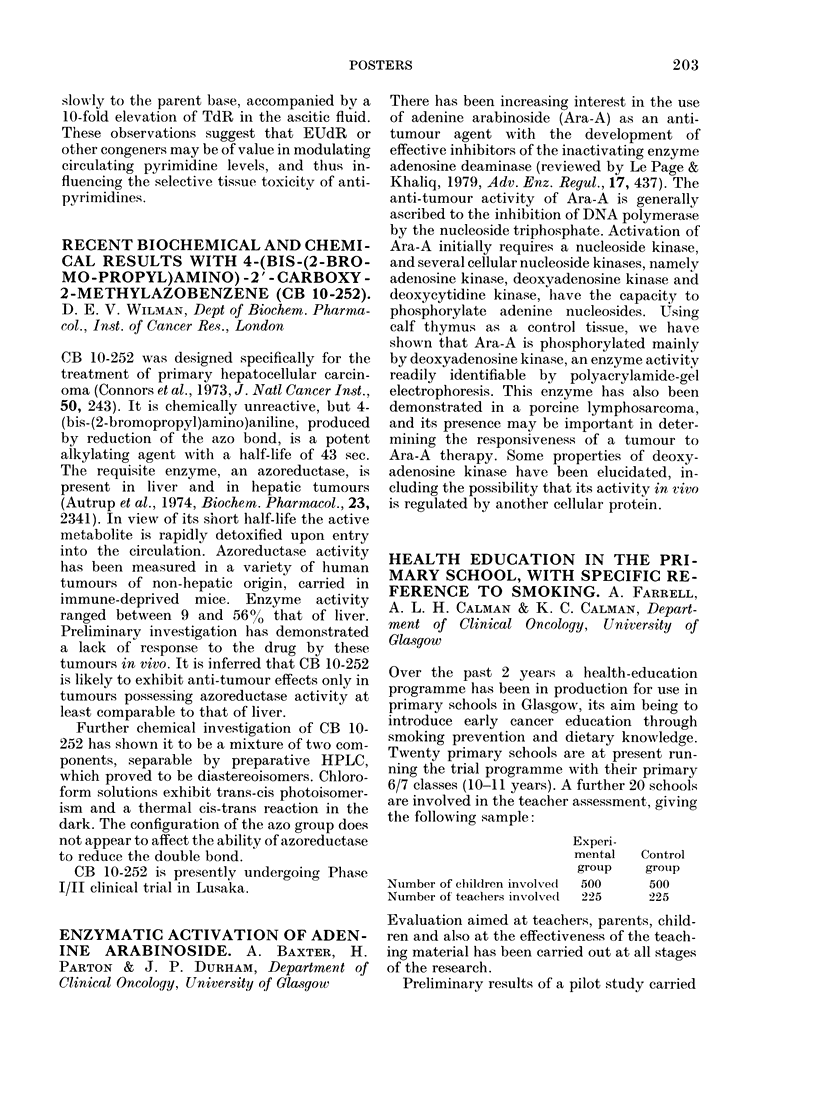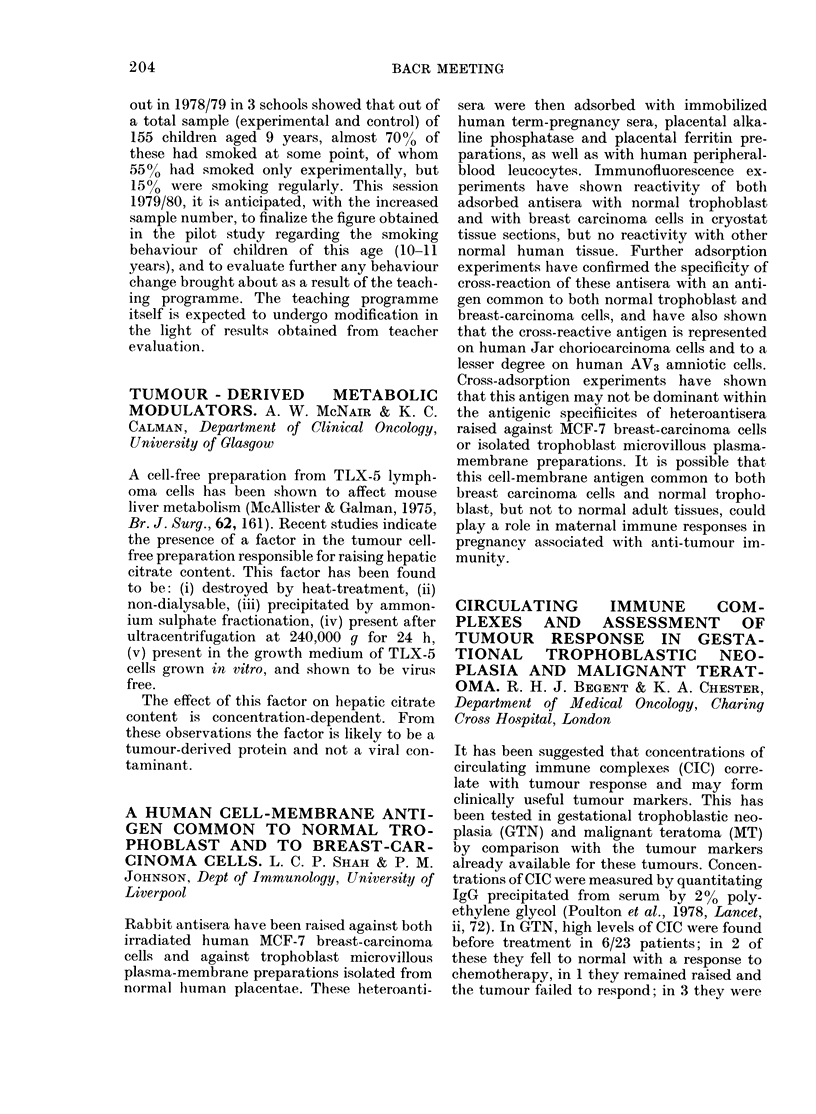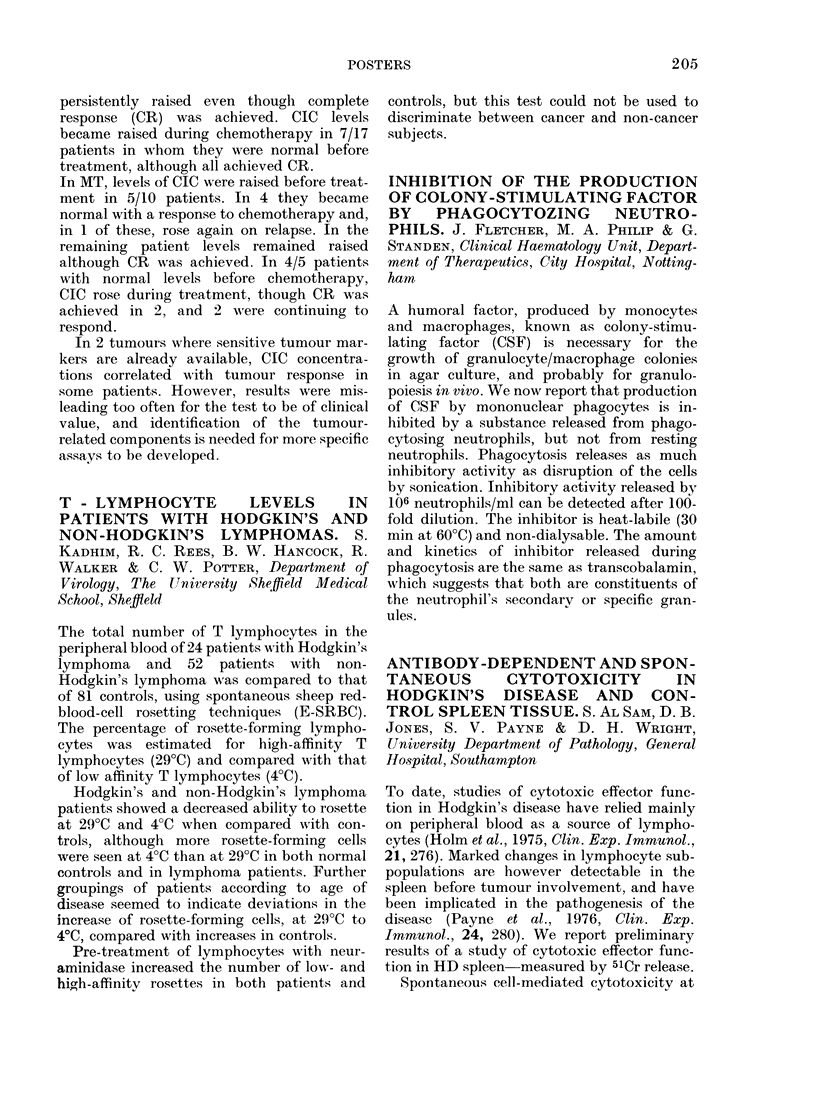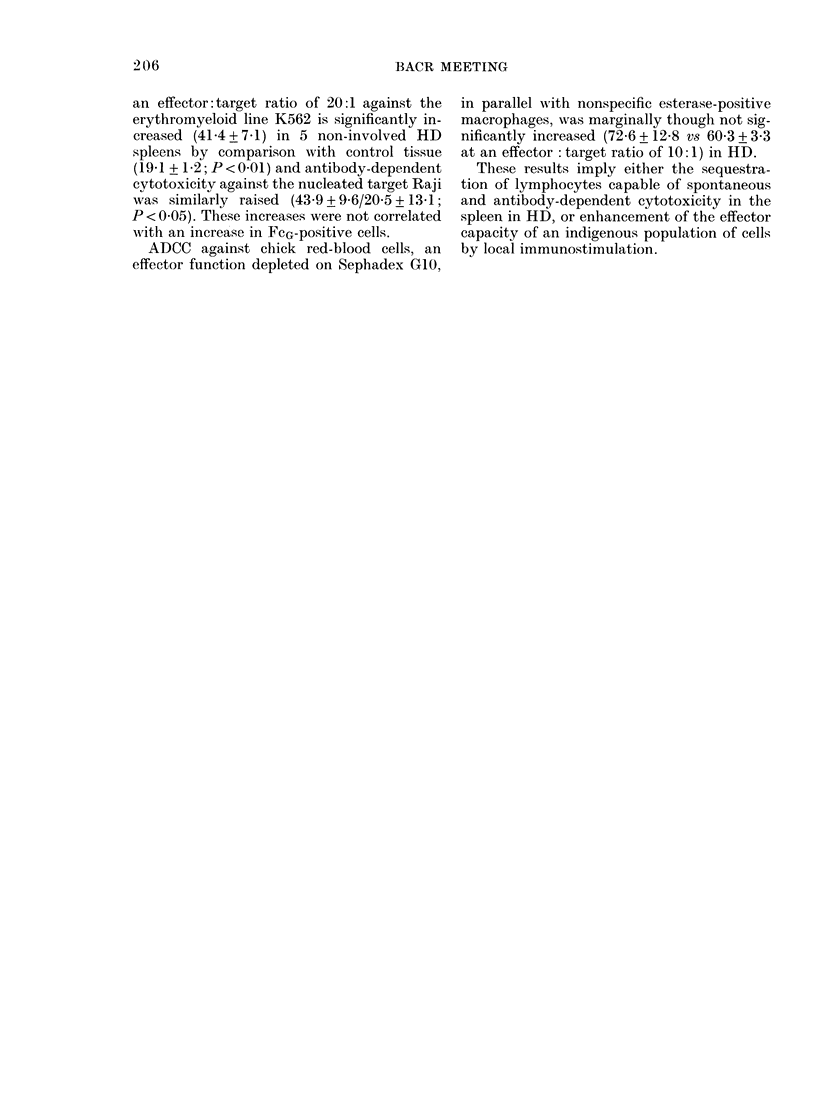# B.A.C.R. Abstracts of member proffered papers

**Published:** 1980-07

**Authors:** 


*Br. J. Cancer* (1980) **41**, 168

### BRITISH ASSOCIATION FOR CANCER RESEARCH
### 21st ANNUAL GENERAL MEETING

*Held at the University of Southampton 31 March–2 April 1980*

### ABSTRACTS OF MEMBERS' PROFFERRED PAPERS

**BREATH ANALYSES OF $^{14}CO_2$ PRODUCTION FROM $^{14}CH_3$-LABELLED HEXAMETHYLMELAMINE IN THE MOUSE.** A. Gescher & T. Raymont, *Cancer Chemotherapy Research Group, University of Aston, Birmingham*

The antineoplastic drug hexamethylmelamine (HMM) requires metabolic activation, and its activity depends on the presence of methyl moieties in the molecule. It is biotransformed in the liver by N-demethylation, following which the single carbon moiety enters the formaldehyde-formate pool and is mainly excreted as $CO_2$. As part of our investigation into the relationship between HMM metabolism and its mode of action we determined $^{14}CO_2$ exhalation in a continual fashion in CBA/Lac mice (20–30 g) following i.p. administration of $^{14}CH_3$-labelled HMM (40 mg/kg) and compared it with the $^{14}CO_2$ appearance in breath after 38 mg/kg $^{14}CH_3$-labelled aminopyrine (AP), a non-cytotoxic model substrate of N-demethylases. Whereas the $^{14}CO_2$ formation rate–time plot for AP peaked rapidly and declined in a biphasic fashion, the corresponding plot for HMM showed a peak after 40–70 min and a monophasic decline with a half life ($t\frac{1}{2}$) of $89{\cdot}7 \pm 6{\cdot}6$ min (n = 5). Peak N-demethylating activity produced $82{\cdot}5 \pm 8{\cdot}0$ nmol $^{14}CO_2$/min (n = 4) after AP and $35{\cdot}4 \pm 4{\cdot}5$ nmol $^{14}CO_2$/min (n = 4) after HMM. Pretreatment with an inhibitor of oxidative drug-metabolizing enzymes (SKF525A, 60 mg/kg i.p. 30 min before drug administration) and an enzyme inducer (phenobarbital, 50 mg/kg i.p. for 4 days) dramatically effected the $CO_2$ data. For HMM the $t\frac{1}{2}$ of the $^{14}CO_2$ in the breath after SKF was increased to $264{\cdot}5 \pm 21{\cdot}7$ min (n = 3); in phenobarbital-pretreated mice the $t\frac{1}{2}$ was decreased to $69{\cdot}3 \pm 10{\cdot}7$ min (n = 4). These results show that pharmacogenic interference with oxidative drug metabolism leads to changed HMM demethylation rates *in vivo* which may influence its antineoplastic efficacy.

**CLINICAL AND EXPERIMENTAL STUDIES WITH PENTAMETHYLMELAMINE (PMM).** D. R. Newell, C. J. Rutty, J. F. R. Muindi, I. E. Smith & K. R. Harrap, *Dept of Biochemical Pharmacology, Inst. of Cancer Res., Sutton, Surrey*

PMM is a water-soluble melamine derivative which, unlike hexamethylmelamine (HMM), can be administered clinically in parenteral form. In the present Phase I clinical study PMM was given as an i.v. infusion (100 mg/m$^2$–1300 mg/m$^2$). Gastrointestinal toxicity (nausea and vomiting) was the major side-effect observed, as with HMM given orally, which became severe and prolonged (24 h) at doses $>300$ mg/m$^2$. Pharmacokinetics and metabolism were monitored in patients by gas chromatography using a nitrogen-specific detector. PMM is eliminated from the plasma with a half-life of 108 min. Two major PMM metabolites were detected in plasma: $N^2N^2N^4N^6$-tetramethylmelamine and $N^2N^4$-$N^6$-trimethylmelamine. Pharmacokinetic analyses indicate that i.v. PMM produces higher levels of less extensively demethylated melamines than HMM administered orally. Hence it may be possible, using PMM, to achieve therapeutic levels of active metabolites at doses which are non-toxic to normal host tissues. Thus PMM may offer a significant therapeutic advantage over HMM. In addition, preliminary evidence from high-performance liquid chromatography analyses suggests the presence of N-methylolmelamines in human plasma. Such compounds are chemically unstable in aqueous solution, releasing formaldehyde at a rate which is highly dependent upon the ionic environment. N-methylolmelamines are more toxic to a number of tumour cell lines *in vitro* than are the corresponding methylmelamines, and thus may represent the species responsible for the *in vivo* antitumour activity. N-methylolmelamines markedly inhibit both DNA and RNA synthesis *in vitro*, as does formaldehyde. However, these inhibitory effects do not appear to be responsible for the selective cytotoxic action *in vivo*, where inhibition of nucleic acid synthesis in sensitive tumour cells cannot be shown.

**PRECLINICAL STUDIES ON POTENTIAL SECOND-GENERATION ALTERNATIVES TO CISPLATIN (CisPtII),** K. R. Harrap, M. Jones, R. Shepherd, H. McD. Clink, S. Sparrow, B. C. V. Mitchley, S. A. Clarke & A. Veasy, *Dept Biochem. Pharmacol., Inst. Cancer Res., Sutton, Surrey*

Cis-dichlorodiammine platinum (II) (cisplatin) is useful in the treatment of human malignant disease. However, its therapeutic effectiveness is limited by the appearance of severe dose-limiting toxic side-effects. These include nephrotoxicity, myelotoxicity, severe nausea and vomiting and ototoxicity. There is a great need to replace cisplatin with a less toxic derivative possessing antitumour activity at least comparable to that of cisplatin. To this end we have selected 8 platinum complexes from more than 300 derivatives previously screened for antitumour activity at the Institute of Cancer Research. These 8 compounds all possess antitumour activities comparable to or greater than that of cisplatin in several rodent tumour systems.

The selected platinum complexes were screened against a wider spectrum of transplantable animal tumours, and against a human epidermoid carcinoma of the bronchus grown in immune-deprived mice. Toxic damage to normal tissues was assessed in the rat at maximum tolerated doses of each compound. A biochemical indicator of tissue damage, platinum-induced nuclear protein phosphorylation, was also measured in tumour and several host tissues.

Our overall conclusion from this study is that cisdiammine-1,1-cyclobutane dicarboxylic acid (CBDCA) exhibits more selective antitumour activity than any of the other derivatives. This compound is a viable candidate for Phase I clinical evaluation, and an appropriate toxicology study is in hand.

**REDUCTION OF THE CYTOTOXICITY OF HYDROXYMETHYL TRIAZENES BY ESTER OR ETHER FORMATION.** J. A. Hickman, R. J. Simmonds, *Department of Pharmacy, University of Aston, Birmingham*, G. F. Kolar, *D.K.F.F.*, 6900 *Heidelberg, F.R.G.* & K. Vaughan, *Department of Chemistry, St Mary's University, Halifax, Nova Scotia*

The N-dimethyltriazines are metabolized to selective antitumour species, and it has been proposed that the hydroxymethyl intermediates of their metabolic N-demethylation may be important for selective cytotoxicity (Hickman, 1978, *Biochimie*, **60**, 997). Preussman *et al.* (1969, *Biochem. Pharmacol.*, **18**, 1) have suggested that conjugates of hydroxymethyltriazenes may be transport forms of biologically active species, and Kolar & Carubelli (1979, *Cancer Lett.*, **7**, 209) recently reported the isolation from rat urine of a novel O-glucuroniside conjugate after administration of 3,3-dimethyl-1-(2,4,6-trichlorophenyl)triazene. We have now investigated the cytotoxic and antitumour properties of this conjugate. In an *in vivo* antitumour test against the TLX5 lymphoma in CBA/LAC mice 50 mg/kg of the conjugate had no activity whereas 50 mg/kg of the precursor dimethyltriazene gave up to 62% increase in life span. *In vitro* the conjugate had no toxicity at 1 mg/ml, whereas the monomethyl analogue was potently cytotoxic at 50 $\mu$g/ml. Our failure to synthesize the hydroxymethyl analogue prevented a direct comparison between this compound and its conjugate; however, *p*-carbomethoxy-N-hydroxymethyl-N-methylphenyltriazene was active *in vivo*, whereas its O-benzoylated derivative had reduced activity. The results suggest that substitution of hydroxymethyltriazines may be a deactivation step in the metabolism of dimethyltriazenes.

**THE TOXICITY OF BINARY COMBINATIONS OF 2′-DEOXYCOFORMYCIN (dCf) AND 2′-DEOXYADENOSINE (AdR).** R. M. Paine, H. McD. Clink, K. G. McGhee & K. R. Harrap, *Dept Biochem. Pharmacol., Inst. Cancer Res. and Dept Haematology, Royal Marsden Hospital, Sutton, Surrey*

A Phase I clinical study of dCf showed inhibition of lymphocyte adenosine deaminase (ADA) activity, lymphocytotoxicity and large elevation in erythrocyte deoxyadenosine triphosphate (dATP). The latter effect reflected increased circulating purine levels following treatment, and suggested that AdR might be used clinically to enhance dCf activity. We have previously shown that dCf potentiates the cytotoxicity of AdR *in vitro* (Paine *et al.*, 1977, *Br. J. Cancer*, **36**, 417). The underlying biochemical mechanism appeared to be *via* the intracellular accumulation of dATP, a known negative effector of the enzyme ribonucleotide reductase. The present study shows, however, that dCf/AdR combination can prove prohibitively toxic to normal tissues.

Treatment of mice with dCf (0·27 mg/kg) for 5 days inhibited spleen and lymphnode ADA, a 64-fold elevation of erythrocyte dATP, but no haematological toxicity or weight loss. However, if mice were injected with AdR (270 mg/kg) in combination with dCf, weight loss, haematological toxicity and liver damage occurred. Leucopenia was pronounced, often with agranulocytosis. The femoral marrows of these mice showed cytotoxic damage, including vacuolation and some giant metamyelocytes. Erythrocyte dATP levels were raised 1500-fold. Mortality during treatment was attributable to acute liver failure; marrow toxicity was only detected when treatment had finished. No such toxic effects were seen in mice receiving adenosine (AR, 270 mg/kg) in combination with dCf, AR alone, or AdR alone. We conclude that the supplementation of clinical dCf schedules with AdR should be considered with caution.

**THE EFFECTS OF NANDROLONE DECANOATE (DECA - DURABOLIN, ORGANON) ON THE TOXICITY AND ANTI-TUMOUR ACTION OF CCNU IN EXPERIMENTAL MOUSE COLON TUMOURS.** J. A. Double & M. C. Bibby, *Postgraduate School of Studies in Medical and Surgical Sciences, University of Bradford*

There are conflicting reports in the literature on the value of anabolic steroids in the management of patients undergoing cancer chemotherapy. This situation almost certainly arises from variation in dose and treatment schedules. Most reports describe simultaneous treatment with low doses of anabolics, which appear to be of no value. The Bradford group have maintained that in order to achieve clinical benefits it is necessary to pretreat the patients with high doses of anabolics (Hancock *et al.*, 1977, *Br. J. Surg.*, **64**, 134). Clinically, nandrolone decanoate (N.D.) appears to reduce the toxicity of various cytotoxic agents. The present study is an attempt to transfer a clinical impression to numerical data, using an experimental model system. The spectrum of chemotherapeutic sensitivity to standard agents of the mouse adenocarcinoma of the colon (MAC) system has been shown to be similar to human large-bowel cancer (Double & Ball, 1975, *Cancer Chemother. Rep.*, **59**, 1083). Therapeutic indices are low and responses are only seen close to maximum tolerated dose. We have shown that 7-day pretreatment with N.D. raises the $LD_{50}$ of CCNU from 78 mg/kg to 112 mg/kg. It could be argued that this was a generalized reduction in the potency of the cytotoxic agent. Results from combined toxicity and anti-tumour studies using the MAC 13 line and CCNU however have shown that the anti-tumour action of the cytotoxic agent is unaltered by the presence of N.D. Simultaneous treatment with N.D. and CCNU produces a therapeutic index of 1·1, whereas 7-day pretreatment with N.D. before CCNU produces a therapeutic index of 1·7. Further studies on this phenomenon are under way using other agents and other tumour lines.

**EFFECT OF PREDNISOLONE ON CHROMATIN BINDING OF ALKYLATING AGENTS.** P. J. Thraves, R. Shepherd. A. Jeney & K. R. Harrap, *Dept of Biochem. Pharmacol., Inst. of Cancer Res., Sutton, Surrey*

Prednisolone enhances the selectivity of chlorambucil by reducing its systemic toxicity and enhancing tumour-cell kill (Harrap *et al.*, 1977, *Eur. J. Cancer*, **13**, 873). The present studies demonstrate that although prednisolone does not enhance the overall macromolecular binding of $^{3}H$-chlorambucil *in vivo*, it does modify its binding to specific chromatin fractions in a tissue-selective manner. Binding was *increased* in chromatin fractions of both alkylating-agent sensitive and resistent strains of Walker 256 carcinosarcoma, but *reduced* in the corresponding fractions from intestinal mucosa. *In vitro*, prednisolone enhanced the binding of $^{14}C$-mephalan to soluble chromatin in resistant cells, but did not alter gross macromolecular binding. The highest concentration of bound drug was in the insoluble chromatin fraction. About 30% of chromatin-bound drug was released after DNase II digestion. Chlorambucil treatment enhanced the digestion of chromatin by micrococcal nuclease, as demonstrated by sucrose-density-gradient fractionation. Chlorambucil appeared to bind preferentially to internucleosome chromatin.

These data demonstrate that the enhanced selectivity of chlorambucil in the presence of prednisolone is associated with tissue-specific modulations of chromatin binding, and that internucleosome linker regions and insoluble structural chromatin are major alkylating-agent-binding sites.

**CYTOSINE ARABINOSIDE (ARA-C) DEAMINATION IN HUMAN LEUKAEMIC MARROW AND RESISTANCE TO ARA-C.** A. L. HARRIS & D. G. GRAHAME-SMITH, *M.R.C. Clinical Pharmacology Unit, Radcliffe Infirmary, Oxford.* (Introduced by Prof. A. J. S. Davies)

Ara-C is an effective drug in acute myeloid leukaemia (AML). Used alone remission occurs in only 25–47% of patients (Armentrout & Burns, 1974, *Am. J. Med. Sci.*, **268,** 163). Ara-C is converted intracellularly to its active metabolite Ara-CTP in AML myeloblasts, and also deaminated to Ara-U, an inactive metabolite. High deaminase activity in lysed marrow blasts has been associated with clinical resistance to Ara-C (Steuart & Burke, 1971, *Nature* (*New Biol.*), **233,** 109) but this was not found with intact peripheral blasts (Smyth *et al.*, 1976, *Eur. J. Cancer*, **12,** 567).

Intact marrow myeloblasts from 11 patients with newly diagnosed untreated AML were incubated with ($^3$H)-Ara-C (1 nM–100 $\mu$M) for 45 min at 37°C in Eagle's medium.($^3$H)-Ara-CTP and ($^3$H)-Ara-U production was measured, with and without 1mM tetrahydrouridine (THU) a potent deminase inhibitor. The sensitivity of DNA synthesis to Ara-C was measured using inhibition of ($^3$H)-TdR incorporation into DNA by 10nM–10$\mu$M unlabelled Ara-C, incubated under identical conditions ($\pm$THU). The ratios of Ara-U/Ara-CTP ranged from 0·32 to 19·11. THU increased Ara-CTP production by 0–27% and deamination was completely inhibited. The increase in Ara-CTP was not related to Ara-U/Ara-CTP ratio, but to % deamination of medium in absence of THU (Corr. 0·89, $P<0{\cdot}001$). 50% inhibition of DNA synthesis was produced by 30–100nM Ara-C, and at 1 $\mu$M 80% inhibition occurred in all blasts. THU did not increase the sensitivity to Ara-C.

These results show that Ara-C was freely available to both metabolic pathways, and that deamination does not produce resistance to Ara-C by diverting Ara-C intracellularly from the kinase pathway that produces Ara-CTP.

**THE DESIGN AND SYNTHESIS OF A NOVEL FOLATE ANTIMETABOLITE N-(4-(N-((2-AMINO-4-HYDROXY-6-QUINAZOLINYL) METHYL) PROP-2 YNYLAMINO)BENZYL)-L-GLUTAMIC ACID (CB 3717).** T. R. JONES, A. H. CALVERT, A. L. JACKMAN & K. R. HARRAP, *Dept Biochem. Pharmacol., Inst. Cancer Res., Sutton, Surrey*

Critical examination of the folate enzyme network suggests the need for a folate inhibitor of thymidylate synthetase (TS) which should give an antimetabolite superior both to methotrexate (MTX) and to 5-fluorouracil (FU) for the following reasons: (i) TS is the rate-limiting enzyme in the thymidylate cycle; (ii) the antipurine effect of MTX, causing gut toxicity in mice but contributing little to the antitumour effect, is avoided (Harrap *et al.*, 1977, *Chem. Biol. Interact.*, **18,** 119); (iii) a folate analogue does not require metabolic activation; resistance to FU is frequently due to the deletion of its activating kinase; (iv) unlike FU, there should be no incorporation into RNA with an associated toxicity; (v) the competing metabolite, 5,10-methylene-tetrahydrofolate, lies at a metabolic branch point and might not build up to overcome the block; (vi) Having a different locus of action from MTX the agent should be active against MTX-resistant cells which have raised intracellular dihydrofolate reductase.

We have achieved this aim, and report here the design and synthesis of 2 new 2-amino-4-hydroxy-quinazoline antifolates. Progressive unsaturation of the terminal C-C bond of a 3-carbon substituent at N-10 increases the affinity of the compounds for TS. The 50% inhibitory concentrations were: 170nM (propyl); 69nM (allyl); 5nM (propargyl). This last named, CB 3717, is the most potent inhibitor of TS yet described. Some of its biochemical and antitumour properties are outlined in the next paper.

**BIOLOGICAL PROPERTIES OF A NOVEL FOLATE ANTIMETABOLITE (CB 3717).** A. H. CALVERT, A. L. JACKMAN, T. R. JONES & K. R. HARRAP, *Dept Biochem. Pharmacol., Inst. Cancer Res., Sutton, Surrey*

This compound was developed as an antitumour agent acting by inhibition of thymidylate synthetase (TS, EC 2.1.1.45). The inhibition of TS (purified by affinity chromatography from L1210 cells) by CB 3717 was characterized, and found to be competitive with 5,10-methylenetetrahydrofolate. The $K_i$ was 1–2nM, making CB 3717 the most potent TS inhibitor described. Reversal of cytotoxicity to L1210 cells in culture could be achieved by co-incubation with thymidine, suggesting that TS was the effective locus. A cell line (L1210/R7A), resistant to methotrexate (MTX) due to raised intracellular dihydrofolate reductase (DHFR) was not significantly cross-resistant to CB 3717. Since MTX resistance may also occur by reduction of the active transport of MTX, the transport of CB 3717 was investigated. It appeared that CB 3717 may be transported by a different pathway to MTX (possibly that used by folic acid) since: (a) CB 3717 competed with labelled folic acid for transport into L1210 cells; and (b) A cell line, L1210/R3U, resistant to MTX due to both increased DHFR and decreased MTX transport, was not substantially cross-resistant to CB 3717. Dramatic antitumour effects, with minimal toxicity, were observed in animals bearing the L1210 tumour. The ascitic tumour was cured in 90% of animals, and significant increases in lifespan were seen after i.v. injection of the tumour. In both cases the results were better than those obtained with an optimal schedule of MTX. Activity was also seen against the ADJ/PC6 tumour, which is normally found to be refractory to antimetabolite treatment. Toxicological evaluation of CB 3717 is planned with a view to early clinical study.

### CHEMOSENSITIVITY OF HUMAN BREAST - TUMOUR XENOGRAFTS.

M. J. Bailey, I. Smith & G. Steel, *Biophysics Department, Institute of Cancer Research, Sutton, Surrey*

Eight lines of human breast cancer have been established and serially transplanted in immune-deprived mice. 5 of these lines have been tested for chemosensitivity to the cytotoxic agents clinically active against breast cancer, using tumour growth delay as an end-point. The tumours varied considerably in their drug sensitivity, some lines being generally chemosensitive, whilst others showed little response to any agent. The order in which drugs and drug combinations were ranked remained constant in repeated experiments on each line, but differed from one line to another. In all cases, a combination of drugs was more effective than the single agents in that combination at equitoxic doses. By studying dose–response curves of single agents, new drug combinations may be designed. One such combination (adriamycin plus melphalan) proved to be very effective when tested against 3 of the xenograft lines. This system may be useful in testing new agents and predicting new combinations for clinical use against breast cancer.

### ENHANCEMENT OF RAZOXANE ANTITUMOUR ACTIVITY BY CIMETIDINE.

M. M. Collins (introduced by K. Hellman), *Department of Cancer Chemotherapy, Imperial Cancer Research Fund, London WC2*

Razoxane [±1,2 di (3,5-dioxopiperazine-1-yl) propane] is a non-selective inhibitor of mitosis (Bakowski, 1976, *Cancer Treat. Rev.*, **3**, 95). It is active by mouth and soluble in dilute HCl. To investigate whether gastric acidity had any influence on the subsequent fate and activity of the compound on experimental tumours, cimetidine was given in anticipation that the antitumour effect of razoxane would be appreciably diminished. In the event the opposite occurred.

Female Sprague–Dawley rats were inoculated with Walker carcinosarcoma and pretreated with cimetidine 1 h before administration of razoxane. The antitumour effect of razoxane in these rats was significantly greater than in those treated with razoxane alone ($P < 0{\cdot}05$). Cimetidine alone possessed no significant antitumour activity. The experiments have been repeated 4 times and the results have been similar on each occasion.

It is clear therefore that gastric acidity is not important to the antimitotic activity of razoxane. Whether the enhanced activity of razoxane after cimetidine might be due to increased blood levels of razoxane or other mechanisms is not yet resolved.

### METHOTREXATE WITH INOSINE, THYMIDINE AND ALLOPURINOL RESCUE. A PHASE I CLINICAL STUDY.

P. J. Dady, G. A. Taylor & K. R. Harrap, *Dept of Biochem. Pharmacol., Inst. of Cancer Res., Sutton, Surrey*

Mice are rescued from the toxic effects of methotrexate (MTX) by a combination of inosine, thymidine and allopurinol (ITA); antitumour activity is superior to that of MTX with folinic acid (FA) rescue (Harrap *et al.*, 1977, *Chem.-Biol. Interactions*, **18**, 119). 32 patients were treated in a Phase I clinical study of ITA rescue. Initially ITA, without MTX, was given to 9 patients. Then 400 mg/$m^2$/24 h MTX was infused into 17 patients and ITA, in decreasing doses, was given at the end of these infusions. A "minimum safe rescue" was identified. In 6 patients the dose of MTX was then increased in stages to 1·5 g/$m^2$/24 h.

No toxicity was seen with ITA alone, nor when MTX 400 mg/$m^2$/24 h was followed by rescue infusions of more than: inosine 0·2 g/$m^2$/24 h, TdR 1 g/$m^2$/24 h and allopurinol 200 mg 8-hourly. Below this "minimum safe rescue", myelosuppression, mucositis and skin rashes were seen, even though rescue was continued until circulating MTX had fallen to $6 \times 10^{-8}$M. With 800 mg/$m^2$/24 h MTX "minimum safe rescue" was successful, but when MTX 1·5 g/$m^2$/24 h was used, toxicity occurred in spite of rescue. MTX-related skin lesions were more frequent than would be expected with the MTX plasma levels achieved. This study showed that ITA is not in itself toxic and the "minimum safe rescue" is effective from MTX doses up to at least 800 mg/$m^2$/24 h.

### A VERSATILE HIGH-PERFORMANCE LIQUID CHROMATOGRAPHIC (HPLC) METHOD FOR THE ESTIMATION OF PURINES, PYRIMIDINES AND THEIR CONGENERS.

G. A. Taylor, P. J. Dady, A. S. Grimes & K. R. Harrap, *Dept of Biochem. Pharmacol., Inst. of Cancer Res., Sutton, Surrey*

A method was developed for the estimation of purines, pyrimidines and their congeners in the plasma of patients included in a Phase I clinical study of thymidine (TdR)/inosine (IR)/allopurinol (Ap) (ITA) reversal of methotrexate (MTX) toxicity. An initial isocratic reverse-phase HPLC separation allowed the collection of a number of effluent fractions, each containing a single component of interest, together with a limited number of unknown contaminants. the reapplication of these fractions to a second HPLC separation permitted the resolution and quantitation of the components of interest.

Circulating levels of nucleosides and bases were measured in patients' plasmas, and in samples from healthy human volunteers. Patients' pretreatment levels of hypoxanthine (HX) fell into 2 distinct groups: the majority (20/25) were in the range 0·85–6·5 $\mu$M, but 5/25 ranged between 49·8 and 64·2 $\mu$M (mean $52·4 \pm 2·3$). The "low" HX group (mean $2·62 \pm 0·32$ $\mu$M) had significantly higher levels of circulating HX ($P = 0·006$) than healthy humans ($1·62 \pm 0·2$ $\mu$M). Following its oral administration, Ap was rapidly metabolized to oxypurinol, which circulated at high levels ($\sim 10^{-4}$M) for considerable periods ($>6$ h). During the administration of the "minimum safe rescue" of ITA, no significant elevation of HX, thymine (T) or TdR was observed. At higher rescue doses TdR was metabolized to T with a half-life of 14–20 min; HX possessed a half-life of 40–65 min.

The method has a high sensitivity; it has also been applied to enzyme assays (*e.g.* xanthine oxidase, dihydrothymine dehydrogenase) and to the purification of labile radioactive materials.

### DETECTION OF RADIOIODINATED TUMOUR - SPECIFIC ANTIGENS WITH SYNGENEIC ANTISERA.

D. Hannant*, J. G. Bowen†, M. R. Price* & R. W. Baldwin*, **Cancer Research Campaign Laboratories, University of Nottingham and †Boots Co., Nottingham*

Lectin affinity and immunoadsorbent chromatography were used to purify papain-solubilized tumour-specific antigens (TSA) from the aminoazo dye-induced rat hepatoma D23. Isolated antigens were radioiodinated using procedures which included (a) coupling of $^{125}$I-labelled tyrosine to TSA with a carbodiimided reagent, (b) protection of antigenic determinant by labelling TSA while bound to its syngeneic immunoadsorbent. These radiolabelled antigens retained serological activity, as demonstrated by co-precipitation (double antibody) tests and specific rebinding to syngeneic immunoadsorbents. Immunoadsorption chromatography indicated that one consequence of radio-labelling hepatoma D23 TSA with $^{125}$I was to reduce the affinity of the labelled antigen for its syngeneic specific antibody.

### DETECTION OF HOST IMMUNE RESPONSE TO A TRANSPLANTABLE TUMOUR IN THE RAT BY THE LAI ASSAY.

C. M. BALLARD & J. P. DICKINSON, *YCRC Laboratories, University Department of Radiotherapy, Cookridge Hospital, Leeds*

The modified capillary-tube leucocyte adherence inhibition (LAI) assay (Ballard & Dickinson, 1980, *Clin. Exp. Immunol.*, **40**) has been used to monitor host immune response to a transplantable mammary carcinoma of spontaneous origin in the rat (Moore & Dixon, 1977, *Cell Tissue Kinet*, **10,** 583). Animals were inoculated with either viable or non-viable (irradiated) minced tumour. Blood was collected by cardiac puncture at various times after inoculation. 3M KCl extracts of tumour and liver were used in the assay. Leucocytes from control animals (untreated, or injected with 0·9% NaCl only) showed a weak, nonspecific, concentration-dependent inhibition of adherence with both extracts, similar to the responses obtained when other proteins (*e.g.* KLH, PPD, FCS, BSA) are added to a leucocyte suspension. A very strong additional inhibition of leucocyte adherence with tumour extract was obtained soon after inoculation in both tumour-bearing and irradiated-tumour-bearing animals. This was maintained throughout the experimental period in irradiated-tumour-bearing animals, but declined as the tumour enlarged in tumour-bearing animals. Throughout the period, responses of both groups of animals to liver extract remained similar to responses of control animals. No LAI effect was observed when leucocytes from control animals were preincubated with plasma from tumour-bearers and irradiated-tumour-bearers. The results show a specific recognition of tumour by the host; however, recognition does not imply an effect on tumour growth. Heavy tumour load was found to lead to abrogation of this response. The LAI assay has been found to be a simple and reliable method for detection and monitoring of one aspect of the host cellular response to the presence of malignant disease.

### PRESENCE OF A TUMOUR IN $F_1$ HYBRID MICE PARTIALLY INHIBITS THE INDUCTION OF GVHD AFTER INJECTION OF PARENT LINE ICC.

T. WHITMARSH-EVERISS & M. O. SYMES, *Department of Surgery, University of Bristol*

In (AxCBA(T6)$F_1$ hybrid mice, GVHD was induced by i.v. injection of A spleen cells, on Day 0. One half of the hybrid mice had received an s.c. transplant of an (AxCBA)$F_1$ strain tumour on Day −9. The mice were killed on Day 14 and their spleen ratios (wt of spleen/g body wt) and spleen indices (spleen ratio of mouse receiving parent-line cells/mean spleen ratio of uninjected mice or mice receiving tumour cells only) determined. The presence of a tumour reduced GVHD in 2 experiments (see Table).

There was no difference in tumour weight, at Day 14, between the several groups of tumour-bearing animals receiving spleen cells. Thus inhibition of GVHD may not depend on diversion of the spleen cells to attack the tumour.

| Dose of A spleen cells injected | Exp. 1 Mean spleen index | | | Exp. 2 Mean spleen index | | |
|---|---|---|---|---|---|---|
| | No tumour | Tumour | *P* | No tumour | Tumour | *P* |
| 50 N | 1·49 | 1·14 | NS | | | |
| 100 N | 2·15 | 1·67 | <0·05 | | | |
| 50 Im | 1·86 | 1·55 | NS | 1·58 | 0·87 | <0·001 |
| 100 Im | 2·22 | 1·38 | <0·001 | 1·94 | 1·30 | <0·001 |
| 150 Im | | | | 2·53 | 1·82 | <0·001 |

N = non-immune. Im = immune to (AxCBA)$F_1$ tumour. 5–8 animals/group.

### REGIONAL IMMUNOTHERAPY OF GUINEA-PIG LINE-10 HEPATOMA WITH CHEMICAL HYPERSENSITIZERS.

M. V. PIMM*, R. W. BALDWIN*, W. A. BASLEY* & V. S. BYERS†, **Cancer Research Campaign Laboratories, University of Nottingham and †Dept Dermatology, Univ. of California, U.S.A.*

Regional immunotherapy in which agents are administered to localize within tumour deposits and promote local host responses, including hypersensitivity reactions, is being widely evaluated for cancer therapy. Originally intact bacteria (principally BCG and *C. parvum*) were used, but these have toxic side-effects, and attention is now directed to developing more defined agents.

The present studies are evaluating regional immunotherapy with alkylcatechols related to the natural plant oil urushiol. These were chosen because they are highly potent hypersensitizing agents with marked lipophilicity (Byers *et al.*, 1979, *J. Clin. Invest.*, **64**, 1437). With intact viable rat and guinea-pig hepatoma cells, $^{3}$H-3-n-pentadecylcatechol (PDC) became incorporated into cell membranes on *in vitro* incubation. Intradermal growth of the guinea-pig Line-10 hepatoma was restricted by intralesional injection of PDC or urushiol, and there was a concomitant control of lymphnode metastases. Although effective in DMSO solution, the response was considerably enhanced when PDC was given in a lipid solvent (squalene). Pre-induction of contact sensitivity to PDC also enhanced the therapeutic response.

These studies demonstrate that chemically defined agents, inducing T-lymphocyte-mediated delayed-hypersensitivity responses, can be used for regional tumour immunotherapy.

### SUPPRESSOR CELLS IN RATS IMMUNIZED AGAINST SOLUBILIZED HEPATOMA-SPECIFIC ANTIGENS.

M. R. Price, D. Hannant, J. G. Bowen & R. W. Baldwin, *Cancer Research Campaign Laboratories, University of Nottingham*

Currently available evidence strongly favours the view that immunization with solubilized tumour-specific antigens from the aminoazo dye-induced rat hepatoma D23 preferentially induces suppressor lymphoid cells. Similar cells are also demonstrable in the thymus of hepatoma-bearing rats, since these cells have the capacity to abrogate tumour-specific transplant-rejection immunity in immune rats. This effect has been reproduced with the soluble fraction of lysates of thymus cells from rats treated with soluble 3M KCl extracts of tumour, indicating the involvement of a tumour-specific soluble suppressor factor. In order to interrupt the maturation of precursor suppressor lymphoid cells, rats were treated with cyclophosphamide (40 mg/kg) before immunization with soluble hepatoma D23 antigen preparations. Animals receiving such treatment were subsequently found to be protected against s.c. challenge with viable tumour cells, a response which has formerly not been achieved with acellular rat hepatoma-specific antigens.

### CONCANAVALIN-A-INDUCED SUPPRESSOR ACTIVITY OF LYMPHOCYTES DERIVED FROM HODGKIN'S DISEASE SPLEENS.

A. Akbar, D. B. Jones, S. V. Payne and D. H. Wright, *University Department of Pathology, General Hospital, Southampton*

Marked changes in lymphocyte subpopulations occur in the spleen in Hodgkin's disease before histological evidence of lymphoma involvement, and have been implicated in the pathogenesis of the condition (Payne *et al.*, 1976, *Clin. Exp. Immunol.*, **24**, 280).

We report results from a preliminary study of concanavalin-A (Con-A)-inducible non-specific suppressor cells present in lymphocytes extracted from spleen tissues. T- and B-enriched lymphocyte suspensions from spleens taken at Hodgkin's staging laparotomy are optimally stimulated with Con-A for 24 h, washed thoroughly in the presence of $\alpha$-methyl manopyranoside and then mixed at a 1:3 ratio with normal allogeneic peripheral-blood responder cells stimulated with Pokeweed mitogen (PWM). Immunoglobulin production at 7 days is measured by immunofluorescence on acetone-fixed cytocentrifuge preparations. Control cultures consist of splenic lymphocytes pre-cultured without Con-A and responder cells cultured alone.

In 4 HD spleens, Con-A-prestimulated T-enriched lymphocyte preparations (range 81–92% sheep-rosetting cells) gave 40–60% suppression of PWM-induced immunoglobulin production relative to control prestimulated cells ($P < 0 \cdot 01$). Two B-enriched preparations (70 and 76% surface Ig$^{+}$ cells) failed to suppress under identical conditions. The requirements for suppressor induction in spleen are different from those for peripheral blood lymphocytes. Whilst these preliminary data are insufficient to enable comment on alterations in suppressor-cell function in HD, we present the results as a viable technique for the measurement of this T-cell function in lymphocytes extracted from malignant and pre-malignant tissue.

**AUTOREACTIVE LYMPHOCYTES IN COLONIC CARCINOMA.** B. M. VOSE*, M. MOORE*, P. GALLAGHER† & P. F. SCHOFIELD†, **Department of Immunology, Patterson Laboratories and* †*Department of Surgery, Withington Hospital, Manchester*

The cytotoxicity of lymphocytes from blood, mesenteric lymph node and tumour have been tested for cytotoxicity against autologous colon tumour cells. A short term (4 h) $^{51}$Cr-release assay was used. Significant cytotoxic reactivity was recorded in 15/23 cases, with most of the positive reactions occurring in Dukes' Stages B and C. Blood lymphocytes from 2/15 healthy individuals were also reactive. In Stage B blood lymphocytes were cytotoxic more frequently than node lymphocytes. Nodes distant from the tumour induced damage in only 1/10 cases, whereas those within 2 cm of the neoplasm were positive in 4/13 patients. In Stage C reactivity in blood and proximal and distal mesenteric lymph nodes occurred together. When present, cytotoxicity could be concentrated in cells rosetting with sheep erythrocytes or by filtration through nylon-fibre columns.

Lymphocytes isolated from 6 tumours showed killing of neither autologous tumour nor the K562 cell line, even in cases where effectors from other sites were reactive. Concentration of tumour-infiltrating T cells by passage through nylon columns did not induce cytotoxicity. Autoreactive cytolytic lymphocytes can thus be detected in colonic neoplasia. The association of these with the clinical course of disease and prognosis is currently under investigation.

**THE INTESTINAL CYTOTOXIC CELL RESPONSE TO AN i.p. INOCULATED ALLOGENEIC TUMOUR.** M. D. J. DAVIES & D. M. V. PARROTT, *Dept of Bacteriology and Immunology, Western Infirmary, Glasgow*

Responses to i.p. inoculated tumours in organized lymphoid tissues and the peritoneal cavity are well documented. Mucosal surfaces, which frequently contain many lymphocytes, have received less attention. Using a new preparation technique, it has now been possible to examine lymphoid cells of the lamina propria of murine small intestine. 5 days after i.p. injection of P-815 tumour cells into C57BL mice, there is a very high level of specific cytotoxic T cells in the lamina propria, which cannot be explained by contamination by Peyer's patch cells. At this time, the specific cytotoxic T-cell levels in other organs are at very low levels. The lamina propria cytotoxic cell levels remain high, while those throughout the lymphoid system increase. A pertinent question is whether this phenomenon is similar to the preferential localization of intestinally activated lymphocytes in the intestine rather than in peripheral sites.

**CHARACTERIZATION OF MACROPHAGE SUB-POPULATIONS WITHIN AN IMMUNOGENIC MURINE FIBROSARCOMA.** K. MOORE & W. H. MCBRIDE, *Department of Bacteriology, Edinburgh University Medical School*

Peritoneal-exudate macrophages can be fractionated by velocity sedimentation at unit gravity into sub-populations differing in their states of activation as determined by FC-receptor avidity in the binding of IgG-sensitized erythrocytes, to form EA rosettes. Activation was associated with macrophages sedimenting at 5–9 mm/h, whilst those sedimenting at 1–5 mm/h were less activated and contained a higher proportion of peroxidase-positive cells.

The sedimentation profile of EA-rosette-forming cells within cell suspensions prepared from the immunogenic fibrosarcoma FSA-R was identical to the profile of peritoneal-exudate cells, and isolation of tumour-infiltrating macrophages indicated at least 2 sub-populations.

Comparison of tumour macrophages with peritoneal macrophages indicates that the highly activated, rapidly sedimenting fraction is in the same state of activation as corresponding *Corynebacterium parvum*-activated macrophages, and both are more highly activated than proteose/peptone-elicited macrophages. The less rapidly sedimenting tumour-macrophage fraction was more highly activated than the corresponding *C. parvum* fraction, which was in turn more activated than proteose/peptone-elicited macrophages.

Thus the macrophage component of the host-cell infiltrate within a solid tumour is a heterogeneous population composed of different stages of differentiation.

**IMMUNOPEROXIDASE STAINING OF AN ANTI-NCA-FREE ANTI-CEA IMMUNOGLOBULIN WITH LUNG TUMOURS ONLY AFTER TRYPSINIZATION.** C. H. J. FORD, A. J. SALTER & C. E. NEWMAN, *Clinical Oncology Unit, University of Birmingham, Queen Elizabeth Hospital, Birmingham*

An absorbed anti-CEA Ig (donated by IDRL, University of Birmingham), shown to localize *in vivo* (Dykes *et al.*, 1980, *Br. Med. J.*, i, 220) has been further absorbed with a glutaraldehyde-fixed polymer of normal human spleen to remove anti-NCA antibodies. Immunoperoxidase (Heyderman & Neville, 1977, *J. Clin. Pathol.*, **30**, 138) was used to investigate cellular localization after absorption. Chronic myeloid leukaemia cells or sections of formalin-fixed spleen were used as targets, as they express NCA. After absorption, the Ig still contained a high titre of anti-CEA antibodies in an enzyme-linked immunosorbent assay. However, by immunoperoxidase it failed to stain a lung tumour previously known to localize anti-CEA antibodies, and showed only weak, restricted staining of liver metastases containing CEA, or a breast or colon tumour, all of which had been strongly positive before absorption. Only after treatment of sections with 0·1% trypsin for 2 h was it possible to demonstrate tumour-cell staining in sections from 15/27 lung-cancer patients. Of 11 patients who had raised serum CEA levels (>40 ng/ml) preoperatively, 9 were positive; of 10 who developed raised levels postoperatively, 4 were positive; of 6 who had normal levels throughout follow-up, 2 were positive. Although an absorbed non-immune sheep Ig did not stain tumour cells after 2h trypsinization, both this, and the absorbed anti-CEA Ig, showed considerable staining of collagen. As little is known of the effect of trypsin on antigenic determinants in tissue sections, these preliminary results must be treated with caution. However, they illustrate the problems of working with a highly absorbed anti-CEA Ig, and raise the possibility that some reports of anti-CEA localization *in vitro* in lung tumours may be due to specific anti-NCA antibodies, or antibodies directed at antigenic determinants common to both CEA and NCA.

**LIMITED SPECIFICITY OF AN ANTI-LUNG TUMOUR ANTISERUM IN A FUSED-ROCKET IMMUNOELECTROPHORESIS (FRIEP) TECHNIQUE.** C. S. WOODHOUSE, C. H. J. FORD & C. E. NEWMAN, *Clinical Oncology Unit, University of Birmingham, Queen Elizabeth Hospital, Birmingham*

An antiserum raised to a cultured human small-(oat-)cell carcinoma line (OCCI) (Ford & Newman, 1979, *Carcino-Embryonic Proteins*, **2**, 541) was investigated using FRIEP. Extracts prepared from normal lung and lung tumour tissues were used as a reference panel against which antibody specificity could be judged, by comparison with the pattern produced by "monospecific" antisera. The unabsorbed antiserum showed relatively high titre reactivity against no more than 4 antigens in any one extract and produced no precipitates against human serum in crossed-immunoelectrophoresis.

Using this antiserum, an antigen has been demonstrated in a saline extract of a squamous-cell lung carcinoma from the panel of extracts. The antigen is not detectable by FRIEP in extracts of normal lung adjacent to the tumour, of a second squamous-cell carcinoma nor of several normal lung tissues. Comparison with antisera to 25 serum proteins (CEA, AFP, Ferritin, retinol-binding protein, HCG $\alpha$ unit, SPI, PAG or tumour-associated antigens described by Veltri (Veltri *et al.*, 1977, *Cancer Res.*, **37**, 1313), Irie (Irie *et al.*, 1976, *Cancer Res.*, **39**, 2902) and Gennings (Gennings *et al.*, 1979, *Carcino-Embryonic Proteins*, **2**, 553) has failed to identify the antigen. It is soluble in 50%-saturated ammonium sulphate and shows an $\alpha_2$ electrophoretic mobility in agarose at pH 8·6. The presence of this antigen in extracts of several other lung tumours is now being examined.

The ability to identify the uniqueness of this antigen by the unabsorbed antiserum and its limited reactivity are notable, since xeno-antisera raised to tumour cells characteristically require extensive absorption before selectivity can be shown.

### *IN VITRO* CYTOTOXICITY OF AN ANTI-CEA IMMUNOGLOBULIN WITH CULTURED LUNG TUMOUR CELLS.

J. R. JOHNSON, C. E. NEWMAN & C. H. J. FORD, *Clinical Oncology Unit, University of Birmingham, Queen Elizabeth Hospital, Birmingham*

A high-titre anti-CEA Ig, supplied by the Immunodiagnostic Research Laboratory (IDRL), University of Birmingham, has previously been shown to localize on human tumours *in vivo* (Dykes *et al.*, 1980, *Br. Med. J.*, i, 220). Using indirect immunofluorescent and immunoperoxidase techniques, the same Ig shows surface and cytoplasmic localization with cells from a human anaplastic lung-tumour line (Calu-6). A temporal variation exists with the proportion of cells staining in the immunofluorescence test, being highest in the lag phase of culture growth and decreasing during the log phase.

In a $^{51}$Cr-release assay, the same Ig is cytotoxic to Calu-6 target cells used immediately after trypsinization, when $\sim 20\%$ of cells show localization of the Ig. This effect is augmented by the addition of rabbit complement, but abolished by prior heat inactivation of the Ig (56°C, 30 min).

Although vincristine exhibits no immediate lysis, in the presence of heat-inactivated Ig or heat-inactivated Ig and complement its cytotoxicity is greatly increased and apparently dose-dependent. Similar effects with adriamycin, 5-fluorouracil, bleomycin and methotrexate have not been obtained.

The specificity of these interactions is under investigation.

### BIOLOGICAL ACTIVITY OF MONOCLONAL ANTIBODY TO A SPONTANEOUS RAT MAMMARY CARCINOMA.

M. J. EMBLETON, J. G. MIDDLE & G. R. FLANNERY, *Cancer Research Campaign Laboratories, University of Nottingham*

A tumour-specific monoclonal antibody was prepared against a rat mammary carcinoma, Sp4, by the production and cloning of a hybrid between mouse myeloma cells (P3-NS1) and spleen cells from an Sp4-immune rat. This antibody was detected by $^{125}$I antiglobulin binding, and further studies have been undertaken to determine its activity in tumour transplantation *in vivo*, and in complement-dependent and cell-dependent cytotoxicity tests *in vitro*. These studies are aimed at evaluating the therapeutic potential of monoclonal antibodies to tumour cells.

### ARYL AZIDES AS RADIOSENSITIZING AGENTS: A PRELIMINARY PHOTO-CHEMICAL EVALUATION.

M. F. G. STEVENS & C. K. WONG, *Cancer Chemotherapy Research Group, Department of Pharmacy, University of Aston in Birmingham*

Aryl azides undergo photolytic and radiolytic degradation to transient reactive species (arylnitrenes) capable of versatile interactions with biological substrates (Knowles, 1972, *Accounts Chem. Res.*, **5**, 155). The effects of 4 azides [9-(3-azidoanilino)acridine (1), 9-(4-azidoanilino)acridine (2), 9-(4-azido-2-methoxyanilino)acridine (3) and 4-azidobenzenesulphonamide (4)] on L-1210 cells in RPMI medium were examined in the dark and during exposure to light (366 nm) for 5 min. Cell numbers were measured with a Coulter Counter and cytotoxicity was expressed as $ID_{80}$ ($\mu$g/ml); each result is a mean of three determinations.

Cytotoxicity of azides to L-1210 cells

| Compound | $ID_{80}$ (dark) | $ID_{80}$ (light) |
|---|---|---|
| (1) | 61 | 5·5 |
| (2) | 11 | 0·9 |
| (3) | 11 | 2·2 |
| (4) | $>1\times10^3$ | 4·3 |

Results indicated that irradiation enhanced cytotoxicity of the drugs in all cases, but the most dramatic effect was elicited by the azidosulphonamide (4) which was non-cytotoxic in the dark bu significantly cytotoxic when incubated with L-1210 cells in light. Pre-irradiation of the azidosulphonamide solution before exposure to L-1210 cells abolished activity ($ID_{80} > 1\times10^3$ $\mu$g). In control experiments the stable photoproducts formed from azidosulphonamide were found to be non-cytotoxic, and it is possible that a photogenerated nitrene species is the destructive agent.

### EFFECTS OF DEXAMETHASONE ON THE PHARMACOKINETICS AND TOXICITY OF MISONIDAZOLE IN MICE.

P. WORKMAN, *MRC Clinical Oncology and Radiotherapeutics Unit, Cambridge*

Previous studies (Workman, 1979, *Br. J. Cancer*, **40,** 335) have shown that pretreatment of mice with phenobarbitone or phenytoin increased the acute $LD_{50}$ of the hypoxic cell radiosensitizer misonidazole (Ro 07-0582; MISO). The mechanism involves an increased rate of metabolism to desmethylmisonidazole (Ro 05-9963; DEMISO) through induction of hepatic microsomal enzymes, which decreases the area under the curve (AUC) for MISO in blood and brain. Clinical studies are in progress to assess the possible role of phenobarbitone and phenytoin, and also the corticosteroid dexamethasone, in reducing MISO neurotoxicity.

Experiments were carried out to investigate the interaction between dexamethasone sodium phosphate (DEX) and MISO in C3H mice. MISO was injected i.p. at a dose of 1 g/kg and MISO and DEMISO were measured in blood and brain by high-performance liquid chromatography (Workman *et al.*, 1978, *J. Chromatogr.*, **147,** 507). Pretreatment with DEX (0·5, 25 and 100 mg/kg i.p. daily for 5 days) had no effect on the metabolism, pharmacokinetics and urinary excretion of MISO. Simultaneously administered DEX (25 mg/kg i.v.) did not alter the blood concentrations of MISO and DEMISO, but did reduce the brain MISO AUC by 15% ($P < 0·02$).

Simultaneous DEX did not change the acute $LD_{50}$ for MISO (1·8–1·9 g/kg). However, this was reduced to 1·7 g/kg after pretreatment with 0·5 mg/kg/day DEX, and decreased further at the higher doses of DEX.

**DRUG INTERACTIONS WITH MISONIDAZOLE IN THE DOG.** R. A. S. White, *Dept. Clin. Vet. Medicine, and* P. Workman, *Clinical Oncology and Radiotherapeutics, Cambridge*

The hypoxic cell radiosensitizing drug misonidazole (MISO) is currently being evaluated in several clinical trials, but its total dose has been found to be limited by neurotoxic side-effects. We have investigated in the dog the interactions of several drugs likely to be used in combination with MISO which may alter its pharmacokinetic behaviour and possibly its efficacy and toxicity.

Cyclophosphamide (Cy 50 mg/m$^2$ every 2nd day for 9 wks) was found to delay oral absorption and reduce peak plasma MISO concentrations by 30%, but bioavailability was generally unimpaired. Half-life was extended by up to 35% in 1 dog.

Phenytoin sodium (15 mg/kg daily pretreatment for 10 days) peak plasma concentrations were reduced by 10%, plasma half-life by 40% and total tissue exposure by 35%. Pretreatment with phenobarbitone achieved a similar effect, although simultaneous sodium pentobarbitone increased peaks by 10% and tissue exposure by 30%.

Dexamethasone (0·1 mg/kg daily for 9 weeks) was found to have no effect on MISO pharmacokinetics.

**RESPONSE TO CHEMOTHERAPY OF MULTICELLULAR TUMOUR SPHEROIDS IN STATIC CULTURE** P. R. Twentyman, *MRC Clinical Oncology and Radiotherapeutics Units, Hills Road, Cambridge*

Multicellular tumour spheroids of several cell lines have been grown using a static culture technique modified from that of Yuhas *et al.* (1977, *Cancer Res.*, **37,** 3639). These lines include the EMT6 and RIF-1 mouse tumours and the HT29 and CaMa-1 human colon and breast carcinomas. Response of the growing spheroids to cytotoxic drug treatment *in vitro* has been studied using both spheroid growth delay and clonogenic cell survival as endpoints. In EMT6 spheroids, time of assay after drug treatment was an important parameter in the determination of cell survival. Apparent recovery from potentially lethal damage occurred after treatment with nitrogen mustard (NH2), BCNU, CCNU, myleran and cis-platinum. For this group of agents a reasonably similar relationship was found between spheroid growth delay and cell survival (measured 24 h after treatment). For adriamycin, actinomycin D and 5 fluorourcil, however, extended growth delay was observed with relatively little cell kill. The human cell spheroids were similar to EMT6 in sensitivity to HN2 (as assayed by growth delay) but markedly less sensitive to BCNU. The relationship between spheroid growth delay and cell survival in RIF-1 and HT29 spheroids is currently being investigated.

Many features of the tumour spheroid model system make it amenable to the investigation of tumour-response parameters in a way that cannot be done with simpler monolayer cultures.

**STUDIES WITH HYPERTHERMIA AND CYTOTOXIC DRUGS USING EMT6 MULTICELLULAR TUMOUR SPHEROIDS.** J. E. Morgan & N. M. Bleehen, *MRC Clinical Oncology and Radiotherapeutics Unit, Cambridge*

Multicellular tumour spheroids show many characteristics of *in vivo* tumours which are not present in monolayer cultures. In these studies spheroids of the EMT6/Ca/VJAC cell line have been used as a relatively sophisticated *in vitro* model system to investigate interactions between hyperthermia and some commonly used cytotoxic agents. Heating was carried out by total immersion in a thermostatically controlled circulating waterbath. The response of the spheroids to treatment has been assayed, both in terms of growth delay and of surviving fraction after trypsinization. Using spheroids 200–300 $\mu$m in diameter we have demonstrated a significant enhancement of bleomycin cytotoxicity at 43°C. For example, a growth delay of 0·40 days seen after exposure to 10 $\mu$g/ml BLM for 1 h at 37°C is increased to 3·05 days at 43°C. Heat alone under these conditions produces a growth delay of 0·50 days. Preheating at 40°C has previously been shown to alter the response to EMT6 cells to subsequent heat and drug exposures. Similarly with spheroids, a prior exposure of 6 h at 40°C induces tolerance to subsequent BLM (20 $\mu$g/ml) at 43°C/1 h, reducing the growth delay from 5·95 days to 3·70 days. With adriamycin, no increased cytotoxicity was seen after a 1h exposure at 43°C. However, prolonged exposure (6 h) to 1 $\mu$g/ml adriamycin increased the growth delay from 1·10 days at 37°C to 4·15 days at 42°C. Current investigations into the heat and drug sensitivities of spheroids pretreated with 5mM MISO at 37°C under both oxic and hypoxic conditions were also reported.

**EFFECT OF HIGH BLOOD-SUGAR LEVELS ON BLOOD FLOW AND THERMAL RESPONSES OF ANIMAL TUMOURS.** S. K. Calderwood & J. A. Dickson, *Cancer Research Unit, Dept of Clinical Biochemistry, Royal Victoria Infirmary, Newcastle upon Tyne*

There is now considerable evidence that several types of animal and human tumours are damaged by heating at temperatures >42°C, while normal tissues are unaffected. The current work concerns approaches whereby this heating effect may be potentiated.

Blood flow in the Yoshida sarcoma (measured by rubidium-86) was inhibited by 90–100% at high blood-glucose levels (>20mM). A similar finding was obtained in MC-7 sarcoma and D-23 carcinoma. The inhibition in each tumour was independent of site, occurring in s.c. tumours on the foot and flank and in i.m. leg tumours, and was similar at a range of tumour volumes. A similar inhibition was produced by galactose, a sugar not metabolized by the tumours. In the i.m. VX2 carcinoma in the rabbit, blood flow was also inhibited by hyperglycaemia. Blood flow in normal tissues of the animals was little affected by hyperglycaemia.

The inhibition of blood flow enhanced the specificity of thermotherapy; the tumour was unable to dissipate heat *via* circulatory blood, whilst normal tissues were cooled by a heat-induced increase in blood flow which was unaltered by the increase in blood sugar. Tumour heating was more uniform at high sugar levels and the use of such conditions, combined with heat, may eliminate the incidence of "cool zones" around tumour blood vessels.

If inhibition of blood flow at high blood-sugar level proves a general phenomenon in tumours, it may represent an Achilles' heel for selective therapy in cancer cells isolated from the host.

**CIRCULATING IMMUNE COMPLEXES IN DOGS WITH OSTEOSARCOMA.** A. Segal-Eiras, R. A. Robins, L. Owens & R. W. Baldwin. *Cancer Research Laboratories, University of Nottingham*

It is known that some dog malignant diseases present similar characteristics to those found in humans (Ankerst *et al.*, 1973, *Bull. W.H.O.*, **49**, 205). The present study shows the results from the $^{125}$I-C1q binding assay in an attempt to analyse circulating immune complexes (CIC) in dogs bearing osteogenic sarcoma (OS). Serum and plasma samples from adult large dogs with OS, normal Irish wolfhounds and beagles were tested for CIC by the C1q-binding test. A high proportion (36/43; 83%) of the OS sera showed high C1q-binding levels. These results were compared with those obtained in normal dog samples, where C1q values were low. The

Clq-binding technique has been slightly modified in this laboratory by the use of heparin (Baldwin *et al.*, 1979, *Behring Inst. Mitt.*, **64,** 63) which increases the discrimination between normal and pathological samples in the study on human tumours. Correlative determination of Clq-binding levels in serum and heparinized plasma showed that the addition of heparin increased Clq binding in normal dog samples with a smaller increase in Clq binding with plasma samples from dogs with OS. The investigations on dogs' sera revealed a very clear-cut difference of Clq-binding levels between the tumour-bearing animals and the normal, without any addition of heparin. Five dogs with OS were studied for CIC when samples were taken before and after treatment. Post-therapy serum samples showed a decrease of Clq-binding levels in all cases, and so far these dogs remain without clinical evidence of disease. It is tempting to consider that the Clq-binding test may be a useful method of studying CIC in dogs with osteogenic sarcoma.

### CIRCULATING IMMUNE COMPLEXES IN HUMAN BONE NEOPLASIA.

A. Segal-Eiras, R. A. Robins, R. W. Baldwin & V. S. Byers, *Cancer Research Laboratories, University of Nottingham*

Serum samples from patients bearing osteogenic sarcoma (OS), giant cell tumour (GCT), osteoblastoma and healthy controls were studied for the presence of circulating immune complexes (CIC) by the $^{125}$I-Clq-binding assay, slightly modified by the inclusion of heparin (Baldwin *et al.*, 1979, *Behring Inst. Mitt.*, **64,** 63). A high proportion (42/62; 67·7%) of the OS sera showed high Clq binding. Similarly, $^{125}$I-Clq-binding assay with sera from GCT patients revealed a high proportion with elevated Clq binding levels. These results were compared with those obtained with sera from patients with benign osteoblastoma, and with 20 sera from normal young donors. In the benign bone-tumour group none of the sera tested showed high Clq binding, and of the normal young donors samples, only 1 serum gave a significantly high Clq binding. Serum samples from 2 patients were available for limited study of sequential changes in serum Clq binding. In one patient, high Clq binding was seen preoperatively and in the immediate post-operative period, with values subsequently decreasing, remaining in the normal range for 4 years; this patient continues free of clinical evidence of disease. In a second patient, Clq binding returned to normal for only a brief period after amputation, followed by consistently high Clq binding; this patient died of metastic disease. There is some suggestive evidence that some of the Clq binding is due to tumour-related CIC. A finding of note in the present studies is the elevation of CIC in OS, but none in benign osteoblastoma or normal young donors; similar results were obtained using the heparin-modified Clq-binding assay for comparing breast carcinoma with benign breast diseases.

### DETECTION OF IMMUNE COMPLEXES IN SERA FROM PATIENTS WITH CHRONIC LUNG DISEASES, USING THE Clq-BINDING ASSAY

K. M. Cooper, M. Moore & A. M. Hilton, *Paterson Laboratories, Christie Hospital, Manchester*

Levels of Clq-binding activity (Clq BA) in the sera of patient with various chronic lung diseases and healthy controls, were measured by the technique of Zubler & Lambert (1976, *J. Immunol.*, **116,** 232) under conditions where (i) the limit of detection of human aggregated IgG was 5 $\mu$g (absolute amount) and (ii) there was freedom from interference by DNA and LPS. Clq BA for 50 controls gave an upper limit of 4·50% (mean + 2 s.d.) levels above which in patients' sera were considered positive. Four main groups of patients were studied, whose Clq BA status was as follows.

| | No. pos./ No. tested (%) | ClqBA mean ± s.d. (range) |
|---|---|---|
| Ca bronchus | 33/60 (55) | 9·77 ± 14·23 (1·53–78·40) |
| Bronchiectasis | 19/29 (66) | 9·33 ± 7·31 (1·60–24·05) |
| Chronic bronchitis | 7/37 (19) | 3·49 ± 2·12 (1·64–12·16) |
| Asthma | 4/22 (18) | 3·20 ± 1·24 (1·90–5·94) |

There was no correlation between Clq BA and serum levels of C-reactive protein, C3, C4, or IgM among the patients with Ca bronchus and bronchiectasis. However, there was a positive correlation with IgA and IgG levels. The data indicate that Clq BA in sera is associated with both degenerative and neoplastic conditions of the lung. It is suggested that C1q BA in these diseases may be a reflection of an autoimmune response to antigens released during the course of tissue degeneration/proliferation.

### *IN VITRO* REACTION OF CANCER PATIENTS AND OTHERS TO BCG.

A. J. COCHRAN*, G. TODD†, C. W. CHAN§, R. D. KENNEDY†, R. MACKIE‡ & L. MORRISON†, **Surgical Oncology, UCLA, †Western Infirmary, Glasgow and §Queen Mary Hospital, Hong Kong*

Recall-antigen skin testing is useful for assessing immune competence. However, serial monitoring of cancer patients is impractical because of interpretative problems from reimmunization by repeated antigen exposure. We investigated *in vitro* BCG reactivity in 68 cancer patients and 121 controls (disease-free or with non-debilitating, non-malignant conditions) from a highly tuberculin-sensitized population. A one-stage capillary leucocyte (blood) migration inhibition assay was used. "Antigen" was Glaxo BCG at 25, 37·5, 50, 75 and 100 ug/ml. The reaction frequency of cancer and control groups increased with increasing BCG concentration, but patients were always less reactive than controls. Maximum separation was at 37·5 and 50 $\mu$g/ml of BCG. Cancer patients' reactivity declined significantly with advancing disease. Stage I patients were similarly reactive whether or not tumour was present. Stage II patients with tumour present were less frequently reactive (4/12) than those with tumour (8/12). A small decline in reactivity occurred in controls over 60 years old. The *in vitro* test correlated well with the Mantoux test; 8/11 controls gave concurrent concordant results. In cancer patients from a highly tuberculin-reactive population and in BCG recipients, this simple assay performed serially may aid the prediction of metastases.

### IMMUNOCYTOCHEMICAL LOCALIZATION OF OESTROGEN RECEPTORS

J. HUMPHREYS*, J. H. WESTWOOD†, M. G. ORMEROD†, R. C. COOMBES* & A. M. NEVILLE* (Introduced by G. C. Easty), **Ludwig Institute for Cancer Research and †Institute for Cancer Research, Sutton, Surrey*

The main purpose of the immunocytochemical localization of oestrogen receptors (ER) is to replace a cumbersome radiochemical assay with a simple histological technique. We have extensively evaluated 3 methods: (1) Conjugation of oestradiol ($E_2$) to immunoglobulin G (IgG) or horseradish peroxidase (HRPO). IgG-conjugated $E_2$ was applied to a tissue section in a similar manner to the first antibody in an immunoenzymatic technique. An antiserum raised against $E_2$IgG, and conjugated with HRPO, was then applied to frozen sections of ER-rich tissues (including breast carcinomas), as in an immunoperoxidase protocol. Staining was obtained, but further studies are necessary to determine its specificity. Alternatively, conjugation of $E_2$ directly to HRPO eliminated the second step, but produced a less encouraging result. (2) A "triple sandwich" technique using anti-oestradiol serum. In this method ER is localized by applying $E_2$, anti-$E_2$ and HRPO-linked second antibody in sequence. Early results were positive, but nonspecific staining could not be excluded, since the first antiserum may have contained unwanted antibodies. (3) Immunofluorescence using fluoroscein isothiocyanate (FITC)-conjugated $E_2$. Two methods, after Pertschuk *et al.* (1979, *Am. J. Clin. Pathol.*, **71**, 504) and Lee (1979, *Cancer*, **44**, 1) were followed in an attempt to detect ER by immunofluorescence. $E_2$ was linked to FITC *via* bovine serum albumen. With Pertschuk's method, fluorescence in $ER^+$ tissues was obtained routinely, but we could find no evidence that this was specific. A conjugate made after Lee had very poor binding ability to ER on assay. We conclude that immunocytochemical techniques as currently used produce results that should be interpreted most cautiously. This may be due to the unstable and very soluble nature of the ER molecule, and studies are under way to overcome these factors.

### DETECTION OF MICROMETASTASES OF MAMMARY CARCINOMA USING AN IMMUNOHISTOCHEMICAL STAIN FOR EPITHELIAL MEMBRANE ANTIGEN (EMA).

M. G. ORMEROD*, J. P. SLOANE†, S. F. IMRIE*, K. STEELE*, R. C. COOMBES‡ & A. M. NEVILLE‡, **Institute for Cancer Research, Royal Cancer Hospital, †Royal Marsden Hospital, ‡Ludwig Institute for Cancer Research, Royal Marsden Hospital, Sutton, Surrey*

The human epithelial membrane antigen (EMA) has been found on the surface or luminal membranes of most non-squamous epithelium (Heyderman *et al.*, 1979, *J. Clin. Pathol.*, **32,** 35). All lymphoid tissues were negative. The antigen is produced by most carcinomas.

In mammary carcinomas, any lumina formed invariably stained for EMA. In many tumours it was also found in the cytoplasm, particularly in single cells in infiltrates or metastases. An immunohistochemical stain for EMA has been used in sections of fixed tissues to detect micrometastases which could not be recognized by conventional histology.

We have stained sections of aspirates of marrow from patients with metastatic mammary carcinoma. 10% of the aspirates which had been read as negative on an H.+E. section were shown to contain metastatic cells.

### IDENTIFICATION OF DIFFERENT CELL TYPES USING TWO ENZYME REACTIONS MEASURED SIMULTANEOUSLY IN SINGLE CELLS WITH A MULTI-PARAMETER FLOW CYTOMETER.

S. H. Chambers & J. V. Watson, *MRC Clinical Oncology Unit, The Medical School, Cambridge*

A prerequisite for the eventual use of flow cytometers in clinical medicine is the ability to distinguish reliably between the different cell types that are always present in any biopsy sample. It would also be advantageous to be able to obtain sorted functionally active cells for further manipulations. Most staining procedures, apart from vital DNA staining with benzimidazole derivatives, surface-marker labelling and those using fluorescein diacetate (FDA) result in cell death. By assaying simultaneously with the fluorogenic esterase substrates 4-methylumbelliferyl acetate (MUA) and FDA it has been possible to distinguish between a number of different cell types. Furthermore, in 2 tumour-cell lines in which plating experiments have been carried out, the cloning ability has been retained at above 90% for MUA and FDA exposures of up to 6 h. Both these compounds are hydrolysed nonspecifically by esterases; thus at least two enzymes may be present in each cell type, each with different specificities and reaction-rate characteristics for these substrates.

### PHYSICO-BIOCHEMICAL PROPERTIES OF SUBPOPULATIONS IN MOUSE MARROW.

J. V. Watson & S. H. Chambers, *MRC Clinical Oncology Unit, The Medical School, Cambridge*

Preliminary studies have been conducted on mouse marrow with a multiparameter flow cytometer. Blue light scattered from unstained cells in the foward direction (4°–8°) and measured simultaneously with that scattered at right angles (85°–93°) from each cell revealed 4 distinct, possibly 5 clusters. Similar 2-dimensional studies with UV light showed at least 6 different clusters. Twin enzyme reactions measured simultaneously with 4-methylumbelliferone acetate (UV excitation, violet analysis) and fluorescein diacetate (blue excitation, green analysis) have shown at least 8 different populations with their different reaction-rate characteristics. Although some populations are likely to be common to each pair of the 3 assay modalities it is likely that some are not. The combined assay using all 6 parameters simultaneously has not yet been made, but we hope it will be possible to identify marrow stem cells within this 6-dimensional assay space and to cell sort for positive functional identification after the instrument has been upgraded.

### A TWIN LASER MULTI-PARAMETER ANALYSING FLOW CYTOMETER.

J. V. Watson, *MRC Clinical Oncology Unit, The Medical School, Cambridge*

Most laser-based flow cytometers lack fluorescence excitation versatility, due to the limited number of lasing lines that can be obtained with a single laser. Even with a large tunable laser only one wavelength can be obtained at a time, which considerably reduced the potential of such instruments. These problems have been overcome in our unit by the design and construction of a flow cytometer with a small argon laser emitting blue light at 488 nm, which is used in conjunction with a large krypton laser. The latter can be tuned to emit in the UV, violet, green, yellow or red and any one of these lines can be used for excitation either sequentially or simultaneously with the blue line from the argon laser. Narrow-angle forward light scatter at two wavelengths, plus 90° light collection and analysis in the UV, violet/low blue, high blue, green and red wavelength bands can be achieved simultaneously giving 7-parameter analysis. The principles of operation, design specifications and preliminary performance data were described.

**MEASUREMENT OF LECTIN BINDING TO HUMAN MYELOID AND LYMPHOID CELLS USING FLOW CYTOMETRY.** G. Blackledge, J. Gallagher, A. Morris & D. Crowther, *CRC Department of Medical Oncology, Manchester University and Christie Hospital, Manchester*

Lectins are proteins, derived mainly from plant tissues, which bind reversibly to monosaccharides of defined configuration and structure. In the oligosaccharide chains of glycoproteins, lectins recognize principally, but not exclusively, sugar residues at nonreducing termini. Using flow cytometry we have investigated the binding of 3 fluorescent-conjugated lectins (ConA, WGA, LCA) to the surface membrane of a human erythroleukaemia cell line, K562. By this method it was found that incubation of cells with lectin at 4°C (to prevent endocytosis) for 30 min gave maximal binding and reproducible measurement of surface fluorescence. Specificity of binding was measured by incubation with appropriate sugar inhibitors ($\alpha$ methyl mannoside for ConA and LCA and N-acetyl glucosamine for WGA) and 50–80% of the lectin fluorescence was abolished under these conditions. Dialysed serum which contains glycoproteins was also an effective inhibitor of lectin binding. Using unlabelled lectins, competition studies of lectin binding were carried out. Although LCA and ConA bind to similar sugars, unlabelled ConA at high concentrations could only inhibit 70% of the FITC–LCA binding. This suggests the existence of high-affinity sites for LCA on the surface of K562 cells.

Differences in lectin binding have been seen between different human cell types. Lymphocytes and lymphoma cells have fewer binding sites for ConA on their surface than myeloid and myeloid leukaemia cells. Cells from chronic lymphocytic leukaemia have fewer binding sites for ConA than normal lymphocytes. Lectin binding may prove of clinical relevance as a prognostic factor in the lymphomas.

**CELL KINETICS OF PERTURBATIONS OF SKIN, MARROW AND TUMOUR CELLS INDUCED BY HIGH-DOSE INFUSION OF HYDROXYUREA IN PATIENTS WITH BREAST CANCER.** M. Lambert & H. Bush, *CRC Department of Medical Oncology, Christie Hospital and Holt Radium Institute, Manchester*

Four patients with advanced breast cancer were treated with a 72h continuous infusion hydroxyurea (HU) at 6–12 g/day. Biopsy specimens of marrow, skin and tumour were taken before the infusion was begun and again at 72, 78 and 84 h. The [$^3$H]-TdR-labelling index (LI) of these specimens was determined by the method of Meyer & Bauer (1975) with ARG for 10 days, and in addition the S-phase proportion of the marrow was determined by flow cytofluorimetry after nuclear staining with propidium iodide. In patients in whom adequate serum levels of HU were sustained (>0·1mM), the LI of the marrow was reduced by the infusion from a pretreatment level of 7–15% (mean 12%) to 2–6% (mean 4·5%) at 72 h. After the infusion was discontinued a rebound phenomenon was seen with a marrow LI at 78 h of 12–25% (mean 20%) and at 84 h of 15–22% (mean 18%). Similar values for the S-phase proportion were obtained by cytofluorimetry. The pretreatment LI of the skin specimens was 1·0–2·5% (mean 1·6%) and at 72 h it was reduced to 0·2–0·6% (mean 0·4%) and again a rebound was seen at 78 h (LI 2·5–14%, mean 6·2%) and at 84 h (LI 15–22%, mean 18%). The tumour specimens, however, showed little perturbation, the pretreatment LI was 4·7–16% (mean 10%), at 72 h 9–16% (mean 12%), and at 78 h 9–22% (mean 15%). Attempts at preferential *in vivo* synchrony of normal human tumour cell populations have been partially successful. The biological and technical problems associated with this technique will be discussed. This and similar techniques (Ara-C infusion) offers the possibility of selective protection of dividing skin, marrow, and gut cell populations in cancer patients treated with cytotoxic drugs and radiotherapy.

**SOMATIC CELL HYBRIDIZATION STUDIES ON THE NATURE OF CELLULAR RESISTANCE TO ICRF 159 (RAZOXANE).** D. H. EDGAR & A. M. CREIGHTON, *Cellular Pharmacology Laboratory, Imperial Cancer Research Fund, London*

Two sublines of BHK 21S cells with independently induced resistance to ICRF 159 have been fused with drug-sensitive lines and the drug responses of the resultant somatic cell hybrids have been characterized. Fusion of either of the resistant lines with drug-sensitive Syrian hamster cells yields intraspecific hybrids which demonstrate intermediate levels of drug resistance in colony-forming assays. Since resistance is not due to impaired uptake of ICRF 159 (White & Creighton, 1976, *Br. J. Cancer*, **34,** 323) this result suggests that these resistant lines have either high levels of a drug-specific target or a target with decreased drug susceptibility.

The characteristic perturbation of cell-cycle distribution which results when ICRF 159 is present in a cell population has been investigated in sensitive, resistant and hybrid cells treated with the drug. We have found in the lines examined an inverse correlation between the degree of perturbation caused by a single dose of ICRF 159 and the resistance index of the cell line, as determined by colony-forming assays. In this system therefore flow cytofluorimetry can be used to give an early indication of response to ICRF 159.

**CYCLOSPORIN A IN TUMOUR-BEARING RODENTS.** S. E. HECKFORD, S. A. ECCLES & P. ALEXANDER, *Division of Tumour Immunology, Institute of Cancer Research, Sutton, Surrey*

Cyclosporin A (Sandoz Ltd) is a new immunosuppressive agent with little myelotoxicity, which is being used to replace corticosteroid treatment in kidney-transplant patients and to suppress GVH disease in marrow-graft recipients. In view of the current interest in Cyclosporin A (CYA) we are investigating its effects on the biology of a variety of rodent tumours.

CYA will allow allograft tumours to grow progressively, but in the syngeneic host it has no detectable effect on the biological characteristics of the primary tumour implant. If CYA is administered during the growth of fibrosarcomas in mice or rats, the incidence of metastases following tumour resection is significantly greater than in control groups. We are currently investigating the effect of CYA on rechallenge immunity, as we have found that administration of CYA after excision of the primary tumour implants also facilitates the growth of disseminated tumour cells. In contrast, CYA does not appear to alter the incidence of metastases in animals that have borne skin or mammary carcinomas.

**MACROPHAGES AND METASTATIC POTENTIAL IN MOUSE MAMMARY TUMOURS.** J. R. G. NASH, J. E. PRICE & D. TARIN, *Department of Histopathology, John Radcliffe Hospital, University of Oxford*

Individual tumours contain different proportions of non-neoplastic cells. Some of these are stromal, whilst others possibly have a protective function, mediated immunologically or otherwise. It has previously been reported that *transplantable* tumours of high metastatic potential contain fewer macrophages than ones incapable of dissemination. However, the processes of tumour transplantation and of maintaining tumours in culture probably alter their properties, and we have therefore chosen to re-examine the relationship of macrophage content to tumour spread, using *primary* mammary tumours in mice.

Ingestion of latex particles proved too nonspecific for reliably identifying macrophages in tumour-cell suspensions, and histochemical staining for enzymes such as lysosome, acid phosphatase and nonspecific esterase proved even more limited in value. We therefore used the Fc-mediated phagocytosis assay, in which antibody-coated sheep red cells are ingested by the macrophages under controlled conditions, to quantitate tumour macrophage content expressed as a proportion of total cells in the tumour suspension. With this we found moderate variation between tumours, both in the proportion of macrophages and in their phagocytic capacity.

The metastatic colonization potential of the tumours was assayed by necropsy of mice

90 days after i.v. inoculation of the disaggregated tumour cells, and the results for individual tumours was correlated with their macrophage content at the time of inoculation. It was found that there is no correlation between macrophage content and the colonization potential of these primary tumours.

### EFFECT OF INHIBITION OF PLATELET FUNCTION ON SEEDING OF CIRCULATING TUMOUR CELLS.

N. WILLMOTT, A. BAXTER & K. C. CALMAN, *Department of Clinical Oncology, University of Glasgow, and* A. MALCOLM, *Department of Pathology, Western Infirmary, Glasgow*

The accretion of platelets has been postulated to facilitate the seeding of metastatic tumour cells. To test whether this hypothesis applied to the TLX-5 murine lymphoma, the occurrence of blood-borne metastases was mimicked by injecting a low number (1–5 $\times 10^2$) of TLX-5 lymphoma cells i.v. Tumour spread was not confined to the first vascular bed encountered, the lungs, but was also evident in liver, kidney and spleen. An attempt to prevent the seeding of injected lymphoma cells was made by injecting an inhibitor of platelet function, RA233 (30 mg/kg) i.v. 3 min before the lymphoma cells. This procedure had no effect on the survival time of tumour-bearing mice, which were all dead within 12 days regardless of treatment.

In separate experiments it was shown that RA 233 could inhibit platelet function. Thus, the drop in circulating platelet count caused by *Corynebacterium parvum* injected i.v. could be prevented if RA233 (30 mg/kg) was injected i.v. 3 min earlier. That is to say, although prior treatment with RA233 could prevent the platelet aggregation caused by *C. parvum*, it did not affect survival time when injected before tumour cells. It is therefore concluded that platelet accretion is not necessary for the seeding of i.v. injected TLX-5 lymphoma cells.

### LEWIS LUNG CARCINOMA: SELECTING METASTATIC VARIANTS.

M. MAGUDIA, P. WHUR, J. LOCKWOOD, J. BOSTON & D. C. WILLIAMS, *Marie Curie Memorial Foundation, Oxted, Surrey*

A stable line of Lewis lung carcinoma was maintained in mice by i.m. passage of cells from pooled primary tumours. Various parameters were monitored: (1) Latent period. (2) Growth rate. (3) Number of lung metastases. (4) Differential cell counts of primary tumours and metastases.

To obtain a high-metastasis variant the procedure was repeated substituting cells from lung tumours. The latent and the growth periods increased with each generation, and by the second passage differed significantly from those of the control group, indicating a slower primary tumour growth rate with each successive generation. The number and size of metastases increased after the first passage ($75 \pm 10$ compared to $55 \pm 1$ for stable line ($P < 0{\cdot}01$)). In both lines primary tumours contained more tumour cells and less macrophages than metastases, but the high-metastasis varient contained fewer macrophages at both sites than the stable line. This suggests a lowered immune response to the variant.

Low-metastasis variants were obtained by adapting cells to monolayer culture. Only a few tumour cells survived to form established cultures, and these were reinjected into mice. The latent and the growth periods increased substantially, and there were fewer metastases in the lungs. However, cells of metastatic origin produced significantly more metastases ($22 \pm 4$) than primary cells ($9 \pm 4$) ($P < 0{\cdot}05$).

These findings indicate that cells with varying metastatic potentials exist within primary tumour cell populations. Cells of high metastatic variants can be isolated by appropriate experimental procedures.

### METASTATIC COLONIZATION POTENTIAL OF PRIMARY TUMOURS INOCULATED BY DIFFERENT ROUTES.

D. TARIN & J. E. PRICE, Department *of Histopathology, John Radcliffe Hospital, University of Oxford*

In previous studies on primary mammary tumours we found that cells from some could heavily colonize the lungs after i.v. inoculation in mice, whereas those from others did so weakly or not at all. The tumours were all adenocarcinomas and we could find no features which correlated with this behavioural variation. However, the high reproducibility of the findings in each batch of animals inoculated with a given tumour indicated that the differences between individual tumours were due to intrinsic differences in their constituent cells.

To see whether interplay between these intrinsic properties and local environmental factors in organs containing tumour cells affects colony formation, we inoculated cells from each of 23 primary mammary tumours by 4 different routes. The routes chosen were *via* the tail vein or the hepatic portal vein or s.c. or i.p., and for each we used a separate batch of mice. The animals were killed and necropsied at 90 days and the distribution of deposits recorded.

In this experiment, comprising over 250 animals, it was found that, first, tumours which consistently colonized by one route of inoculation did not necessarily do so by all routes and, secondly, the combination of organs in which the inoculated tumour cells grew varied from tumour to tumour. These observations indicate that failure or success in colonization of the lungs after i.v. inoculation is not simply a matter of tumour transplantability, and is determined by interplay between intrinsic properties of the tumour cells themselves and local host influences in the sites where they lodge.

**A TECHNIQUE FOR THE STUDY OF LYMPHOCYTE RECIRCULATION IN HUMANS USING INDIUM$^{111}$ LABELLING.** J. Wagstaff, C. Gibson, N. Thatcher, W. L. Ford* & D. Crowther, *CRC Department of Medical Oncology, Christie Hospital, and *the Medical School, Manchester University, Manchester*

Indium$^{111}$ oxine has been shown to be an efficient label for lymphocytes (Rannie *et al.*, 1977, *Clin. Exp. Immunol.*, **29**, 509). It allows external gamma imaging (Frost *et al.*, 1979, *Int. J. Nucl. Med. Biol.*, **6**, 60) not possible with chromium. Only one report (Lavender *et al.*, 1977, *Br. Med. J.*, ii, 797) has been published applying this technique to humans. We have labelled lymphocytes obtained from peripheral blood using the Haemonetics Cell Separator. Lymphocyte migration from the blood to the liver, lungs, spleen and lymph nodes was followed using surface-probe counting, gamma-camera imaging and blood sampling. Normal volunteers and patients with chronic lymphocytic leukaemia, lymphoma and solid tumours have been studied. Preliminary results show imaging of spleen and lymph nodes. Blood curves in normals are similar to results previously reported using chromium (Jønsson & Christensen, 1978, *Scand. J. Haematol.*, **20**, 319). In chronic lymphocytic leukaemia and lymphoma, two patient groups have been distinguished, one with slower-than-normal blood clearances as previously reported (Bremer *et al.*, 1978, *Lymphology*, **2**, 231) and the second faster. Using dynamic gamma-camera imaging, new information on the early kinetics of reinjected lymphocytes has been obtained. Data showing activity changes with time in various organs (heart, lung, liver and spleen) have been obtained with the help of computer storage and analysis. These techniques allow the study of lymphocyte recirculation in humans by non-invasive means.

**TREATMENT OF LOCALIZED MALIGNANT LYMPHOMA OF THE ORBIT.** G. Thomas (introduced by Dr P. M. Wilkinson), *Department of Clinical Pharmacology, Christie Hospital, Manchester*

Twenty-three patients (mean age 61—range 5–87) with histologically proven orbital lymphoma received treatment during the period 1946–1975. Because of the long period, detailed evaluation was not possible in all cases, but clinically all patients had localized disease with normal peripheral-blood count and chest X-rays. The median duration of follow up is 126 months (range 44–318). Nine patients are alive with no further evidence of disease, one patient had a relapse successfully treated at 100 months, 10 patients died of incidental causes, 2 were lost to follow up (at 120 and 136 months) and in one patient it was impossible to determine the cause of death. The ratio of observed/expected deaths was 0·66. Although this particular site is said to carry a poor prognosis it would appear that, in patients with localized disease, treatment with radiotherapy alone is satisfactory.

**IMPROVED RESULTS OF TREATMENT OF GASTROINTESTINAL LYMPHOMA BY COMBINED-MODALITY THERAPY.** G. Blackledge & D. Crowther, *CRC Department of Medical Oncology, Christie Hospital, Manchester*

Thirty-two patients with gastrointestinal lymphoma have been treated at this hospital between 1976 and 1979, 12 with disease arising in the stomach, 16 with disease in the small intestine and 4 with large intestinal disease. Nine of the patients had nodular histologies, 12 had diffuse poorly differentiated lymphocytic or undifferentiated histology and 11 had diffuse histiocytic lymphoma (DHL). The patients were staged using the classification proposed by us (Blackledge *et al.*, 1979, *Clin. Oncol.*, **5**, 209). This has allowed a comparison of survival with a retrospective study of 104 patients, allowing for similar stage. Combination chemotherapy, vincristine, adriamycin and prednisone for DPDL and DHL, and cyclophosphamide, vincristine and prednisone for the nodular lymphomas, was given to patients with Stage II or greater disease (30/32), followed by radiotherapy to the abdomen whenever possible. Nineteen patients (53%) achieved a complete remission. Actuarial survival curves showed 51% alive at 3 years, with the deaths occurring within the first year. Improvements in survival were seen in Stage IIB/C, 90% 2-year survival compared with 30% in the retrospective study, and Stage III, 55% 2-year survival compared with 20% in the previous study ($P=0{\cdot}0079$). Patients with DHL had a survival of 63% at 2 years, with no deaths after 4 months. Patients with local problems, *e.g.* perforation or malabsorption, during treatment had median survival of 8 months, compared with 61% of patients without these problems being alive at 3 years. Combined-modality treatment with combination chemotherapy and radiotherapy represents an advance in the management of gastrointestinal lymphoma.

**EXTRAMEDULLARY PLASMACYTOMAS.** A. L. PAHOR*, J. A. H. WATERHOUSE† & J. POWELL†, **E.N.T. Department, Dudley Road and Sandwell D.G. Hospitals, Birmingham,* †*Regional Cancer Registry, Birmingham*

Extramedullary plasmacytomas (EMP) are uncommon tumours. Their diagnosis presents difficulty for both the clinician and the pathologist. They can be solitary or multiple. Thirty-three cases were diagnosed in the Birmingham Region between 1963 and 1976 (24 solitary and 9 multiple). The commonest site is the head and neck (24). Three cases have primary lymphonode involvement. This series is one of the largest reported in the literature so far and is population-based, thus reflecting the true incidence more than hospital-based series. Conditions which can be mistaken for EMP are chronic nonspecific infections such as plasma-cell polyp of the vocal cord; chronic specific infections such as yaws and sarcoidosis; monomorphic tumours such as neuroblastoma, reticulosarcoma and lymphomas. Investigations to exclude multiple myeloma are essential and should be carried out from the onset. A scoring system is designed to monitor the transformation of EMP to multiple myeloma and thus be a guide to chemotherapeutic treatment. Treatment of EMP is essentially radiotherapy. Life-long follow-up is mandatory in all cases, as local or distant recurrences can occur many years after apparently successful treatment. EMP should be considered as premyeloma stage and we urge the formation of a multidisciplinary group to study EMP, which hopefully will lead to more understanding of multiple myeloma.

**PRIMARY LYMPHOMAS OF THE TESTIS.** G. READ, *The Christie Hospital and Holt Radium Institute, Manchester*

Primary lymphoma of the testis is rare. During the period 1960–1979, 1307 patients with testicular tumours were registered at the Christie Hospital and Holt Radium Institute. Of these, only 51 patients were considered to have a primary lymphoma of the testis, the pathology being either the diffuse histiocytic, poorly differentiated lymphocytic or undifferentiated histological type. There were no cases of Hodgkin's disease of the testis. Only in those patients in whom the testicular involvement was the presenting symptom or the dominant feature was the testis accepted as the primary site. Eight cases had bilateral testicular involvement, and in 23 patients the disease was apparently confined to the testicle at presentation. The patients were treated in most instances by radiotherapy, though in later years chemotherapy was also used. The results of treatment are presented. Survival was poor. In the 28 patients with evidence of metastases at presentation, only 3 lived more than 12 months. However, the 5-year age-corrected survival in the patients with disease confined to the testicle, was 30%. Bone, skin and liver involvement was especially common. There is insufficient experience yet to determine whether chemotherapy has improved the outcome.

### THE PROGNOSTIC SIGNIFICANCE OF SERUM $\beta_2$-MICROGLOBULIN IN MULTIPLE MYELOMA.

D. Norfolk, J. A. Child, S. Kerruish, E. H. Cooper & A. M. Ward, *The Department of Haematology, The General Infirmary at Leeds, The Unit for Cancer Research, University of Leeds and The Supra Regional Protein Reference Laboratory, Sheffield*

The levels of serum $\beta_2$-microglobulin ($\beta_2$-m) were measured serially in 36 patients with multiple myeloma for 6 months to 5 years. The initial $\beta_2$-m levels are highly correlated to survival ($P=0{\cdot}01$). The median survival in months, according to the initial $\beta_2$-m levels (number of patients in parentheses) was as follows: $>6$ mg/l, 14 months (11); 3–6 mg/l, 19 months (12) and $<3$ mg/l, $>50$ months (13). These levels of $\beta_2$-m are correlated to tumour mass as calculated from multifactorial indices. An initial $\beta_2$-m of $>3$ mg/l tended to fall on treatment but rarely reached normal, a rising value usually heralded an irreversible progression, and when $>8$ mg/l, often accompanied by renal failure. The level of $<3$ mg/l was usually associated with a relatively benign evolution but carried an increased risk of infection.

These detailed studies have been compared to the pattern in 120 patients analysed by the Protein Reference Laboratory, and indicate they are representative of the disease in general. Creatinine $>140$ nM was only present in 3 of the patients in the longitudinal studies and in 10% of the Sheffield series. The paraprotein levels show a far wider variation than those of $\beta_2$-m.

### ALLOGENEIC MARROW TRANSPLANTATION IN ACUTE MYELOID LEUKAEMIA—AN UPDATE OF THE ROYAL MARSDEN HOSPITAL SERIES.

D. W. Hedley, *Royal Marsden Hospital Bone Marrow Transplant Team, Sutton, Surrey*

Although conventional drug treatment produces a remission rate of around 70%, virtually all patients with acute myeloid leukaemic (AML) relapse and ultimately die of their disease. More radical treatment while in remission with total body irradiation (TBI) and allogeneic marrow transplantation (BMT) from a histocompatible sibling donor now appears to offer the prospect of permanent cure (Thomas *et al.*, 1977, *Blood*, **49**, 511) and of 29 patients in first remission of AML treated in this manner at the Royal Marsden Hospital (RMH) only 3 have so far relapsed. Furthermore, the early part of the survival curve for these patients is no worse than that of a similar series of non-grafted patients (who did not have a matched sibling donor), suggesting that TBI and BMT are not associated with an excess of early, treatment-related deaths.

The use of the new immunosuppressive agent Cyclosporin A has reduced the incidence of fatal GVH disease from 11/26 to 1/25 in the RMH series, and we are now exploring the use of partially mis-matched donors. TBI and BMT should be considered for patients with AML in remission who have histocompatible siblings.

### THE ROLE OF TRANSTRACHEAL ASPIRATION IN THE DIAGNOSIS OF RESPIRATORY INFECTION IN NEUTROPENIC PATIENTS WITH ACUTE LEUKAEMIA.

M. L. Slevin, R. Bell, A. G. Catto-Smith, E. J. Shaw, J. M. Ford, J. S. Malpas & T. A. Lister, *ICRF Department of Medical Oncology and Department of Bacteriology, St Bartholomew's Hospital, London*

Transtracheal aspiration (TTA) was performed during 35 febrile episodes in 31 neutropenic patients with acute leukaemic and clinical or radiological evidence of respiratory-tract infection. Twenty-five patients were receiving antibiotics at the time of TTA (Tobramycin and Cephazolin 14, Tobramycin and Flucloxacillin 5, other combinations 6). A 14-gauge Davol IVI catheter was inserted under sterile conditions through the crico-thyroid membrane. Bronchial secretions were aspirated, with or without lavage with 10 ml of normal saline, and specimens were cultured immediately. The procedure was carried out under platelet cover if indicated, and no serious haemorrhagic complications were seen. Minor surgical emphysema occurred in one patient. There was no radiological evidence of pneumothorax. Positive cultures were obtained from TTAs (46%). In these 16 cases, concurrent sputum examination was positive to culture in only 4, negative in 5, and unobtainable in 7. There was no case in which the TTA was negative and the sputum positive. The TTA was positive in 16/30 cases in which the chest radiograph was abnormal. 12 cases in whom the TTA was positive were receiving broad-spectrum antibiotics and in 8 of these the regimen was altered. In the 4 positive cases not an antibiotics, the findings influenced the choice of drugs. The high diagnostic yield of this simple test suggests that its routine use in the diagnosis of chest infection in neutropenic patients should be further evaluated.

### NEUTROPENIA AND INFECTION IN ACUTE MYELOGENOUS LEUKAEMIA. A COMPARISON OF THREE CHEMOTHERAPEUTIC REGIMENS.

A. Rohatiner, R. Bell, J. Ford, J. S. Malpas & T. A. Lister, *ICRF Department of Medical Oncology, St Bartholomew's Hospital, London*

The relationship of fatal infection to neutropenia during remission induction for AML is analysed. 150 consecutive patients received repeated cycles of Adriamycin (ADR) Cytosine Arabinoside (Ara-C), Vincristine and Prednisolone given over 9 days (Regimen 1), or ADR, Ara-C and Thioguanine given over 5 days, at either high (Regimen 2) or very high doses (Regimen 3). The table shows the number of pts and fatal infections and the mean duration of neutropenia in days, associated with the first 2 treatment cycles, when all the infective deaths occurred.

| Regimen | Pts | Fatal infections | Neutrophils × $10^9$/l | | |
|---|---|---|---|---|---|
| | | | <0·1 | 0·1–0·5 | 0·5–1·0 |
| 1 | 82 | 19 | 23 | 12 | 7 |
| 2 | 27 | 2 | 19 | 10 | 5 |
| 3 | 41 | 13 | 19 | 8 | 5·4 |

The duration of neutropenia was longest in Regimen 1 (possibly because treatment was given over 9 days) and was virtually identical in Regimens 2 and 3. However, there was a significantly higher incidence of fatal infection in the more intensively treated group (Regimen 2 *vs* 3, $P<0{\cdot}05$). 22/34 fatal infections were preceded by profound neutropenia (neutrophils $<0{\cdot}1\times 10q$/l) for a mean of 8 days. 27 of these 34 pts showed no rise in neutrophil count in response to infection. The data confirm that fatal infections are associated with persistant severe neutropenia. Intensification of therapy, resulting in a significant increase in fatal infections, did not increase the degree or duration of neutropenia, suggesting that other factors, such as mucosal ulceration significantly contribute to outcome.

### CYTOGENETIC EVIDENCE FOR A NEW CLONE AT RELAPSE OF ALL: A CASE REPORT.

L. M. Secker-Walker & C. A. Sieff, *Dept of Cytogenetics and Immunology, Royal Marsden Hospital, Fulham Road, and Dept of Haematology, Hospital for Sick Children, Great Ormond Street*

Two possible mechanisms of disease relapse in acute lymphoblastic leukaemia (ALL) have been suggested. Cells of the malignant clone, harboured since diagnosis, may re-emerge; alternatively leukaemia may occur in a previously unaffected cell-line. A case is reported in which chromosomal evidence, from each of three disease episodes, indicates the working of both mechanisms at different times in the same patient.

A boy aged 7 years when ALL was diagnosed, had a first marrow relapse 4 years later. This was followed by a good second remission of 18 months. The patient died in second relapse 6 years after diganosis. On marrow morphology and cytochemistry the cells in the 3 disease episodes were indistinguishable. The chromosome constitution of all marrow cells at diagnosis was 47 XX, del(5)(q13)del(15)(q15)+Mar. However, both relapses were associated with a new chromosomally abnormal clone characterized by a long-arm deletion of chromosome 6, with none of the abnormalities seen at diagnosis. The use of chromosomal abnormalities to identify a malignant clone is considered in the light of tumour-evolution theory. The influence of therapy on the suppression, eradication and/or initiation of a chromosomally abnormal malignant clone is discussed.

**THERAPEUTIC DRUG MONITORING OF METHOTREXATE.** J. F. B. STUART, J. G. McVIE & K. C. CALMAN, *Department of Clinical Oncology, and* J. R. LAWRENCE & W. H. STEELE, *Department of Materia Medica, University of Glasgow*

High-dose MTX in the treatment of osteogenic sarcomas requires monitoring of MTX throughout the infusion and up to at least 48 h afterwards, in order to establish the amount of folinic acid required to avoid toxicity. Enzyme immunological techniques in the form of the "EMIT" system (Syva) provide a rapid method of analysis. Comparability with other methods of analysis is important to establish the serum concentrations of the MTX at which folinic acid is administered. Preliminary results in 30 samples show that readings using selenium-based radioimmunoassay (RIA) (Paxton *et al.*, 1978, *Clin. Chem.*, **24,** 1534) were about 67% of results using the EMIT system. This difference between the assays was found over the whole concentration range: RIA 66% at 0–1000 nM; 62% at 1000–5000 nM and 73% in samples containing >5000 nM. Overall there was good correlation between the methods ($r=0{\cdot}84$, $P<0{\cdot}001$).

**METABOLISM AND EXCRETION OF METHOTREXATE (MTX) IN MAN.** A. L. STEWART, J. MARGISON & P. M. WILKINSON, *Department of Clinical Pharmacology, Christie Hospital, Manchester*

Previous kinetic studies of MTX have relied on drug determination principally by $^3$H-MTX which is now known to present certain technical difficulties and makes identification of metabolites difficult. We have developed an unambiguous HPLC assay for both MTX and 7,OH-MTX and compared concentrations of both in bile, serum and urine to those obtained by radioimmunoassay (RIA). Two patients with choledocal T-tubes received MTX 100 mg/m$^2$ i.v.; both patients had disseminated malignancy, but renal and hepatic function were normal by conventional biochemical criteria. The decay of drug in serum was triphasic, and concentrations were similar for both assays, with the exception of the terminal decay phase. For urine, although RIA in these 2 patients gave similar concentrations to HPLC, it has consistently overestimated drug excretion in a larger patient series, due to interference from 2·4-diamino N$^{10}$-methylpteroic acid. It was not possible to determine MTX accurately in bile by RIA because of marked quenching. It was, however, readily identifiable by HPLC, peak concentrations being reached 0·5–3 h after drug administration. Decay of drug in bile was biphasic, with a mean terminal half-life of 15 h. The biliary concentration always exceeded that in serum, with a maximum bile:serum ratio of 100:1. Cumulative biliary excretion could only be determined in one patient, where 13% of the administered dose was excreted in 48 h. Although moderate-dose MTX is said not to be metabolized, 7,OH-MTX was identified in all 3 constituents. Only small amounts were present in bile in contrast to serum where substantial amounts may be present 24 h after administration. This study confirms that the renal route is the main excretory pathway for MTX, and on the basis of these observations dose reduction is probably not necessary in the presence of obstructive jaundice.

**PHARMACOKINETICS OF CYCLOPHOSPHAMIDE IN PATIENTS WITH OAT-CELL CARCINOMA OF THE BRONCHUS.** P. M. WILKINSON, N. THATCHER, S. B. LUCAS & S. CUSHING, *Dept Clinical Pharmacology and CRC Dept of Medical Oncology, Christie Hospital, Manchester*

Serial serum and urine concentrations of cyclophosphamide (CY) and alkylating metabolites (AM) were determined in 8 patients with histologically proven oat-cell carcinoma of the bronchus following i.v. doses of 1·5, 2·5 and 3·5 g/m$^2$ respectively. For each dose the serum decay was triphasic, the mean terminal half-life of CY being 5·8, 8·6 and 8·8 h and of AM 14, 14·7 and 12·6 h. Although considerable variation between patients was observed in the AM/CY ratio, this did not increase with successive dose increments. The area under the concentration/time curve from 6–24 h for CY could be directly correlated with the percentage fall in neutrophil count, but for the AM this applied to the terminal phase only. No significant difference was found between the change in neutrophil count and response but there was one between ratios and response. We conclude that incremental dose increase does not impair hepatic biotransformation of CY, and that the AM/CY ratio may be an important factor in predicting tumour response.

**BLEOMYCIN DRUG MONITORING IN THE MANAGEMENT OF MALIGNANT EFFUSIONS.** J. F. B. Stuart, J. M. Trotter, J. G. McVie & K. C. Calman, *Department of Clinical Oncology, University of Glasgow, and* W. Aherne & S. James, *Department of Biochemistry, University of Surrey*

Intracavitary bleomycin (BLM) has now been used in a total of 35 patients with malignant effusions, with an overall response rate of 70%. There were 6 possible drug-related deaths with this therapy; apart from this it was well tolerated, with low toxicity. Trotter *et al.* (1979, *Br. J. Cancer*, **40,** 310) have shown that high serum levels of BLM in one of the treatment-related deaths suggests that intracavitary doses of BLM in excess of 40 mg/m$^2$ should be avoided in the elderly.

These findings led to BLM monitoring in all patients given the drug by the intrapleural or i.p. routes. Systemic peak concentrations of BLM showed a 5- to 10-fold increase in some cases, and one of the patients who died had normal plasma concentrations of BLM during a previous infusion with the same dose schedules. In a further 5 patients studied who had low toxicity, the systemic BLM concentration was 40% of that when equivalent amounts were given by the i.v. route (Stuart *et al.*, 1979, *Br. J. Cancer*, **40,** 316).

In a further 4 patients a predictive dose of 5 mg BLM was given, followed by the appropriate higher dose 4–5 h later. The mechanism of BLM toxicity associated with these levels of intracavitary drug is unclear, but it seems reasonable to continue to monitor BLM during intracavitary therapy.

**ESTIMATION OF EXPOSURE TO ALKYLATING AGENTS USING GAS CHROMATOGRAPHY - MASS SPECTROMETRY.** P. B. Farmer & E. Bailey, *MRC Toxicology Unit, Carshalton, Surrey*

Alkylating agents form an important class of environmental carcinogens, in addition to being widely used as anti-cancer agents. *In vivo* exposure to alkylating agents leads to the formation of S-alkylcysteine and N-alkylhistidine derivatives in haemoglobin. Methods have been developed for the quantitation of these amino acids in rat or human haemoglobin, using capillary gas chromatography–chemical ionization–mass spectrometry. After treatment of rats with the methylating agent methyl methanesulphonate (MSS) the amount of S-methylcysteine in haemoglobin bears a linear relationship with the dose of MMS injected. In contrast, the degree of methylation after injection of the anti-cancer drug 5-(3,3-dimethyl-1-triazene)-imidazole-4-carboxamide (DTIC) is very low. Hydroxyethylhistidine has been determined in the haemoglobin (after sodium borohydride reduction) of humans exposed to vinyl chloride.

**BIOGENESIS OF BLADDER CANCER IN THE FISCHER 344 RAT BY INTRAGASTRIC N-BUTYL-N-(4-HYDROXYBUTYL) NITROSAMINE (BBN).** R. M. Hicks, J. Chowaniec, E. D. Massey & A. Harvey, *School of Pathology, Middlesex Hosp. Med. School, London*

The biological response of animals to BBN is affected by species (Hirose *et al.*, 1976, *Gann*, **67,** 175), strain (Moon, unpublished) and sex (Bertram & Craig, 1972, *Eur. J. Cancer*, **8,** 587). It is an organ-specific bladder carcinogen for the rat, and we also find it to be a powerful bladder carcinogen for the hamster but ineffective in the baboon (Hicks *et al.*, unpublished). In the Fischer 344 rat, BBN produces multi-focal, large, exophytic, well-differentiated, transitional-cell tumours with some squamous metaplasia, which generally do not metastasize but slowly invade the bladder wall. Their histopathology and ultrastructure are comparable to low-grade, superficial, papillary bladder cancers in man which are often regarded as relatively benign. Nevertheless the survival of these animals is related to the BBN dose and the tumours cause death by obstruction and haemorrhage. The dose-response to BBN is measured in terms of total tumour volume produced with time and is reffected by the life tables. The BBN/Fischer rat system has been used in many laboratories to induce bladder tumours for chemotherapeutic trials and other investigations. Its suitability as a model for human bladder cancer is limited to the local disease only, and it cannot be used to study metastasis.

**SILICA-INDUCED LYMPHOMA IN RATS.** M. M. FENNELL WAGNER & J. C. WAGNER, *MRC Pneumoconiosis Unit, Llandough Hospital, Penarth, S. Glamorgan*

Following upon a single i.p. injection of crystalline silica (quartz) into the pleural cavity of Wistar-derived rats at 5–6 weeks of age, malignant lymphomas develop (Wagner, J. C., 1962, *Nature*, **196,** 180). They have been designated malignant lymphoma of histiocytic type (Wagner, 1976, *J. Natl Cancer Inst.*, **57,** 509) and the rate is ~40%. In 2 other strains of rats the rate was 5% and 7% (Wagner, submitted for publication) and it also varied with the type of silica injected (Wagner, submitted for publication). The tumours were not found after injection with saline, coal or carbon (Wagner, 1976). Lymphocytic lymphosarcoma occurred equally in saline- and silica-injected rats. Electron microscopy and role of thymectomy are discussed, and the importance of the production of a lymphoma consequent upon the introduction of an agent believed to be specifically cytotoxic to macrophages (Allison, 1976, *In Vitro Methods in Cell-Mediated Tumour Immunity*, p. 395). Although numerous experiments have been performed with quartz during the last 50 years, this is the only study in which this mineral has been shown to be associated with malignancy.

**DETECTION OF BBN - INDUCED BLADDER TUMOURS BY MEASUREMENT OF HAEMATURIA AND URINE CYTOLOGY.** J. TURTON, C. HAWKEY*, J. GWYNNE, L. SIMON & R. M. HICKS, *School of Pathology, Middlesex Hospital Medical School, and *Nuffield Labs. of Comp. Med., Regent's Park, London*

The development of bladder tumours in the urinary bladder of the F344 rat in response to BBN is dose-related. Thus 12 months after dosing with 300, 600 or 1200 mg BBN the total tumour volumes were 7,380 and 2200 $mm^3$ respectively. To avoid killing animals in order to detect the tumours histologically, the possibility was investigated of measuring urine parameters as indicators of tumour growth. Haematuria was found to parallel tumour growth. The correlation between tumour size and haematuria was demonstrated for individual animals after different doses of BBN and the sensitivity of the method discussed. In general, the method is reliable for the detection of tumours over 40 $mm^3$ in size, but a number of false negatives were obtained with very small tumours. Haemorrhagic anaemia was found to be associated with the presence of large bladder tumours. Examination of urine sediments of 62 animals by urine cytology gave an 80% correct correlation with bladder pathology as found at subsequent necropsy. The 6 false negatives were not correlated with tumour size but all had heavy fungal contamination preventing accurate diagnosis. Thus while neither technique alone is entirely reliable, we have found that together they provide a useful way of monitoring the progress of animals in bladder carcinogenesis trials.

**CHARACTERISTICS OF N-NITROSO-METHYLUREA-INDUCED MAMMARY TUMOURS IN THE RAT.** J. C. WILLIAMS, R. C. COOMBES & B. A. GUSTERSON. (Introduced by G. C. Easty). *Ludwig Institute for Cancer Research, Sutton, Surrey*

N-nitrosomethylurea (NMU) has been reported to induce mammary tumours in rats, with a high incidence of metastasis to marrow and spleen and the development of hypercalcaemia (Gullino *et al.*, 1975, *J. Natl Cancer Inst.*, **54,** 401). In our hands i.v. NMU to 50–55-day-old rats induced mammary tumours in 78% of F344/N and 91% of ICRF Wistar females. Mean latent period for F344/N rats was 149 days and for ICRF Wistar rats was 93 days. 98% of mammary tumours were classified histologically as adenocarcinomas. Primary tumours of non-mammary origin were detected at low incidence. These were squamous-cell carcinomas in the peri-urethral and auditory regions, adenocarcinomas of the bronchus, endometrium and colon, primitive neural ectodermal tumours of the brain and disseminated lymphoma/leukaemia. No histological evidence was found for metastases of either the mammary tumours or other primary tumours in lungs, femora, vertebrae, liver, kidneys, spleen, brain or adrenals, and plasma calcium measurements varied within the normal range independently of tumour volume. No histological difference was found between NMU- and DMBA-induced mammary adenocarcinomas. Oophorectomy at the time of NMU administration greatly inhibited tumour induction; oophorectomy when at least one tumour per animal was palpable produced growth delay or regression in all cases, with subsequent tumour re-growth in 7/8 animals. All NMU- and DMBA-induced mammary tumours tested contained cytoplasmic oestrogen receptor at similar concentrations. No evidence for tumour-induced hypercalcaemia was found. In view of the inability of these tumours to metastasize and/or cause hypercalcaemia the NMU model of breast cancer offers little advantage over other hormonally responsive models.

**REACTIVE HYPERPLASIA AFTER COLOSTOMY CLOSURE ENHANCES CHEMICAL CARCINOGENESIS IN THE DISTAL COLON.** R. C. N. WILLIAMSON, O. T. TERPSTRA, E. P. DAHL & R. A. MALT, *Massachusetts General Hospital, Boston, U.S.A., and University Department of Surgery, Bristol Royal Infirmary*

Adaptive hyperplasia may explain enhanced colonic carcinogenesis in rats after small-bowel resection (Williamson *et al.*, 1978, *Cancer Res.*, **38**, 3212) or pancreatobiliary diversion (Williamson *et al.*, 1979, *Gastroenterology*, **76**, 1386). Using male Fischer rats (n = 205) susceptibility to cancer was studied in colon re-exposed to the faecal stream 4 weeks after defunctioning transverse colostomy. Other groups had laparotomy alone, permanent colostomy, or colonic transection repeated after 4 weeks. Tumours were induced by 1,2-diemthylhydrazine (300 mg/kg) in divided s.c. doses over 11 weeks, starting 2 days after the second operation.

In distal colon, amounts of RNA, DNA and protein fell by 53–58% 4 weeks after transverse colostomy ($P < 0{\cdot}001$), but returned to normal within 1 week of colostomy closure. This reactive hyperplasia promoted the development of distal tumours, as compared with repeated colonic transection (incidence 32% *vs* 6%: $P < 0{\cdot}03$). In proximal colon, protein and nucleic acid contents were unaltered by transverse colostomy, but were increased by 18–59% 4 weeks after restoration of intestinal continuity ($P = 0{\cdot}05$–$0{\cdot}002$); tumour yields were unchanged, however. Suture-line tumours were commoner after repeated transection than after colostomy closure (76% *vs* 39%: $P < 0{\cdot}01$). The presence of a permanent colostomy did not affect the number of distal tumours, but 26% of these animals developed cancers at the stoma.

Hypoplasia is confined to the distal colon after transverse colostomy, but it occurs in both proximal and distal colon after colostomy closure. Intense adaptive hyperplasia promotes carcinogenesis in the distal colon.

**DETECTION OF HERPESVIRUS-CODED MATERIAL IN NEOPLASTIC CERVICAL TISSUE.** R. P. EGLIN, A. B. MACLEAN, F. SHARP, J. C. M. MACNAB, J. B. CLEMENTS & N. M. WILKIE, *Departments of Midwifery and Virology, University of Glasgow*

The presence of virus-specific RNA has been investigated by the technique of *in situ* hybridization using 7·5μm sections of human normal and neoplastic cervical tissue. The $^{125}$I-labelled DNA probes used were from herpes simplex virus 2 ($HSV_2$), adenovirus types 2 and 5, and bacteriophage λ. Results were assessed by counting grains within a standard area. A positive result was considered to be a value at least twice the grain count for control sections, in at least 2 of the 4 consecutive serial sections on each slide.

No positive hybridization was found for the adenovirus and bacteriophage probes against neoplastic and benign cervical tissue. But $HSV_2$ probe produced positive hybridization for all 11 biopsies from cervical intraepithelial neoplasia, for 14/17 squamous-cell cervical carcinomas, for 1/9 cervical adenocarcinomas, and 1/10 biopsy samples of benign epithelium. Surface swabs of the cervix taken from the one case of adenocarcinoma positive for hybridization were positive for free virus; all other swabs were negative.

**AN INCREASED METHIONINE REQUIREMENT BY TUMOUR CELLS *IN VITRO*.** M. J. TISDALE, *Department of Biochemistry, St Thomas's Hospital Medical School, London*

The ability of a number of normal and neoplastic cell lines to survive and grow in culture medium in which methionine was replaced by its immediate precursor homocysteine has been studied. None of the tumour lines showed optimal growth under such nutritional conditions, although there was a variability in the ability to survive and grow. In contrast the normal cell lines studied showed optimal growth in media containing homocysteine only. Although homocysteine alone was unable to support growth of tumour cells, it did stimulate growth under conditions in which methionine became growth-limiting. A correlation has been obtained between the ability of a cell line to survive in media in which homocysteine substitutes for methionine and the minimal concentration of methionine required for optimal growth. Thus normal cell lines grow optimally in media containing only 0·5 $\mu$g/ml of methionine in the presence of homocysteine, whereas some of the tumour cell lines require a concentration of methionine greater than 2 $\mu$g/ml. This suggests that the inability of tumour cell lines to survive in methionine-depleted, homocysteine-supplemented media is not due to any intrinsic biochemical defect, but to the higher methionine requirement of such cells.

**CLUSTERING OF STOMACH CANCER IN ARGYLL, 1971–78.** J. McHardy, C. R. Gillis & D. H. Hole, *West of Scotland Cancer Surveillance Unit, Ruchill Hospital, Glasgow*

The establishment of a relationship between alimentary cancer in cattle and various putative carcinogens (*e.g.* bracken, viruses) and anecdotal evidence of alimentary cancer in both humans and animals living in close proximity, suggested an investigation with implications for human cancer. Consequently a population-based study of the geographical distribution of human cancer was carried out in Argyll in S.W. Scotland where the occurrence of bracken is high, and compared with the distribution of alimentary cancer in Ayrshire, where bracken is sparse.

Previous studies of human cancer in Ayrshire have suggested space/time clustering of alimentary cancer, and this present study has revealed a cluster in space and time of stomach cancer which was most marked in the year of maximum incidence, 1974. This cluster however did not occur in the same areas as the alimentary cancer in cattle. Preliminary studies have been carried out using existing data of occupation and family background, trace elements in the soil and nitrates in the water supply, without yielding definitive results at this stage.

**AN INCREASE IN THE INCIDENCE OF BLADDER CANCER.** C. R. Gillis*, W. M. MacIntyre*, J. M. Glennie*, P. Boyle*, A. T. Sandison† & A. J. Malcolm†, **West of Scotland Cancer Surveillance Unit, Ruchill Hospital and* †*University Dept of Pathology, Western Infirmary, Glasgow*

Clinical study of 667 cases of bladder cancer seen between 1957 and 1972, together with an epidemiological study of bladder cancer in the County of Ayrshire, Scotland, between 1958 and 1977, revealed a 3-fold increase in incidence. This increase, proportionately similar in males and females, is not due to changes in the structure of the population, and an increase of this magnitude has not been reported elsewhere. The cases were classified at the outset of this clinical study by occupational status into "high" and "low" risk groups. The high-risk groups consisted of chemical workers, miners, farm and agricultural workers and rubber workers. While there was an increase in incidence in the high-risk group, the most striking increase occurred in the general population group, implying the existence of some as yet unrecognized aetiological factor. The sites within the bladder at which these tumours occurred were similar to published series; however the spectrum of tumour types differed from other British series in having a lower representation of transitional-cell tumours. Whilst these differences may arise from variations in the evaluation of metaplastic change, they may represent a true variation in bladder-cancer histology.

**CANCER OF THE LARYNX IN SCOTLAND.** P. Boyle*, A. G. Robertson†, C. R. Gillis*, H. Yosef† & G. E. Flatman†, **West of Scotland Cancer Surveillance Unit, Ruchill Hospital, and* †*Institute of Radiotherapeutics and Oncology, Western Infirmary, Glasgow*

Evidence from epidemiological, experimental and necropsy studies suggests that smoking cigarettes, cigars or pipes is a significant causative factor in the development of cancer of the larynx. A strong dose–response relationship with the number of cigarettes smoked is a particular feature of the disease. In Scotland the proportion of the population smoking remains high, and the average annual amount of tobacco smoked is increasing. Thus an increase in mortality from laryngeal cancer would be expected. However, in Scotland there has been no increase in mortality from laryngeal cancer, and neither an increase in incidence in a well studied area of the West of Scotland with high-quality registration data, nor a detectable improvement in survival to account for this anomaly. Treatment and staging details were available for patients treated in the Glasgow Institute of Radiotherapeutics and Oncology between 1968 and 1978, and the survival was typical of clinical and registry series over the same period.

**CHEMOTHERAPY OF ADVANCED MALIGNANT TERATOMAS.** E. S. NEWLANDS, R. H. BEGENT, S. B. KAYE, G. J. RUSTIN & K. D. BAGSHAWE, *Charing Cross Hospital, London*

Between 1977 and November 1979 we have treated 53 patients with malignant teratomas (43 males, 10 females). Thirty (70%) out of 43 male patients had advanced and bulky disease at the time of presentation. Treatment schedules were:

(A) Day 1: Vincristine 1·0 mg/m$^2$; Methotrexate 300 mg/m$^2$. Day 2: Bleomycin 30 mg as an i.v. infusion for 48 h; and folinic acid 15 mg 12-hourly for 4 doses starting at 24 h. Day 4: Cis-Platinum 120 mg/m$^2$ with mannitol diuresis.

(B) VP.16.213 100 mg/m$^2$ Days 1–5; Actinomycin-D 0·5 mg Days 3–5; Cyclophosphamide 500 mg/m$^2$ Day 5.

(C) Hydroxyurea 500 mg q.d.s. Days 1 and 2; Vinblastine 5 mg/m$^2$ Day 3; Chlorambucil 10 mg b.d. Days 3–5.

(D) The same as Treatment A without the Cis-Platinum.

Schedules were repeated in the following sequence: A, A, B, C, D. Courses were continued in sequence B, C, D, unless drug resistance developed (detected by monitoring human chorionic gonadotrophin and α-fetoprotein) and the inappropriate treatment schedule was omitted. Of the initial 33 male patients, 22 are off treatment (mean 9·5 months) with 19 compete responses and 3 with static C.T. Scan nodules. Life-table analysis projects a survival of 66%. Nine out of 10 ovarian-teratoma patients are alive (1 had active disease). Toxicity was relatively mild and febrile episodes with neutropenia occurred in 24% of 53 patients. Adverse prognostic factors at the time of entry were recognized in 10 of the 11 patients who have died.

**TUMOUR-MARKER LEVELS AND PROGNOSIS IN MALIGNANT TERATOMA OF THE TESTIS.** R. H. J. BEGENT, J. R. GERMA-LLUCH & K. D. BAGSHAWE, *Department of Medical Oncology, Charing Cross Hospital, London*

The effect of 6 putative prognostic factors on survival was studied in patients with Stage III and IV malignant teratoma of the testis. Differences between survival curves were tested for statistical significance. A diameter of $>5$ cm in the largest tumour mass and $>8$ pulmonary metastases were adverse prognostic factors ($P$ 0·016 and 0·029 respectively). Patients with malignant teratoma trophoblastic fared worse than those with malignant teratoma undifferentiated ($P$ 0·024) but there was no significant difference between malignant teratoma intermediate and either of the other two groups. Previous chemotherapy or radiotherapy had no significant effect.

Serum α-fetoprotein (AFP) $>10^3$ μg/l and serum β subunit of human chorionic gonadotrophin (hCG) $>10^5$ i.u./l were found to predict a poor prognosis ($P$ 0·001 and 0·019 respectively). A combination of measurements of the tumour markers gave the most consistent indication of prognosis, in that patients with either AFP $>10^3$ μg/l or hCG $>10^5$ i.u./l, or both, fared very badly compared with those with neither factor ($P$ 0·001).

Serum concentrations of AFP and hCG should be stated in reports of treatment of testicular teratoma, in order to provide a basis for comparison with other series. Regular and frequent measurements of these markers are appropriate throughout the whole period of clinical management of patients with malignant teratoma.

**TRIAZINATE IN COMBINATION CHEMOTHERAPY OF ADVANCED COLORECTAL CANCER.** M. T. Shaw, *Department of Clinical Oncology, University of Newcastle upon Tyne* (Introduced by J. Dickson)

Baker's antifol (BAF), a triazine antifol, is a potent inhibitor of dihydrofolate reductase, and has shown activity against adenocarcinoma of the colon in man (1977, *Cancer*, **40,** 9; *Cancer Treat. Rep.*, **62,** 553). In an attempt to improve poor response rates obtained in this disease with 5-fluorouracil (FU) alone, 34 fully evaluable and previously untreated patients were treated with a combination consisting of methyl CCNU 60 mg/m$^2$ p.o. Days 1 and 2, FU 400 mg/m$^2$ i.v./day for 5 days and BAF 150 mg/m$^2$ by i.v. infusion over 1 h each week. Cycles were repeated every 6 weeks. There were 6 complete responses and 4 partial responses, for a total response rate of 18%. However, 7 more patients showed improvement. The median duration of response was 29 weeks and the median survival for all responders and nonresponders was 34 weeks. Toxicity consisted of moderate neutropenia and thrombocytopenia, occasional skin rashes and mild oral mucositis. Vomiting occurred in only one patient. There seems to be no improvement in response rate or survival with the use of this combination over those produced by FU alone.

**SERUM $\alpha_1$-ANTICHYMOTRYPSIN AND CEA LEVELS IN UPPER-GASTROINTESTINAL-TRACT CANCER.** S. Rashid, E. H. Cooper, G. Eaves, J. O'Quigley & A. T. R. Axon, *Unit for Cancer Research, University of Leeds and Gastroenterology Unit, General Infirmary at Leeds*

In 95 patients with stomach cancer the combination of the levels of the pre-operative levels of serum $\alpha_1$-antichymotrypsin (ACT) and CEA were found to be of use in prognosis. The median survival in weeks according to levels of ACT and CEA (numbers in parentheses) were as follows: ACT $<0{\cdot}8$ g/l, CEA normal, 45 weeks (37); ACT $>0{\cdot}8$ g/l, CEA normal, 10 weeks (55); ACT $>0{\cdot}8$ g/l, CEA raised ($>10$ ng/ml, Pharmacia assay), 6 weeks (26).

This system appears to have potential for predicting the findings at laparotomy, and could help in clinical decision making and the stratification of patients in postoperative trials of chemotherapy.

These parameters were also found to be powerful discriminants in the separation of 37 cases of pancreatic cancer from 36 cases of chronic pancreatitis. However, the serum changes in cases of obstructive jaundice due to gall stones were such that the combination of ACT, CEA and routine liver-function tests could not provide a discriminant function to separate these three groups of diseases.

**ALBUMIN METABOLISM AND DISTRIBUTION IN CACHECTIC CANCER PATIENTS.** J. M. Trotter, K. C. Calman, S. Gordon, G. Raines, J. Bell & A. Fleck, *Departments of Clinical Oncology and Pathological Biochemistry, University of Glasgow*

Albumin catabolism is reduced in protein-calorie malnutrition but has been reported high in patients with cancer. Albumin metabolism and distribution was studied in a group of cachectic cancer patients with serum albumin concentrations $<30$ g/l to investigate whether differences in albumin metabolism and the acute-phase response could be detected between patients with anorexia secondary to disseminated malignancy and those with upper gastrointestinal lesions causing reduced dietary intake. Undenatured albumin was specially prepared and labelled with $^{131}$I (McFarlane, 1958, *Nature*, **182,** 53). Plasma and urinary $^{131}$I was measured to monitor the distribution and rate of excretion of the radioactive catabolic products. Together with the $^{131}$I-HSA faecal and urinary losses, the time course of disappearance of $^{131}$I-HSA from the plasma was followed until the terminal exponential was established. The fractional catabolic rate (FCR), transcapillary escape rate (TCER) and the distribution of albumin (extravascular to intravascular ratio (E/P) were determined by compartmental analysis (Matthews, 1957, *Phys. Med. Biol.*, **2,** 36). Six cachectic cancer patients with $>10\%$ weight loss and no cytotoxic chemotherapy in the preceding month had an elevated FCR, TCER and E/P ratio with an associated acute-phase response. Two patients with reduced food intake for mechanical reasons demonstrated a low FCR consistent with protein–calorie malnutrition, and had only a mild acute-phase response. In addition, the survival of these two patients was greater than 5 of the patients with high FCR. The remaining patient responded to a change in therapy. We conclude that cancer patients with serum albumin <30 g/l who have a high FCR and associated acute-phase response have a poor prognosis if untreated.

**SERUM GLYCOPROTEIN HORMONE α-SUBUNIT LEVELS, HORMONE RECEPTORS AND DISEASE STAGING IN BREAST CANCER PATIENTS.** I. A. MacFarlane*, D. Barnes*, J. M. T. Howat†, P. Durning†, C. G. Beardwell* & H. Bush‡, **Department Endocrinology, †Department of Surgery and ‡CRC Department of Medical Oncology, Christie Hospital, Manchester*

Serum glycoprotein hormone α-subunit was measured in 162 consecutive, primary breast-cancer patients (56 premenopausal), 59 patients with benign breast lumps (46 premenopausal) and 112 controls (56 premenopausal). Serum α-subunit levels in the premenopausal cancer patients were significantly higher than in the controls ($P$ 0·037; Mann–Whitney U Test). There was no difference in α level between the premenopausal benign patients, the premenopausal cancer patients and the controls ($P > 0{\cdot}02$). There was no difference in α level between the 3 postmenopausal groups (cancer, benign and controls) ($P > 0{\cdot}18$).

In the premenopausal cancer patients, increasing disease stage (TNM clinical staging with histological confirmation of nodal status) was associated with increasing α-subunit level ($r$ 0·47, $P = 0{\cdot}0001$; Spearman's rank correlation). However, in postmenopausal patients there was no correlation between α level and disease stage ($r$ 0·1, $P > 0{\cdot}3$).

The tumour cytosol oestrogen and progesterone receptor status was estimated in 40 premenopausal and 91 postmenopausal patients. In 27 of these premenopausal and 41 postmenopausal patients the tumour nuclear oestrogen-receptor status was also estimated. No association was found between α level and the presence of the individual receptors or combinations of these receptors.

In view of the correlation of α-subunit with disease stage, increased α level in premenopausal patients might indicate a worse prognosis, as in premenopausal metastatic-melanoma patients (MacFarlane *et al.*, 1979, *Eur. J. Cancer*, **15**, 1497).

**PLASMA AMINO-ACID PROFILES IN CANCER PATIENTS WITH WEIGHT LOSS.** J. M. Trotter, J. E. Carlyle, T. Habeshaw, F. R. Macbeth & K. C. Calman, *Departments of Clinical Oncology and Biochemistry, Gartnavel General Hospital, Glasgow*

Fasting plasma amino-acid patterns in protein-calorie malnutrition have been extensively studied, but there is little comparable data in the cancer patient with concomitant weight loss. Fasting amino-acid patterns were determined in a group of 39 anorectic cancer patients with weight loss, which exceeded 10% in the majority. None had received chemotherapy or radiotherapy within the preceding 3 weeks. Samples were obtained between 08:30 and 09:30 and analysed by ion-exchange chromatography on a Locarte amino-acid analyser (Spackman *et al.*, 1958, *Anal. Chem.*, **30**, 1185). Comparable sampling was performed in patients admitted to hospital for elective eye surgery, none of whom were known to have systemic illness, malignancy, or weight loss. Significantly lower mean values for threonine, alanine, ornithine, histidine, arginine, citrulline, cystine and methionine were found in the cancer patients, together with elevation in the mean value of glutamic acid. Unlike pure protein-calorie malnutrition in which levels of branch-chain amino acids are low, the mean concentrations of leucine and isoleucine were comparable with controls but with twice the range of values. It is suggested that high concentrations of valine, leucine and isoleucine may reflect early protein deprivation and muscle catabolism, whilst low levels reflect more established cancer cachexia with greater loss of lean body mass. In addition the ranges of aspartic acid, asparagine, glutamine, glycine, phenylalanine and tyrosine values were markedly greater in the patient group.

**CAE ESTIMATION IN CEREBROSPINAL FLUID IN PATIENTS WITH METASTATIC BREAST CANCER.** D. P. Dearnaley*†, S. Patel†, T. J. Powles* & R. C. Coombes*†, **Department of Medicine, The Royal Marsden Hospital and †Ludwig Institute for Cancer Research, Sutton, Surrey.* (Introduced by G. C. Easty)

Cerebrospinal fluid (CSF) and plasma levels of CEA have been measured in 37 patients with progressive metastatic breast cancer, some of whom had neurological abnormalities, and in 14 subjects with non-malignant neurological disease. Eight out of 17 patients with CNS metastases had elevated CSF CEA levels, together with 5/18 patients with disseminated breast cancer but without apparent neurological abnormality. CSF CEA was not detected in any of the 14 patients without cancer. Estimation of CEA in CSF may be a useful adjunct to the diagnosis of metastatic breast cancer involving the central nervous system.

**PSYCHIATRIC MORBIDITY AFTER CHEMOTHERAPY AND RADIOTHERAPY FOR BREAST CANCER.** A. V. M. Hughson, A. F. Cooper, C. S. McArdle, A. R. Russell & D. C. Smith, *Depts of Psychiatry and Surgery, Victoria Infirmary, Glasgow*

Adjuvant chemotherapy after mastectomy may prolong disease-free survival, but there is little information on its quality (British Breast Group, 1976, *Br. Med. J.*, ii, 861). After simple mastectomy, psychiatric morbidity was studied during a trial of adjuvant chemotherapy, patients being randomized to chemotherapy (C), radiotherapy plus chemotherapy (RC) or radiotherapy (R). Morbidity was measured by a self-rating scale, the General Health Questionnaire (GHQ) (Goldberg, 1972; OUP). Two groups of disease-free survivors were compared: Group I (C and RC) and Group II (R).

Six months after operation, GHQ scores (Table) were similar in both groups. At 12 months, scores had risen in Group I but fallen in Group II, the between-group difference being highly significant. Morbidity remained significantly higher in Group I at both 18 and 24 months after operation. Results indicate that psychiatric illness is commoner in patients completing a one-year course of chemotherapy than in those who had radiotherapy one year before, and that the difference is still evident one year later.

Table.—*GHQ scores: mean ± s.e. (No. patients in brackets)*

| | Months post-op. | | | |
|---|---|---|---|---|
| | 6 | 12 | 18 | 24 |
| Group I | 6·7 ± 1·5 (26) | 12·2 ± 2·4 (34) | 6·5 ± 2·4 (27) | 4·9 ± 1·6 (21) |
| Group II | 7·4 ± 3·5 (14) | 2·4 ± 0·8 (18) | 1·4 ± 0·6 (13) | 1·0 ± 0·7 (10) |
| *P* | N.S. | < 0·001 | < 0·05 | < 0·05 |

### POSTERS

**SPECIES VARIATION IN THE METABOLISM AND EXCRETION OF BENZO(A)PYRENE.** J. K. Chipman, P. C. Hirom, G. S. Frost & P. Millburn, *Department of Biochemistry, St Mary's Hospital Medical School, London*

In the course of a study on the metabolism and enterohepatic circulation of benzo(a) pyrene (BP), the excretion of radioactivity after i.v. administration (3 $\mu$mol/g) of [$^{14}$C]-BP was measured in rabbits and rats. The % dose excreted *via* the bile in 6 h was 35% (rabbit) and 55% rat). The urine was a relatively minor excretory route in both species. Bile samples (2 h) were incubated with $\beta$-glucuronidase, releasing ~40% of the $^{14}$C into a form extractable with ethyl acetate at pH 7. The extracts were analysed by reversed-phase HPLC. Some 30% of $^{14}$C consisted of polar metabolites (possibly polyhydroxylated and/or conjugated). In the rabbit the major aglycone (~35% of extracted $^{14}$C) was identified as BP 9,10-diol. Phenols and quinones accounted for < 15%. In rat bile extracts, however, only low levels of the 9,10-diol were found (< 3%) and the majority of $^{14}$C cochromatographed with phenols and quinones (BP 3,6-quinone comprising up to 30%) and with the 4,5-diol (15%). Trace amounts of $^{14}$C from both species cochromatographed with the 7,8-diol and with BP itself. Whether or not this marked species difference in biliary metabolites of BP is of toxicological significance is uncertain. It is clear, however, that the proximate carcinogen (BP 7,8-diol) is not excreted *via* the bile in appreciable quantities either directly or as a glucuronide in either species.

**USE OF THE CANCER REGISTRY IN THE STRATEGY OF CLINICAL TRIALS IN LUNG CANCER THERAPY.**
C. R. GILLIS*, P. BOYLE*, D. J. HOLE* & I. MCHATTIE†, **West of Scotland Cancer Surveillance Unit, Ruchill Hospital and the* †*Institute of Radiotherapeutics and Oncology, Western Inf., Glasgow*

Cancer registries which use their collected data, particularly those derived from studies of aetiology, can sometimes comment usefully on prospects for trials of therapy. Data derived from a clinical study of immunotherapy, a retrospective case/control study of cigarette-smoking habits and a prospective population study of risk factors for chest and heart disease, have been utilized to obtain a representative sample of 513 cases of lung cancer which, using the cancer registry, have had their outcome of therapy assessed. Treatment information was abstracted from their case records and categorized by presence or absence of radiotherapy, chemotherapy, surgery and immunotherapy. Last known survival was available from existing case records if the patient had not been notified to the cancer registry as having died. The most important finding requiring clarification by prospective study was the apparent similarity of the "no-treatment" group to all other modalities. While the practice of chemotherapy enhanced survival for patients treated by surgery or radiotherapy, the differences were small. This suggests consideration of the use of cancer registries with accurate data to assess the outcome of therapy when the time from diagnosis to death is short.

**ULTRASTRUCTURAL CHANGES IN THE UROTHELIUM OF RATS ADMINISTERED N-BUTYL-N-(4-HYDROXYBUTYL)NITROSAMINE (BBN).**
E. D. MASSEY, A. E. HARVEY, J. CHOWANIEC & R. M. HICKS, *Middlesex Hospital Medical School, London*

BBN induces large exophytic lesions in the rat bladder which occur mainly in the dome and median areas and frequently obstruct the lumen. Well-differentiated transitional-cell tumours were observed macroscopically from 12 weeks onwards, with frequent infiltration into the core of the lesion. As time progressed, the mature, flat hexagonal pavement-like superficial cells with angular foldings and ridges of normal luminal membrane were replaced by immature cells of irregular size and shape with a flexible luminal membrane. These cells were often covered by abnormally dense stubby and/or pleomorphic club-ended microvilli. The latter are thought to be indicative of malignant transformation. Capillary endothelial cell processes were frequently seen to breach the basal lamina and approach the urothelium. This may be related to the development of the extensive capillary network in these rapidly growing tumours. There were many abnormalities at the urothelial-mesenchymal junction: micro-invasion by epithelial cell processes through the basal lamina; loss, fragmentation and invagination of basal laminae into the epithelium and fusion of the basal laminae of blood capillaries and urothelium. There was frequent invasion of the sub-mucosa by basal-cell pseudopodia and multicellular processes attached to the main epithelium by a narrow neck. As the tumours increased in size these lesions were observed more frequently. Their role in carcinogenesis and rapidly growing epithelia was discussed.

**AN ASSOCIATION BETWEEN PARITY AND AGE OF PRESENTATION IN HUMAN BREAST CANCER.**
S. R. SMITH, K. L. WOODS, J. M. MORRISON* & A. HOWELL, *Departments of Medicine, Therapeutics and Clinical Pharmacology, University of Birmingham, and *Division of Surgery, Selly Oak Hospital, Birmingham.*

Early first pregnancy has been shown to exert a protective effect against the development of breast cancer. It has been inferred from this that the initiation of breast cancer occurs early in a woman's reproductive life, and that the latency from initiation to presentation often span several decades. Although the latency cannot be directly measured, any factor which markedly reduces it would tend to result in an earlier age of presentation. We have therefore studied a consecutive series of 371 breast-cancer patients, presenting to a single clinic over a 5-year period, with regard to age of presentation and reproductive history.

The sample was closely representative of all breast-cancer patients presenting in the West Midlands. The nulliparous women were found to be significantly older at presentation (mean age 63·1 years) than the parous women (mean age 57·9 years; $P<0{\cdot}001$). When the parous group was subdivided according to the number of pregnancies, there was a progressive fall in the mean age at presentation as parity increased. In the higher parity groups there was a significant excess of patients whose age at presentation was below the median age for the group as a whole.

We conclude therefore that in a proportion of patients the development of early neoplastic breast lesions is accelerated by pregnancy, and the effect of several pregnancies may be cumulative. These results point to a dual effect of pregnancy. Early first pregnancy reduces the risk of the initiation of breast cancer, but when initiation has occurred the endocrine changes of pregnancy may accelerate subsequent development.

**STABILIZING EFFECT OF NANDROLONE DECANOATE (DECA-DURABOLIN, ORGANON) ON PLATELET FUNCTION IN PATIENTS UNDERGOING CYTOTOXIC THERAPY FOR BREAST CANCER.** J. A. Double, M. C. Bibby, M. Mughal & R. L. Turner, *Postgraduate School of Studies in Medical and Surgical Sciences, University of Bradford*

It has been reported that simultaneous administration of an anabolic steroid with cytotoxic therapy for advanced breast cancer reduced the toxic effects of the therapy on marrow without apparently influencing antitumour activity (Whyte *et al.*, 1959, *Br. Med. J.*, i, 1315; Turner *et al.*, 1979, *Exp. Haematol.*, **7**, Suppl. 6, 52). In order to give the maximum dose of cytotoxic therapy it is essential fully to monitor haemopoiesis. One of the major complications of cytotoxic therapy is severe thrombocytopenia and impaired platelet function leading to harmorrhage. The present study reports the findings from a series of patients undergoing cytotoxic therapy for advanced breast cancer, half of whom received treatment with an anabolic steroid, nandrolone decanoate (ND). The latter was administered 200 mg weekly 14 days before and throughout cytotoxic therapy for a period of 10 weeks. Platelet aggregation was measured with several inducing agents at presentation and at weekly intervals throughout the study. At presentation some patients had impaired platelet function. This function was restored after 1 week in patients receiving ND. Platelet function was maintained in most patients receiving ND throughout the course of cytotoxic therapy. Patients on cytotoxic therapy alone displayed inconsistent platelet aggregation patterns.

**INVESTIGATIONS INTO THE MODULATION OF THYMIDINE (TdR) SALVAGE BY 5-ALKYL DEOXYURIDINES.** S. E. Barrie, A. Jeney, G. A. Taylor & K. R. Harrap. *Dept of Biochem. Pharmacol., Inst. of Cancer Res., Sutton, Surrey*

The effectiveness of antipyrimidine antimetabolites might be enhanced by the concurrent administration of compounds able to modulate selectively the utilization of preformed pyrimidines by tissues; either by prohibiting in tumours, or by enhancing in normal tissues, the "salvage" of preformed pyrimidine. A series of 5-alkyl-substituted deoxyuridines has been studied in relation to the effects of these derivatives on relevant pyrimidine biosynthetic pathways in Ehrlich ascites cells. 5-Ethyl deoxyuridine (EUdR) proved to be the most cytotoxic of the series ($ID_{50} \sim 10^{-5}$M). It was also the most effective at inhibiting thymidine kinase, for which it is a competitive substrate ($K_m$ 70 $\mu$M). Intracellularly it is further converted to higher phosphorylated derivatives and is incorporated at low levels into DNA. It appears to have two likely loci of action: inhibition of thymidylate synthetase by its mononucleotide and inhibition of TdR triphosphate incorporation into DNA.

An interesting tissue-specific property of the series proved to be their competitive inhibition of TdR phosphorylase. This enzyme is present in high concentrations in gastrointestinal mucosa and liver ($K_i$ values for EUdR being 0·6M and 5·2mM respectively) but is virtually undetectable in Ehrlich cells.

When EUdR (400 mg/kg) was administered to tumour-bearing animals it converted slowly to the parent base, accompanied by a 10-fold elevation of TdR in the ascitic fluid. These observations suggest that EUdR or other congeners may be of value in modulating circulating pyrimidine levels, and thus influencing the selective tissue toxicity of anti-pyrimidines.

### RECENT BIOCHEMICAL AND CHEMICAL RESULTS WITH 4-(BIS-(2-BROMO-PROPYL)AMINO)-2′-CARBOXY-2-METHYLAZOBENZENE (CB 10-252).

D. E. V. WILMAN, *Dept of Biochem. Pharmacol., Inst. of Cancer Res., London*

CB 10-252 was designed specifically for the treatment of primary hepatocellular carcinoma (Connors *et al.*, 1973, *J. Natl Cancer Inst.*, **50,** 243). It is chemically unreactive, but 4-(bis-(2-bromopropyl)amino)aniline, produced by reduction of the azo bond, is a potent alkylating agent with a half-life of 43 sec. The requisite enzyme, an azoreductase, is present in liver and in hepatic tumours (Autrup *et al.*, 1974, *Biochem. Pharmacol.*, **23,** 2341). In view of its short half-life the active metabolite is rapidly detoxified upon entry into the circulation. Azoreductase activity has been measured in a variety of human tumours of non-hepatic origin, carried in immune-deprived mice. Enzyme activity ranged between 9 and 56% that of liver. Preliminary investigation has demonstrated a lack of response to the drug by these tumours *in vivo*. It is inferred that CB 10-252 is likely to exhibit anti-tumour effects only in tumours possessing azoreductase activity at least comparable to that of liver.

Further chemical investigation of CB 10-252 has shown it to be a mixture of two components, separable by preparative HPLC, which proved to be diastereoisomers. Chloroform solutions exhibit trans-cis photoisomerism and a thermal cis-trans reaction in the dark. The configuration of the azo group does not appear to affect the ability of azoreductase to reduce the double bond.

CB 10-252 is presently undergoing Phase I/II clinical trial in Lusaka.

### ENZYMATIC ACTIVATION OF ADENINE ARABINOSIDE.

A. BAXTER, H. PARTON & J. P. DURHAM, *Department of Clinical Oncology, University of Glasgow*

There has been increasing interest in the use of adenine arabinoside (Ara-A) as an anti-tumour agent with the development of effective inhibitors of the inactivating enzyme adenosine deaminase (reviewed by Le Page & Khaliq, 1979, *Adv. Enz. Regul.*, **17,** 437). The anti-tumour activity of Ara-A is generally ascribed to the inhibition of DNA polymerase by the nucleoside triphosphate. Activation of Ara-A initially requires a nucleoside kinase, and several cellular nucleoside kinases, namely adenosine kinase, deoxyadenosine kinase and deoxycytidine kinase, have the capacity to phosphorylate adenine nucleosides. Using calf thymus as a control tissue, we have shown that Ara-A is phosphorylated mainly by deoxyadenosine kinase, an enzyme activity readily identifiable by polyacrylamide-gel electrophoresis. This enzyme has also been demonstrated in a porcine lymphosarcoma, and its presence may be important in determining the responsiveness of a tumour to Ara-A therapy. Some properties of deoxyadenosine kinase have been elucidated, including the possibility that its activity *in vivo* is regulated by another cellular protein.

### HEALTH EDUCATION IN THE PRIMARY SCHOOL, WITH SPECIFIC REFERENCE TO SMOKING.

A. FARRELL, A. L. H. CALMAN & K. C. CALMAN, *Department of Clinical Oncology, University of Glasgow*

Over the past 2 years a health-education programme has been in production for use in primary schools in Glasgow, its aim being to introduce early cancer education through smoking prevention and dietary knowledge. Twenty primary schools are at present running the trial programme with their primary 6/7 classes (10–11 years). A further 20 schools are involved in the teacher assessment, giving the following sample:

| | Experimental group | Control group |
|---|---|---|
| Number of children involved | 500 | 500 |
| Number of teachers involved | 225 | 225 |

Evaluation aimed at teachers, parents, children and also at the effectiveness of the teaching material has been carried out at all stages of the research.

Preliminary results of a pilot study carried out in 1978/79 in 3 schools showed that out of a total sample (experimental and control) of 155 children aged 9 years, almost 70% of these had smoked at some point, of whom 55% had smoked only experimentally, but 15% were smoking regularly. This session 1979/80, it is anticipated, with the increased sample number, to finalize the figure obtained in the pilot study regarding the smoking behaviour of children of this age (10–11 years), and to evaluate further any behaviour change brought about as a result of the teaching programme. The teaching programme itself is expected to undergo modification in the light of results obtained from teacher evaluation.

**TUMOUR - DERIVED METABOLIC MODULATORS.** A. W. McNair & K. C. Calman, *Department of Clinical Oncology, University of Glasgow*

A cell-free preparation from TLX-5 lymphoma cells has been shown to affect mouse liver metabolism (McAllister & Galman, 1975, *Br. J. Surg.*, **62**, 161). Recent studies indicate the presence of a factor in the tumour cell-free preparation responsible for raising hepatic citrate content. This factor has been found to be: (i) destroyed by heat-treatment, (ii) non-dialysable, (iii) precipitated by ammonium sulphate fractionation, (iv) present after ultracentrifugation at 240,000 *g* for 24 h, (v) present in the growth medium of TLX-5 cells grown *in vitro*, and shown to be virus free.

The effect of this factor on hepatic citrate content is concentration-dependent. From these observations the factor is likely to be a tumour-derived protein and not a viral contaminant.

**A HUMAN CELL-MEMBRANE ANTIGEN COMMON TO NORMAL TROPHOBLAST AND TO BREAST-CARCINOMA CELLS.** L. C. P. Shah & P. M. Johnson, *Dept of Immunology, University of Liverpool*

Rabbit antisera have been raised against both irradiated human MCF-7 breast-carcinoma cells and against trophoblast microvillous plasma-membrane preparations isolated from normal human placentae. These heteroantisera were then adsorbed with immobilized human term-pregnancy sera, placental alkaline phosphatase and placental ferritin preparations, as well as with human peripheral-blood leucocytes. Immunofluorescence experiments have shown reactivity of both adsorbed antisera with normal trophoblast and with breast carcinoma cells in cryostat tissue sections, but no reactivity with other normal human tissue. Further adsorption experiments have confirmed the specificity of cross-reaction of these antisera with an antigen common to both normal trophoblast and breast-carcinoma cells, and have also shown that the cross-reactive antigen is represented on human Jar choriocarcinoma cells and to a lesser degree on human $AV_3$ amniotic cells. Cross-adsorption experiments have shown that this antigen may not be dominant within the antigenic specifiicites of heteroantisera raised against MCF-7 breast-carcinoma cells or isolated trophoblast microvillous plasma-membrane preparations. It is possible that this cell-membrane antigen common to both breast carcinoma cells and normal trophoblast, but not to normal adult tissues, could play a role in maternal immune responses in pregnancy associated with anti-tumour immunity.

**CIRCULATING IMMUNE COMPLEXES AND ASSESSMENT OF TUMOUR RESPONSE IN GESTATIONAL TROPHOBLASTIC NEOPLASIA AND MALIGNANT TERATOMA.** R. H. J. Begent & K. A. Chester, *Department of Medical Oncology, Charing Cross Hospital, London*

It has been suggested that concentrations of circulating immune complexes (CIC) correlate with tumour response and may form clinically useful tumour markers. This has been tested in gestational trophoblastic neoplasia (GTN) and malignant teratoma (MT) by comparison with the tumour markers already available for these tumours. Concentrations of CIC were measured by quantitating IgG precipitated from serum by 2% polyethylene glycol (Poulton *et al.*, 1978, *Lancet*, ii, 72). In GTN, high levels of CIC were found before treatment in 6/23 patients; in 2 of these they fell to normal with a response to chemotherapy, in 1 they remained raised and the tumour failed to respond; in 3 they were persistently raised even though complete response (CR) was achieved. CIC levels became raised during chemotherapy in 7/17 patients in whom they were normal before treatment, although all achieved CR.
In MT, levels of CIC were raised before treatment in 5/10 patients. In 4 they became normal with a response to chemotherapy and, in 1 of these, rose again on relapse. In the remaining patient levels remained raised although CR was achieved. In 4/5 patients with normal levels before chemotherapy, CIC rose during treatment, though CR was achieved in 2, and 2 were continuing to respond.

In 2 tumours where sensitive tumour markers are already available, CIC concentrations correlated with tumour response in some patients. However, results were misleading too often for the test to be of clinical value, and identification of the tumour-related components is needed for more specific assays to be developed.

**T - LYMPHOCYTE LEVELS IN PATIENTS WITH HODGKIN'S AND NON-HODGKIN'S LYMPHOMAS.** S. Kadhim, R. C. Rees, B. W. Hancock, R. Walker & C. W. Potter, *Department of Virology, The University Sheffield Medical School, Sheffield*

The total number of T lymphocytes in the peripheral blood of 24 patients with Hodgkin's lymphoma and 52 patients with non-Hodgkin's lymphoma was compared to that of 81 controls, using spontaneous sheep red-blood-cell rosetting techniques (E-SRBC). The percentage of rosette-forming lymphocytes was estimated for high-affinity T lymphocytes (29°C) and compared with that of low affinity T lymphocytes (4°C).

Hodgkin's and non-Hodgkin's lymphoma patients showed a decreased ability to rosette at 29°C and 4°C when compared with controls, although more rosette-forming cells were seen at 4°C than at 29°C in both normal controls and in lymphoma patients. Further groupings of patients according to age of disease seemed to indicate deviations in the increase of rosette-forming cells, at 29°C to 4°C, compared with increases in controls.

Pre-treatment of lymphocytes with neuraminidase increased the number of low- and high-affinity rosettes in both patients and controls, but this test could not be used to discriminate between cancer and non-cancer subjects.

**INHIBITION OF THE PRODUCTION OF COLONY-STIMULATING FACTOR BY PHAGOCYTOZING NEUTROPHILS.** J. Fletcher, M. A. Philip & G. Standen, *Clinical Haematology Unit, Department of Therapeutics, City Hospital, Nottingham*

A humoral factor, produced by monocytes and macrophages, known as colony-stimulating factor (CSF) is necessary for the growth of granulocyte/macrophage colonies in agar culture, and probably for granulopoiesis *in vivo*. We now report that production of CSF by mononuclear phagocytes is inhibited by a substance released from phagocytosing neutrophils, but not from resting neutrophils. Phagocytosis releases as much inhibitory activity as disruption of the cells by sonication. Inhibitory activity released by $10^6$ neutrophils/ml can be detected after 100-fold dilution. The inhibitor is heat-labile (30 min at 60°C) and non-dialysable. The amount and kinetics of inhibitor released during phagocytosis are the same as transcobalamin, which suggests that both are constituents of the neutrophil's secondary or specific granules.

**ANTIBODY-DEPENDENT AND SPONTANEOUS CYTOTOXICITY IN HODGKIN'S DISEASE AND CONTROL SPLEEN TISSUE.** S. Al Sam, D. B. Jones, S. V. Payne & D. H. Wright, *University Department of Pathology, General Hospital, Southampton*

To date, studies of cytotoxic effector function in Hodgkin's disease have relied mainly on peripheral blood as a source of lymphocytes (Holm *et al.*, 1975, *Clin. Exp. Immunol.*, **21**, 276). Marked changes in lymphocyte subpopulations are however detectable in the spleen before tumour involvement, and have been implicated in the pathogenesis of the disease (Payne *et al.*, 1976, *Clin. Exp. Immunol.*, **24**, 280). We report preliminary results of a study of cytotoxic effector function in HD spleen—measured by $^{51}Cr$ release.

Spontaneous cell-mediated cytotoxicity at an effector:target ratio of 20:1 against the erythromyeloid line K562 is significantly increased (41·4 ± 7·1) in 5 non-involved HD spleens by comparison with control tissue (19·1 ± 1·2; $P < 0{\cdot}01$) and antibody-dependent cytotoxicity against the nucleated target Raji was similarly raised (43·9 ± 9·6/20·5 ± 13·1; $P < 0{\cdot}05$). These increases were not correlated with an increase in $Fc_G$-positive cells.

ADCC against chick red-blood cells, an effector function depleted on Sephadex G10, in parallel with nonspecific esterase-positive macrophages, was marginally though not significantly increased (72·6 ± 12·8 *vs* 60·3 ± 3·3 at an effector : target ratio of 10:1) in HD.

These results imply either the sequestration of lymphocytes capable of spontaneous and antibody-dependent cytotoxicity in the spleen in HD, or enhancement of the effector capacity of an indigenous population of cells by local immunostimulation.